# Identification and Stereoselective Total Synthesis of an Insect Homosesquiterpene from the Clonal Raider Ant *Ooceraea biroi*

**DOI:** 10.1021/acs.jnatprod.5c00656

**Published:** 2025-08-27

**Authors:** Ryan M. Alam, Yoko Nakamura, Stefan Bartram, Nico Ueberschaar, Tim Zetzsche, Yuko Ulrich, Sarah E. O’Connor, Tobias G. Köllner

**Affiliations:** † Department of Natural Product Biosynthesis, 28298 Max Planck Institute for Chemical Ecology, Hans-Knöll-Straße 8, D-07745 Jena, Germany; ‡ NMR/Biosynthesis Group, Max Planck Institute for Chemical Ecology, Hans-Knöll-Straße 8, D-07745 Jena, Germany; § Institute for Inorganic and Analytical Chemistry, Mass Spectrometry Platform, 425954 Friedrich Schiller University Jena, Humboldtstraße 8, D-07743 Jena, Germany; ∥ Lise Meitner Research Group Social Behaviour, Max Planck Institute for Chemical Ecology, Hans-Knöll-Straße 8, D-07745 Jena, Germany

## Abstract

Ants typically demonstrate a high level of complex social behavior that is largely mediated by chemical communication. In recent years, the Clonal raider ant *Ooceraea biroi* has become a promising model system for the study of social behavior in ants. Here we report the profile of extracted volatiles from *O*. *biroi* and, following detection of an α-homofarnesene as the major component, unambiguously confirm its structural identity as (3*Z*,6*E*)-14-methyl-α-farnesene through preparative stereoselective total synthesis.

## Graphical abstract

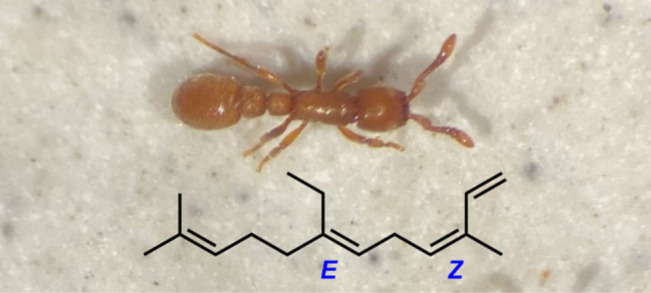

Ants live in complex colonial societies and demonstrate highly sophisticated social behavior that is largely mediated through chemical communication.^1^ Previous efforts to identify and characterize ant-derived semiochemicals have shown that these eusocial insects produce a range of structurally diverse pheromones, that are capable of coordinating activities, such as foraging behavior,^2–8^ alarm response,^9–11^ conspecific recruitment,^12^ mate attraction,^13^ and brood care.^14^ Furthermore, the use of multicomponent pheromone blends, where combinations of compounds are employed to elicit a specific behavioral response, have been noted in several ant species^13,15,16^ and are particularly advantageous, as they enable an even higher degree of communication complexity.^17^

The Clonal raider ant *Ooceraea biroi,* formerly known as *Cerapachys biroi*, has become an attractive model system for examining the genetic and neuronal bases of social behavior,^18–22^ as it lacks a queen caste, reproduces asexually, and is amenable to genetic manipulation.^19,23^ However, apart from the identification of two alarm pheromones, 4-methylheptan-3-one and 4-methylheptan-3-ol,^18^ little is known about its semiochemical composition. Here we present the analysis and unambiguous structural elucidation of extracted volatiles in *O. biroi*.

## Results and Discussion

Gas chromatography-electron ionization-mass spectrometry (GC-EI-MS) analysis of whole-body extracts taken from foraging worker ants permitted the detection of several distinct volatile compounds (Figure 1A). Each peak was tentatively identified using the National Institute of Standards and Technology (NIST) MS-Library v. 3.0 (2023) and then confirmed by comparison with an authentic standard (see Supporting Information), albeit with the exception of later eluting cuticular hydrocarbons (t*_r_* 22.00–23.50 min). We could detect the known alarm pheromones,^18^ 4-methylheptan-3-one (peak 1, t*_r_* 4.79 min) and 4-methylheptan-3-ol (peak 2, t*_r_* 5.32 min), for which the exact stereochemical configurations remain unassigned, and *n*-undecane (peak 3, t*_r_* 7.47 min). Interestingly, at least two other previously unidentified components were also observed, i.e., β-springene (peak 5, t*_r_* 17.69 min) (see Supporting Information), and a major peak, eluting at 13.79 min (peak 4), that gave an EI-MS spectrum which could not be readily identified (Figure 1B). Notably, none of the above-mentioned compounds could be detected in hexane extracts from *O*. *biroi* larvae and pupae (see Supporting Information).

**Figure 1:**
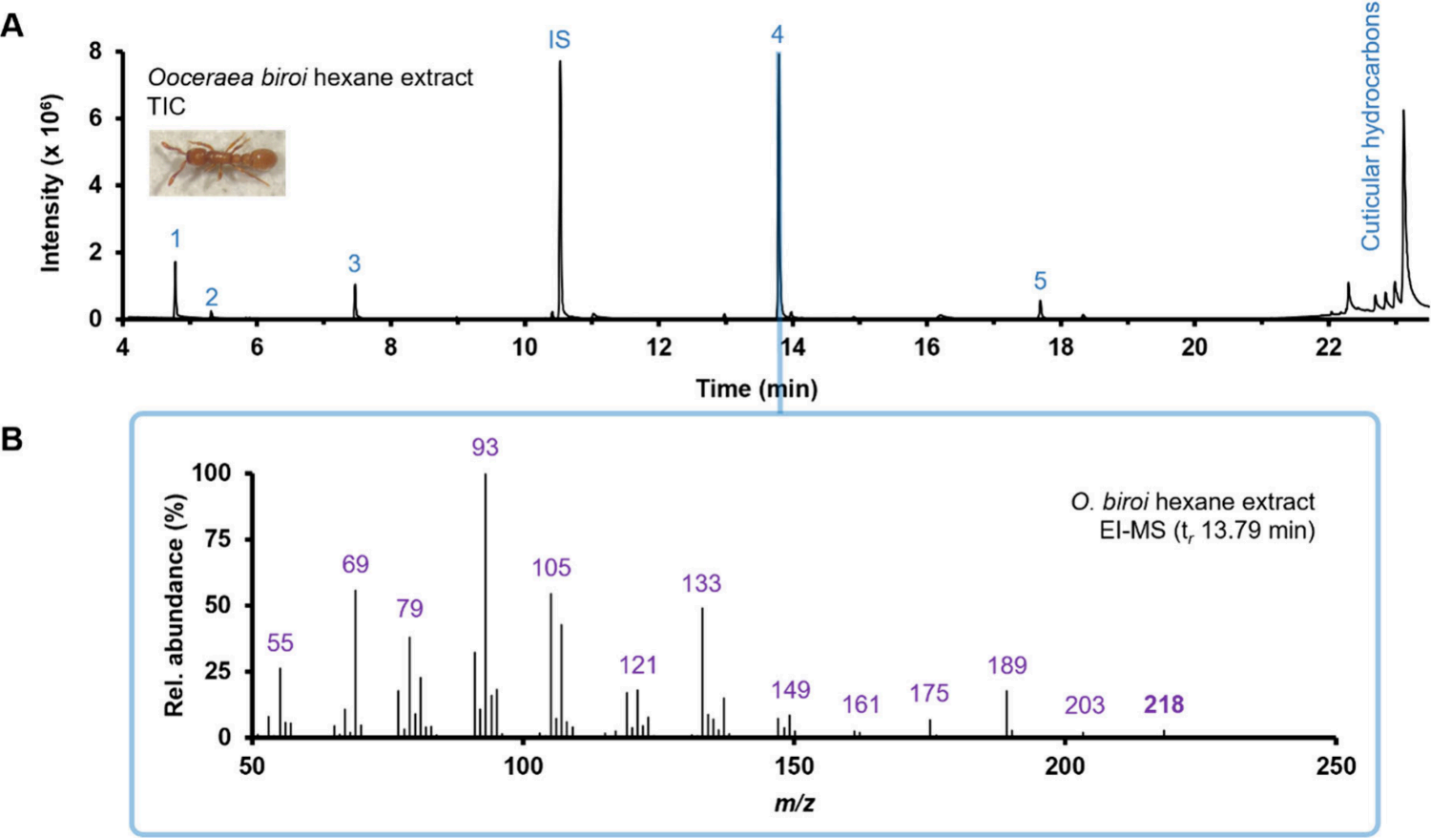
(**A**) GC-EI-MS total ion current (TIC) chromatogram of an *O*. *biroi* whole-body extract. Peak identity: 1) 4-methylheptan-3-one, 2) 4-methylheptan-3-ol, 3) *n*-undecane, 5) β-springene. (**B**) EI-MS spectrum obtained for peak 4 (t*_r_* 13.79 min).

The EI-MS spectrum for peak 4 showed that the compound had a molecular ion of *m*/*z* 218 (Figure 1B). High resolution GC-orbitrap-MS analysis confirmed a molecular formula of C_16_H_26_ (observed [M^+·^] 218.2033, calcd. C_16_H_25_, 218.2029, Δ*_m_*_/_ *_*z*_* 0.4 mDa [1.8 ppm]) with four degrees of unsaturation. Alongside the presence of the characteristic isoprenoid fragment ion *m*/*z* 69 (C_5_H_9_^+·^) as the second most abundant fragment ion (62%), the EI fragmentation pattern for peak 4 suggested the successive loss of at least 11 methyl/methylene substituents, and indicated that peak 4 may be a terpenoid derivative. Considering that peak 4 could be a C_16_-containing homosesquiterpene led to an account from Morgan and Thompson detailing the identification of several terpenoids in myrmicine ants.^24^ Comparison of nominal EI-MS spectra for peak 4 with the tentatively identified pheromone, (3*Z*,6*E*)-14-methyl-α-farnesene (**1b**),^24^ showed a high degree of similarity.

To confirm the identity of peak 4, we aimed to prepare **1** as a mixture of isomers (**1a**–**d**) employing Thompson and Morgan’s reported route (Scheme 1).^24^ However, Wittig olefination of ethyl ketone **2** in the presence of phosphonium iodide **3** ylide was nonproductive in our hands and only gave a complex mixture (see Supporting Information). Conversely, when cyclohexanone was substituted for ethyl ketone **2**, the ylide of **3** was found to proceed within 30 min at room temperature to the corresponding trisubstituted alkene, indicating that the recalcitrant olefination of **2** is impeded by steric hindrance.

**Scheme 1:**
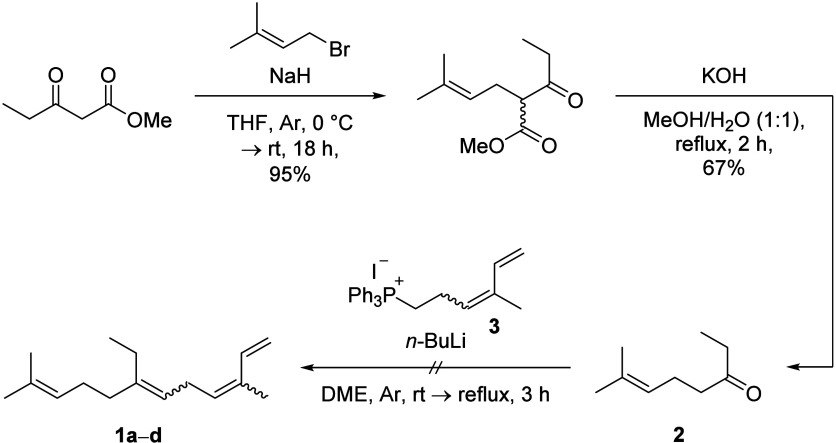
Wittig olefination of ethyl ketone **2** failed to provide access to 14-methyl-α-farnesene isomers **1a**–**d**

Inspired by Ando and Tamaka’s *Z*-selective synthesis of trisubstituted alkenes under Julia-Kocienski olefination conditions,^25^ we aimed to access homosesquiterpene **1b** directly from sulfonyl tetrazole **4**, which could be synthesized over three steps from homoallylic alcohol **5** (Scheme 2).

**Scheme 2:**
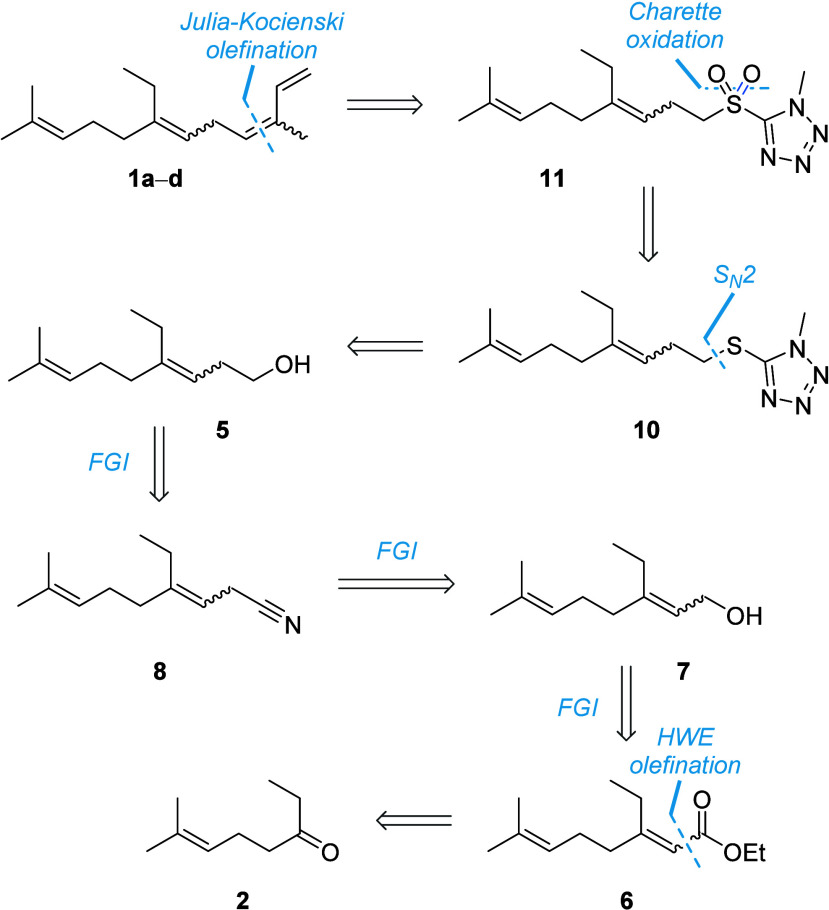
Retrosynthetic analysis of 14-methyl-α-farnesene isomers **1a**–**d**. Note: FGI = functional group interconversion

Ester **6** was prepared as a mixture of isomers (ratio *E*:*Z* = 3:2; 83% yield) via Horner-Wadsworth-Emmons (HWE) olefination of ethyl ketone **2** (Scheme 3) and then reduced to the corresponding allylic alcohol **7** (64% yield) in the presence of diisobutylaluminum hydride (DIBAL-H). Phosphorus tribromide-mediated halogenation of **7** followed by cyanation, afforded the corresponding nitrile **8** (71% yield), which was then hydrolyzed under alkaline conditions to furnish β,γ-unsaturated acid **9**. Notably, basic hydrolysis of **8** also generated the corresponding α,β-unsaturated isomer of **9** as a minor side product (*ca*. 10% by ^1^H NMR). With crude **9** in hand, lithium aluminum hydride (LAH) reduction afforded the corresponding homoallylic alcohol **5** as a mixture of (*E*/*Z*)-alkene isomers in 81% yield over two steps. Thioether **10** was prepared from **5** via the corresponding mesylate. Oxidation of **10** to sulfone **11** was achieved employing Charette’s conditions,^26^ using a combination of sodium tungstate and hydrogen peroxide at room temperature (44% yield). Following Ando and Tamaka’s procedure,^25^ olefination of **11** with methyl vinyl ketone (**12**) proceeded nonstereoselectively, as evidenced by GC-EI-MS (Figure 2A) to yield 14-methyl-α-farnesene (**1a**–**d**) as a mixture of stereoisomers (24% yield).

**Scheme 3:**
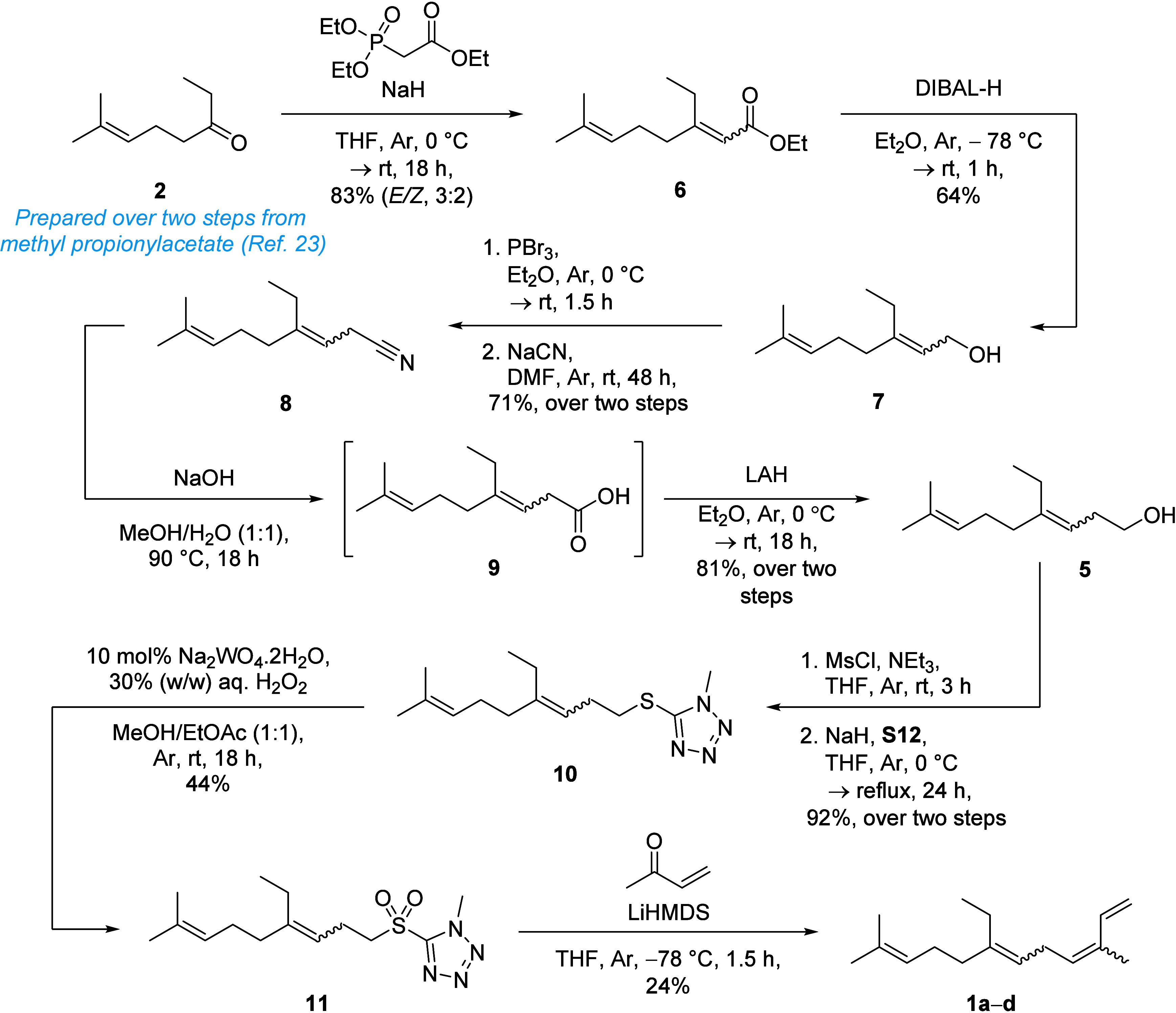
Non-stereoselective synthesis of 14-methyl-α-farnesene isomers **1a**–**d**

**Figure 2:**
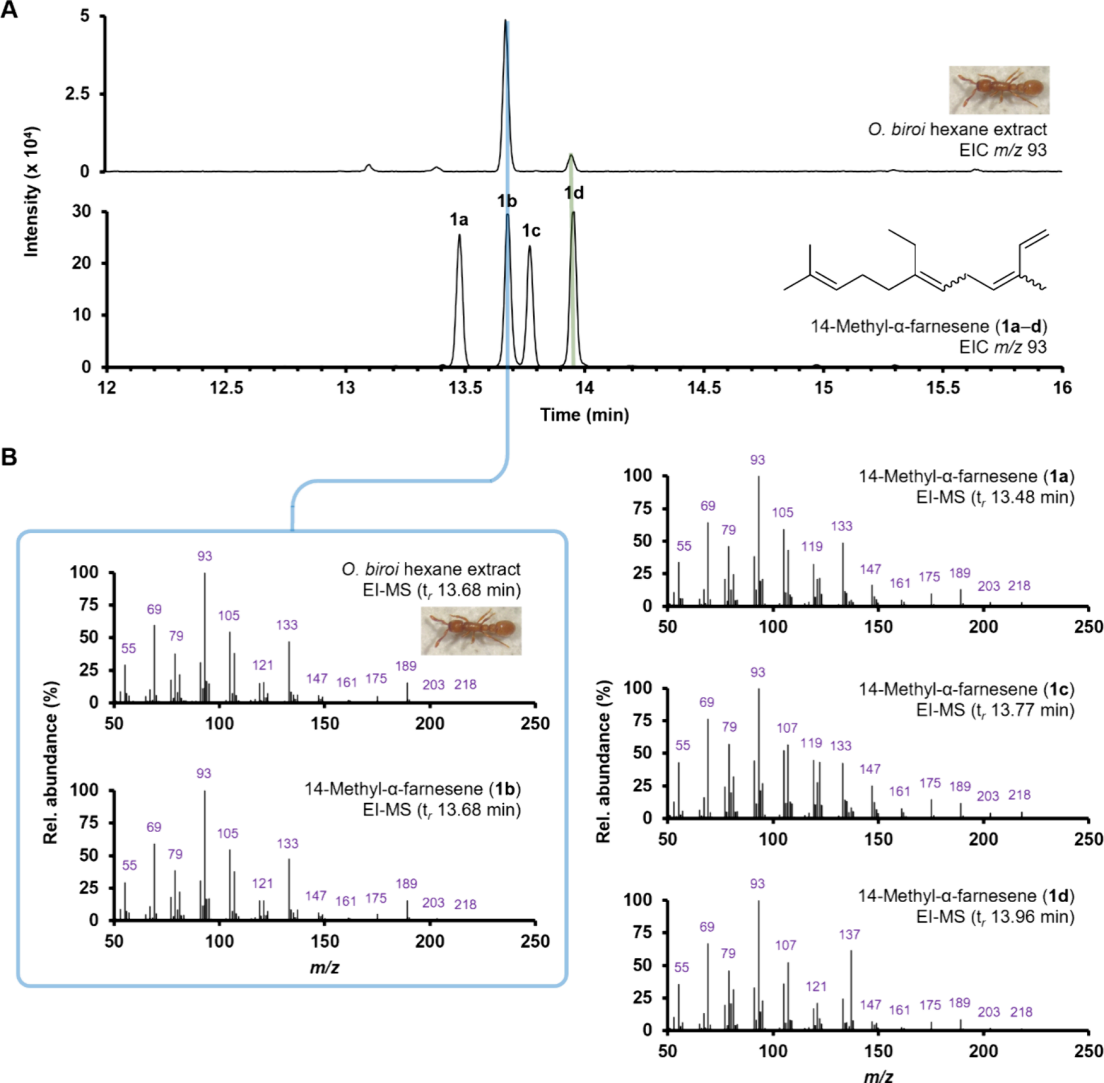
Comparison of GC-EI-MS chromatograms (**A**) and mass spectra (**B**) obtained for an *O*. *biroi* extract (top) and **1a**–**d** (bottom). Column: OPTIMA WAX.

Comparing GC-EI-MS data for peak 4 and the synthetic mixture of 14-methyl-α-farnesene isomers (**1a**–**d**) confirmed that the major component found in *O*. *biroi* was one of four stereoisomers (Figure 2A). The EI-MS spectra for both peak 4 and the coeluting 14-methyl-α-farnesene isomer (t*_r_* 13.68 min) were identical (Figure 2B). Furthermore, comparison of the *O*. *biroi* extract with the mixture of synthetic stereoisomers **1a**–**d** showed that the ant extract also contained an additional 14-methyl-α-farnesene isomer, **1d**, as a minor component (t*_r_* 13.96 min, Figure 2A). With the exception of the fragment ion *m*/*z* 137 observed for **1d**, all four stereoisomers of 14-methyl-α-farnesene (**1a**–**d**) produced similar EI-MS spectra with comparable fragmentation patterns (Figure 2B). Importantly, homofarnesene isomers **1a**–**d** were separable using both polar (OPTIMA WAX) and nonpolar (ZB-5) stationary phases.

Although the elution order of isomers **1a**–**d** could not be unambiguously determined (Figure 2A), inspection of the relative ratios of (*E*)- and (*Z*)-alkene isomers present in sulfonyl tetrazole precursor **11** and resulting mixture of stereoisomers **1a**–**d**, using ^1^H NMR suggested that the major isomer present in *O*. *biroi* was (3*Z*,6*E*)-14-methyl-α-farnesene (**1b**). Moreover, comparing chromatographic data obtained for **1a**–**d**, using a polar stationary phase (OPTIMA WAX), with structurally similar sesquiterpenes, i.e., (2*Z*,6*Z*)-, (2*Z*,6*E*)-, (2*E*,6*Z*)-, and (2*E*,6*E*)-farnesol, separated using a similar PEG-based stationary phase (ZB-FFAP),^27^ provided further support for our tentative stereochemical assignment of peak 1a (t*_r_* 13.68 min, Figure 2A).

As mentioned earlier (*vide supra*), **1b** has been found in certain myrmicine ant species, based on detailed nominal GC-EI-MS^24,28,29^ or infrared (IR) spectroscopic analysis.^30^ However, to unequivocally determine the structure of the isomer present in *O*. *biroi* and access material for future bioassay we aimed to identify a preparative stereoselective synthetic route to **1b**. Importantly, as **1a**–**d** were inseparable using flash column and/or preparative thin layer chromatographic techniques, it was imperative that **1b** be prepared stereoselectively. Retrosynthetic analysis indicated that **1b** may be approached through methylenation of β,γ-unsaturated aldehyde **13**. Importantly, (2*Z*,5*E*)-methyl ester **14** could be prepared from aldehyde **15**, employing a *Z*-selective Still-Gennari olefination strategy which would proceed via the kinetically favorable *cis*-oxaphosphetane adduct **16** in the presence of phosphoryl ester **17** (Scheme 4).^31^

**Scheme 4:**
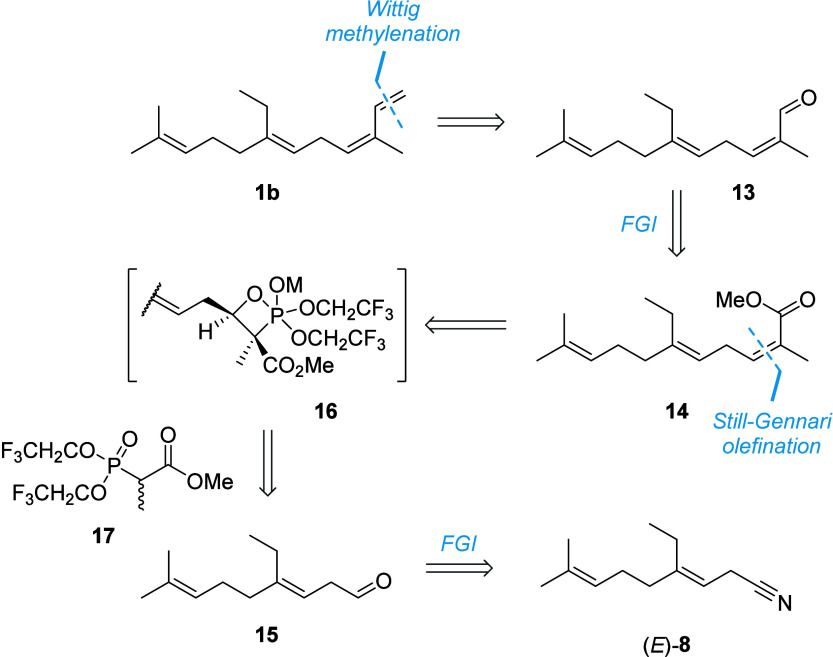
Retrosynthetic analysis of (3*Z*,6*E*)-14-methyl-α-farnesene (1b)

Allylic alcohol (*E*)-**7** was obtained following Gibb’s route^32^ in 37% yield over four steps. With (*E*)-**7** in hand, nitrile (*E*)-**8** could be prepared stereoselectively over (62% yield over two steps), via cyanation of the corresponding bromide in the presence of tetrabutylammonium cyanide (Scheme 5).^33^ However, notwithstanding the use of several distinct reaction conditions, including diisobutylaluminum hydride (DIBAL-H) in various solvents, LiAl­(OEt)_3_H,^34^ and NaH/ZnCl_2_,^35^ attempts to access homoallylic aldehyde **15** from (*E*)-**8** were unsuccessful.

**Scheme 5:**
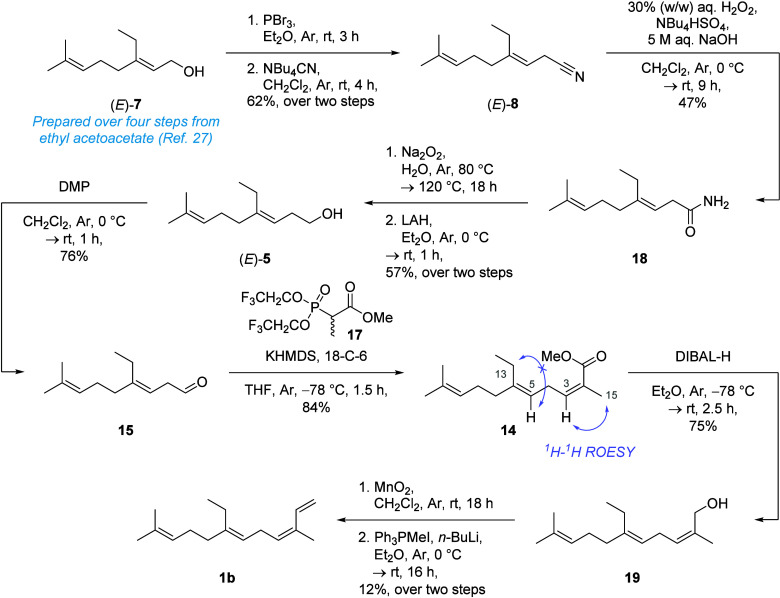
Stereoselective synthesis of (3*Z*,6*E*)-14-methyl-α-homofarnesene (1b)

Alongside the formation of the corresponding α,β unsaturated acid side product (*vide supra*), alkaline hydrolysis of nitrile (*E*)-**8** yielded the corresponding acid (*E*)-**9** as a mixture of inseparable 3*E*/*Z*-alkene isomers. Alternatively, (*E*)-**8** was successfully hydrolyzed to primary amide **18** in the presence of basic hydrogen peroxide under phase-transfer conditions^36^ (47% yield). Subsequent, treatment of β,γ-unsaturated amide **18** with sodium peroxide^36^ afforded the corresponding acid which was directly reduced to alcohol (*E*)-**5** without any observable alkene isomerization. Dess-Martin oxidation of (*E*)-**5** then readily permitted access to desired aldehyde intermediate **15**.

Still-Gennari olefination^37^ of homoallylic aldehyde **15** with ester **17** ^38^ stereoselectively gave the desired (3*Z*,6*E*)-isomer **14** (84% yield). Stereochemical confirmation of **14** was obtained via observation of a positive ^1^H–^1^H rotating frame Overhauser enhancement spectroscopy (ROESY) correlation between H-3 and H-15, alongside no detectable correlation between H-5 and H-13 (see Supporting Information). The DIBAL-H-mediated reduction of **14** afforded allylic alcohol **19** (75% yield) which was then oxidized in the presence of manganese dioxide to yield α,β-unsaturated aldehyde **13**. However, **13** underwent spontaneous (*E*/*Z*)-alkene isomerization upon standing at room temperature and was therefore immediately carried forward to the final reaction step. Finally, Wittig methylenation of labile aldehyde **13** furnished (3*Z*,6*E*)-14-methyl-α-farnesene (**1b**) in 12% yield over two steps.

Comparison of GC-EI-MS data for the *O*. *biroi* extract and synthetic **1b** showed that both compounds coelute (t*_r_* 13.68 min) and share identical EI-MS spectra, thus confirming the absolute stereochemistry of the major volatile terpenoid present in *O*. *biroi* (Figure 3). Interestingly, (*E*,*E*)-14-methyl-α-farnesene (**1d**) was also detected in synthetic **1b** and coeluted with the previously observed minor α-homofarnesene isomer **1d** (t*_r_* 13.95 min). The presence of minor amounts of **1d** (relative to **1b** in our synthetic standard) likely arises from the undesirable (2*Z*)- to (2*E*)-alkene isomerization of α,β-unsaturated aldehyde **13** (*vide supra*) prior to Wittig methylenation (Scheme 5).

**Figure 3:**
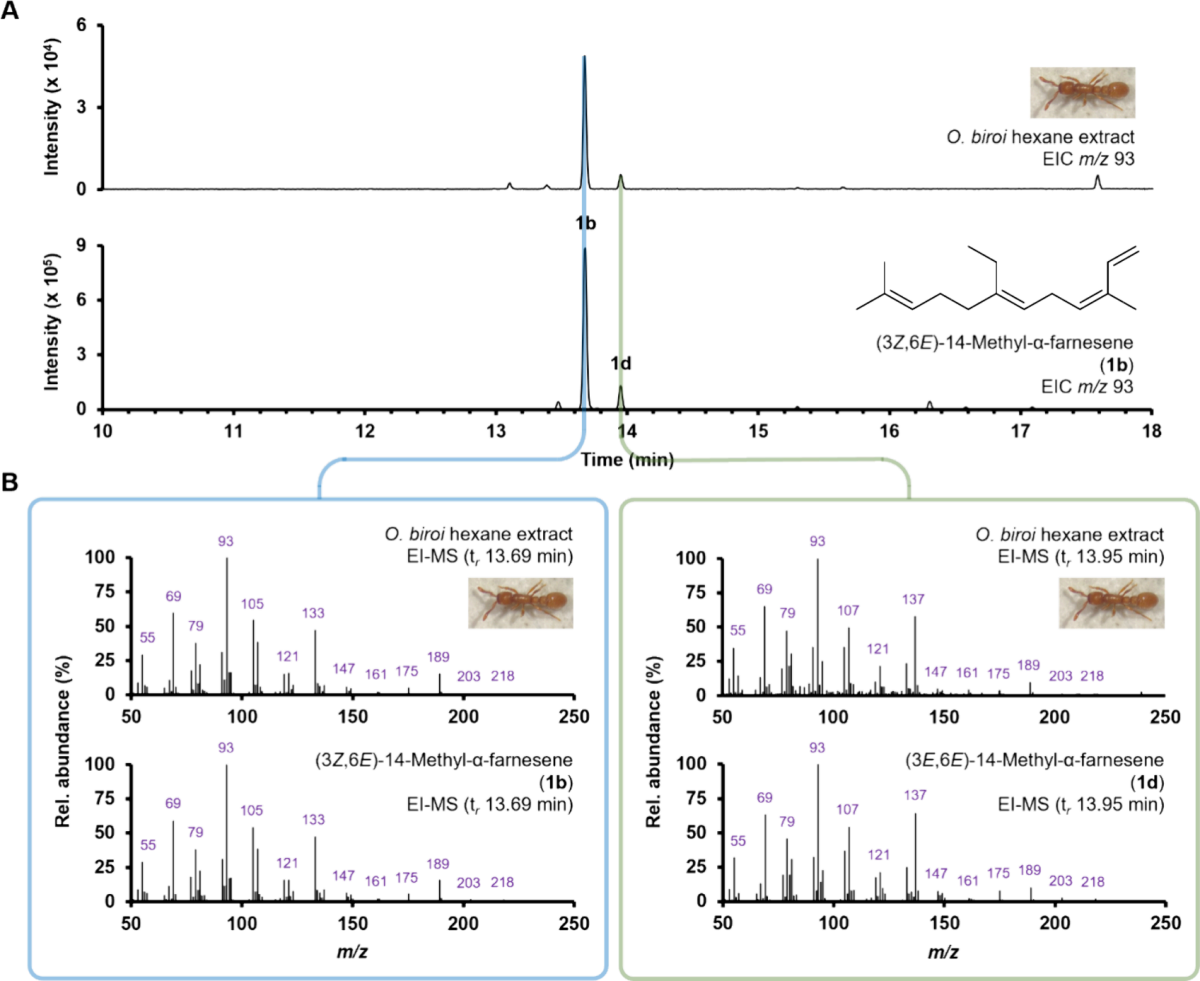
Comparison of GC-EI-MS chromatograms (**A**) and mass spectra (**B**) obtained for an *O*. *biroi* extract (top) and synthetic **1b** (bottom, left; containing **1d** [bottom, right]).

Notably, several homosesquiterpenes have been found in the sandfly *Lutzomyia longipalpis*, including 9-methylgermacrene-B^39,40^ and 3-methyl-α-himachalene,^41,42^ which act as sex pheromones. However, given the structural similarity of **1b** with homoterpenoid trail pheromones found in other ants,^43^ such as *Solenopsis invicta*,^44^ and its observed absence in both pupae and larvae (see Supporting Information), we suspect **1b** may function as a trail pheromone component in *O*. *biroi* workers.

In summary, alongside the detection and identification of β-springene, GC-EI-MS has revealed that (3*Z*,6*E*)-14-methyl-α-farnesene (**1b**) is the major terpenoid present in the Clonal raider ant. Through stereoselective total synthesis of terpenoid **1b**, we have unequivocally confirmed its structure and gained access to material that will support ongoing studies that aim to determine its biological role(s) in *O*. *biroi*.

## Experimental Section

### General Experimental Note

All reagents were obtained from commercial sources and used without any further purification unless otherwise specified. Room temperature ranged between 18–22 °C. Both thin layer chromatography (TLC) and preparative TLC (PTLC) were carried out on aluminum-backed silica gel 60 F_254_ plates (Merck) that were visualized with Hanessian’s stain, alkaline KMnO_4_, and/or using UV_254 nm_ light detection. Gradient flash column chromatography was performed using Merck silica gel 60 (particle size 0.040–0.063 mm, density 0.8 g/cm^3^).

### Ant Extract Preparation

Individual specimens of *Ooceraea biroi* workers were taken from colonies kept in darkness at 27 ± 1 °C, prior to extraction in hexane (200 μL). After standing at room temperature for 30 min, the hexane extract was taken off with a glass micropipette and directly submitted for GC-EI-MS analysis using an injection volume of 1 μL.

### Nuclear Magnetic Resonance (NMR) Spectroscopy

Nuclear magnetic resonance (NMR) spectra were recorded on Bruker Avance III HD 400 or 500 MHz spectrometer (Bruker Biospin GmbH, Rheinstetten, Germany) at room temperature (*ca*. 293 K), using CDCl_3_ as sample solvent. Chemical shift values (δ_H_ and δ_C_) are reported in parts per million (ppm) relative to solvent (δ_C_ 77.0 [*C*DCl_3_]) or residual solvent (δ_H_ 7.26 [C*H*Cl_3_]) and coupling constants (*J*) are expressed in Hertz (Hz), in the following format: chemical shift value (multiplicity, coupling constant, integration). ^1^H NMR spectral data are described, using the following abbreviations; s (singlet), brs (broad singlet), d (doublet), t (triplet), q (quartet), appd (apparent doublet), dd (doublet of doublets), dq (doublet of quartets), apptsept (apparent triplet of septets), qt (quartet of triplets), ddd (doublet of doublets of doublets), and m (multiplet).

### Gas Chromatography-Electron Ionization-Mass Spectrometry (GC-EI-MS)

Nominal gas chromatography electron ionization mass spectrometry (GC-EI-MS) analyses were performed using an Agilent 8890 Series gas chromatograph coupled with an Agilent 5977B single quadrupole mass selective detector (Agilent Technologies, CA 95051, United States). Unless stated otherwise, samples were prepared using dichloromethane as sample solvent to give a final concentration of ca. 10 μg/mL. Chromatographic analyses were performed using helium as the carrier gas applied at a constant flow rate of 1.1 mL/min with an injection volume of 1 μL in splitless mode and were all carried out using a PAL RSI 120 autosampler (CTC Analytics AG, Zwingen, Switzerland). The injector and transfer line temperatures were 220 and 280 °C, respectively. For chromatographic separation, an initial column oven temperature of 60 °C was held for 2 min and then increased by 10 °C/min to 250 °C, prior to being heated to 320 °C (100 °C/min ramp) and held at 320 °C for 2 min, using a Zebron ZB-5 column (5% phenyl, 95% dimethylpolysiloxane; 30 m length, 0.25 mm inner diameter, 0.25 μm film thickness, 10 m precolumn, Phenomenex, Aschaffenburg, Germany). Alternatively, chromatographic separations using an OPTIMA WAX column (polyethylene glycol [CW 15–20 kDa]; 30 m length, 0.25 mm inner diameter, 0.25 μm film thickness; Macherey-Nagel, Düren, Germany) were carried out employing an initial column oven temperature of 40 °C that was held for 2 min and then heated to 240 °C (10 °C/min ramp) and subsequently maintained at 240 °C for 3 min. Solvent delay was set to 4 min. The ion source temperature was 230 °C. MS data acquisition was carried out in scan mode (mass range, 50–450 *m*/*z*). The ionization energy was 70 eV.

### High-Resolution Mass Spectrometry (HRMS)

General high-resolution mass spectrometry (HRMS) experiments were performed using an Impact II ultrahigh-resolution quadrupole-time-of-flight (UHR-Q-ToF) (Bruker Daltonik; Bremen, Germany) mass spectrometer operating in positive electrospray (ESI) mode, unless otherwise specified. Liquid sample introduction was achieved with an UltiMate 3000 ultrahigh performance liquid chromatography (UHPLC) system (ThermoFisher Scientific, Germering, Germany), employing an injection volume of 2 μL that was introduced directly to the ion source without any prior chromatographic separation. The solvent system employed for HRMS analysis consisted of H_2_O, containing 0.1% (v/v) formic acid, and acetonitrile (1:1) that was applied at a flow rate of 0.2 mL/min at 24 °C with a total runtime of 2 min. Samples for analysis were prepared using acetonitrile as solvent, to give a final sample concentration of approximately 0.1 mg/mL. The desolvation gas (dry N_2_) temperature was maintained at 200 °C with a flow rate of 9 L/min and the operating capillary voltage and end plate offset were 3.5 kV and 0.5 kV, respectively. The acquisition mass range was 100–500 *m*/*z*, with a sampling rate of 1 Hz. External calibration was performed using a solution of sodium formate in *i*-PrOH that was introduced via syringe pump with a flow rate of 0.18 mL/h for the first 0.02 min of the run.

Alternatively, samples dissolved in dichloromethane or hexane were injected via a TriPlus RSH autosampler (Thermo Fisher Scientific, Bremen, Germany) on a TRACE 1310 gas chromatograph (Thermo Fisher Scientific, Bremen, Germany). For liquid samples, the SSL injector in split mode (1:10 split flow) at a temperature of 300 °C, and a gas flow of 1 mL/min, was employed. Helium 5.0 was used as carrier gas with an additional moisture and oxygen trap (the vendor specifies a gas quality of 6.0 after passage), on a Zebron ZB-SemiVolatiles column (5% phenyl, 95% dimethylpolysiloxane; 30 m length, 0.25 mm inner diameter, 0.25 μm film thickness, 10 m precolumn, Phenomenex, Aschaffenburg, Germany). The initial oven temperature of 40 °C was held for 2 min, and then increased to 320 °C at a rate of 10 °C/min. This temperature was kept for 5 min. The TRACE 1310 gas chromatograph was coupled with a Q Exactive GC mass spectrometer (Thermo Fisher Scientific, Bremen, Germany). The resolution was set to 120,000 (FWHM) throughout analysis and the mass range was set to 50–650 *m*/*z* for measurements. Automated gain control (AGC target) was set to 1 × 10^6^, and maximum inject time was set to “auto”. Auxiliary temperatures were set to 280 °C for both transfer lines 1 and 2. MS transfer line temperature was set to 250 °C and the temperature of the electron ionization source was set to 300 °C. EI was performed at 70 eV energy, a filament delay was set to 5.4 min. Nitrogen for supply of the C-Trap and HCD cell of the GC Orbitrap had a minimum purity of 99.999% (Linde, Munich, Germany), and was further dried, using a moisture filter (the vendor specifies a gas quality of 6.0 after passage; Thermo Fisher Scientific, Bremen, Germany).

### (*E*)-4-Ethyl-8-methylnona-3,7-dienenitrile ((*E*)-8)

To a dry 50 mL round-bottom flask under an Ar atmosphere, was added alcohol (*E*)-**7** (402 mg, 2.39 mmol) and anhydrous Et_2_O (5 mL). Upon cooling to 0 °C, the colorless solution was treated dropwise with PBr_3_ (75 μL, 0.79 mmol) and allowed to warm to room temperature. After stirring for 3 h, the reaction mass was charged with brine (10 mL). Following layer separation, the aqueous phase was extracted with Et_2_O (10 mL x 3). The combined organic layers were dried over anhydrous Na_2_SO_4_ and carefully concentrated under reduced pressure to afford an amber oil that was subsequently dissolved in anhydrous CH_2_Cl_2_ (13.5 mL) and added dropwise to a stirring solution of freshly prepared tetrabutylammonium cyanide^45^ (3.160 g, 11.77 mmol) in anhydrous CH_2_Cl_2_ (10 mL). After stirring for 4 h, the resulting mixture was concentrated in vacuo to give a viscous amber oil that was purified by gradient flash column chromatography to (0–5% Et_2_O in pentane) to furnish title compound (*E*)-**8** as a colorless oil (1.300 g, 62% [over two steps from allylic alcohol (*E*)-**25**]): R*_f_* 0.27 (Et_2_O/pentane, 1:19); ^1^H NMR (CDCl_3_, 400 MHz) δ 5.12 (t, *J* = 7.0 Hz, 1H), 5.08–5.06 (m, 1H), 3.06 (d, *J* = 7.0 Hz, 2H), 2.11–2.03 (m, 6H), 1.69 (s, 3H), 1.60 (s, 3H), 1.01 (t, *J* = 7.6 Hz, 3H); ^13^C NMR (CDCl_3_, 100 MHz) δ 147.8, 132.1, 123.5, 118.8, 111.0, 36.1, 26.3, 25.6, 23.4, 17.7, 15.9, 12.5; HRMS (ESI/Q-ToF) *m*/*z*: [M + H]^+^ Calcd for C_12_H_20_N 178.1590; Found 178.1591 (0.6 ppm).

### (*E*)-4-Ethyl-8-methylnona-3,7-dienamide (18)

To a 50 mL round-bottom flask, containing an ice-cold solution of nitrile (*E*)-**8** (1.3 g, 7.33 mmol) dissolved in CH_2_Cl_2_ (3.2 mL), was sequentially added 30% (w/w) aq. H_2_O_2_ (3.51 mL), tetrabutylammonium hydrogen sulfate (547 mg, 1.61 mmol), and 5 M aq. NaOH (3.2 mL, 2.2 mmol). The mixture was warmed to room temperature and stirred for a further 9 h. The reaction mass was diluted with CH_2_Cl_2_ (10 mL), separated, and the resulting aqueous layer subsequently extracted with CH_2_Cl_2_ (5 mL x 4). The combined organic phases were dried over anhydrous Na_2_SO_4_ and concentrated under reduced pressure to afford a residue that was then purified by gradient flash column chromatography (50–100% Et_2_O in pentane) to yield primary amide **18** as a light yellow oil (673 mg, 47%): R*_f_* 0.10 (Et_2_O); ^1^H NMR (CDCl_3_, 400 MHz) δ 5.79 (brs, 1H), 5.48 (brs, 1H), 5.28 (t, *J* = 7.7 Hz, 1H), 5.08–5.06 (m, 1H), 3.00 (d, *J* = 7.7 Hz, 2H), 2.11–2.03 (m, 6H), 1.67 (s, 3H), 1.60 (s, 3H), 0.99 (t, *J* = 7.6 Hz, 3H); ^13^C NMR (CDCl_3_, 100 MHz) δ 174.3, 147.0, 132.1, 124.1, 116.1, 36.2, 35.0, 26.4, 25.7, 23.0, 17.7, 12.9; HRMS (ESI/Q-ToF) *m*/*z*: [M + H]^+^ Calcd for C_12_H_22_NO 196.1696; Found 196.1696 (0.0 ppm).

### (*E*)-4-Ethyl-8-methylnona-3,7-dien-1-ol ((*E*)-5)

To a 50 mL pear-shaped flask, containing primary amide **18** (670 mg, 3.43 mmol) suspended in H_2_O (10 mL), was slowly added portionwise Na_2_O_2_ (267 mg, 3.43 mmol). Following complete addition of Na_2_O_2_, the mixture was heated to 85 °C (oil bath) for 6 h and then charged with additional Na_2_O_2_ (267 mg, 3.43 mmol) and heated to 120 °C (oil bath) for 12 h. The resulting solution was cooled to 0 °C, adjusted to pH 5 with ice-cold 2 M HCl, and repeatedly extracted with CH_2_Cl_2_ (10 mL x 6). The combined organic layers were dried over anhydrous MgSO_4_ and concentrated *in vacuo* to give an amber oil that was carried forward to the next reaction step without any further purification. To a separate dry 25 mL round-bottom flask under an Ar atmosphere, containing an ice-cold suspension of LiAlH_4_ (325 mg, 8.58 mmol) in anhydrous Et_2_O (10 mL), was added dropwise crude carboxylic acid intermediate (*E*)**-9** in anhydrous Et_2_O (5 mL). The resulting mixture was warmed to room temperature and stir for 1 h, then cooled to 0 °C, and slowly treated dropwise with H_2_O (0.33 mL), 15% (w/v) aq. NaOH (0.33 mL), H_2_O (1 mL). After stirring at room temperature for 30 min, the gray suspension was filtered through a short pad of Celite to give a filtrate that was then dried over anhydrous Na_2_SO_4_ and concentrated under reduced pressure to afford alcohol (*E*)-**5** as a colorless oil (356 mg, 57% [over two steps from **18**]): R*_f_* 0.47 (Et_2_O/hexane, 2:3); ^1^H NMR (CDCl_3_, 500 MHz) δ 5.10–5.06 (m, 2H), 3.61 (t, *J* = 6.4 Hz, 2H), 2.30 (q, *J* = 6.7 Hz, 2H), 2.09–2.03 (m, 6H), 1.69 (s, 3H), 1.60 (s, 3H), 0.98 (t, *J* = 7.6 Hz, 3H); ^13^C NMR (CDCl_3_, 125 MHz) δ 144.9, 131.6, 124.3, 119.3, 62.5, 36.5, 31.1, 26.8, 25.7, 23.2, 17.7, 13.3; HRMS (ESI/Q-ToF) *m*/*z*: [M + H]^+^ Calcd for C_12_H_23_O 183.1743; Found 183.1743 (0.0 ppm).

### (*E*)-4-Ethyl-8-methylnona-3,7-dienal (15)

To a dry 25 mL round-bottom flask under an Ar atmosphere, containing an ice-cold solution of alcohol (*E*)**-5** (282 mg, 1.54 mmol) dissolved in anhydrous CH_2_Cl_2_ (10 mL), was added Dess-Martin periodinane (849 mg, 2 mmol). The reaction mass was warmed to room temperature and stirred for 1 h (complete consumption of starting material (*E*)-**5** was confirmed by TLC [Et_2_O/pentane, 2:3]) and then directly subjected to gradient flash column chromatography (0–5% Et_2_O in pentane) to afford aldehyde **15** as a light yellow oil* (211 mg, 76%): R*_f_* 0.47 (Et_2_O/hexane, 2:3); ^1^H NMR (CDCl_3_, 500 MHz) δ 9.63 (t, *J* = 2.1 Hz, 1H), 5.27 (t, *J* = 7.3 Hz, 1H), 5.10–5.08 (m, 1H), 3.14 (dd, *J* = 7.3, 1.9 Hz, 2H), 2.09–2.02 (m, 6H), 1.68 (s, 3H), 1.60 (s, 3H), 0.98 (t, *J* = 7.6 Hz, 3H); ^13^C NMR (CDCl_3_, 125 MHz) δ 200.3, 147.1, 131.8, 123.9, 112.2, 43.0, 36.4, 26.7, 25.7, 23.6, 17.7, 13.0; HRMS (ESI/Q-ToF) *m*/*z*: [M + H]^+^ Calcd for C_12_H_21_O 181.1587; Found 181.1587 (0.0 ppm). *Note: Aldehyde **15** was found to be particularly volatile and demanded careful observation during concentration of fractions following flash column chromatography.

### Methyl 2-[bis­(2,2,2-trifluoroethoxy)­phosphoryl]­propanoate (17)

Following a previously reported procedure,^38^ to a dry 100 mL round-bottom flask, containing methyl 2-[bis­(2,2,2-trifluoroethoxy)­phosphoryl]­acetate (1.67 mL, 7.86 mmol) dissolved in anhydrous THF (26 mL) under an Ar atmosphere at 0 °C, was added *t*-BuOK (1.058 g, 9.43 mmol). After stirring for 30 min, the resulting yellow solution was charged dropwise with MeI (2.45 mL, 39.3 mmol) over 15 min and then warmed to room temperature. Upon stirring for 24 h, the resulting being suspension was quenched with sat. aq. NH_4_Cl (15 mL) and repeatedly extracted with EtOAc (10 mL x 3). The combined organic layers were dried over anhydrous Na_2_SO_4_ and concentrated under reduced pressure to afford an orange oil that was further purified by gradient flash column chromatography (0–30% EtOAc in hexane) to afford title compound **17** as a colorless oil (1.148 g, 44%): R*_f_* 0.90 (EtOAc; visualization with alkaline KMnO_4_); ^1^H NMR (CDCl_3_, 400 MHz) δ 4.49–4.36 (m, 4H), 3.78 (d, ^5^ *J*_HP_ = 0.3 Hz, 3H), 3.20 (dq, ^2^ *J*_HP_ = 22.8, *J*_HH_ 7.4 Hz, 1H), 1.52 (dd, ^3^ *J*_HP_ = 19.2, *J*_HH_ = 7.3 Hz, 3H). Physical and spectral data agreed with those reported previously.^46^

### Methyl (2*Z*,5*E*)-6-ethyl-2,10-dimethylundeca-2,5,9-trienoate (14)

To a dry 25 mL pear-shaped round-bottom flask, containing dry 18-crown-6 ^47^ (799 mg, 2.95 mmol) under an Ar atmosphere, was added anhydrous THF (2.5 mL). Following the complete dissolution of 18-crown-6, methyl 2-[bis­(2,2,2-trifluoroethoxy)­phosphoryl]­propanoate (**17**, 196 mg, 0.59 mmol) was added and the resulting mixture cooled to – 78 °C. The solution was charged dropwise with KHMDS (1 M in THF, 0.59 mmol) and aged at – 78 °C for a further 10 min. To the resulting amber mixture was added dropwise a solution of aldehyde **15** (106 mg, 0.59 mmol) in anhydrous THF (2.5 mL). After stirring for 1.5 h (complete consumption of starting material **15** was confirmed by TLC [E_2_O/pentane, 1:19]), the reaction mass was charged with sat. aq. NH_4_Cl (5 mL) and then allowed to warm to room temperature. The resulting biphasic mixture was further diluted with H_2_O (5 mL), prior to being repeatedly extracted with Et_2_O (10 and 5 mL x 3). The combined organic layers were dried over anhydrous Na_2_SO_4_ and then carefully concentrated under reduced pressure to afford a residue that was purified by gradient flash column chromatography (0–5% Et_2_O in pentane) to afford title compound **14** as a colorless oil (124 mg, 84%): R*_f_* 0.53 (Et_2_O/pentane, 1:19); ^1^H NMR (CDCl_3_, 500 MHz) δ 5.91–5.87 (m, 1H, H3), 5.10 (t, *J* = 7.3 Hz, 2H, H5/H9), 3.75 (s, 3H, H16), 3.19 (t, *J* = 7.2 Hz, 2H, H4), 2.08–1.99 (m, 6H, H7/H8/H13), 1.90 (appd, *J* = 1.4 Hz, 3H, H15), 1.68 (s, 3H, H11), 1.60 (s, 3H, H12), 0.96 (t, *J* = 7.6 Hz, 3H, H14); ^13^C NMR (CDCl_3_, 125 MHz) δ 168.5 (C1), 142.6 (C6), 142.4 (C3), 131.4 (C10), 126.2 (C2), 124.3 (C9), 121.0 (C5), 51.3 (C16), 36.5 (C7), 28.3 (C4), 26.8 (C8), 25.7 (C11), 23.2 (C13), 20.7 (C15), 17.7 (C12), 13.2 (C14); HRMS (ESI/Q-ToF) *m*/*z*: [M + H]^+^ Calcd for C_16_H_27_O_2_^+^ 251.2006; Found 251.2006 (0.0 ppm).

### (2*Z*,5*E*)-6-Ethyl-2,10-dimethylundeca-2,5,9-trien-1-ol (19)

To a dry 100 mL round-bottom flask under an Ar atmosphere, containing a solution of methyl ester **14** (120 mg, 0.48 mmol) in anhydrous Et_2_O (5 mL) cooled to – 78 °C, was added dropwise DIBAL-H (1 M in THF, 1.1 mL, 1.1 mmol). The resulting mixture was allowed to warm to room temperature and stir for 2.5 h (complete consumption of starting material **14** was confirmed by TLC [Et_2_O/pentane, 1:19]). Upon cooling to 0 °C, the reaction mass was then charged with sat. aq. Na–K-tartrate (5 mL) and further stirred at room temperature until complete layer separation was observed. The aqueous layer was extracted with Et_2_O (10 mL x 3) and the combined organic layers were dried over anhydrous Na_2_SO_4_ and concentrated under reduced pressure to afford a residue that was further purified by gradient flash column chromatography (0–40% Et_2_O in pentane) to afford title compound **19** as a colorless oil (80 mg, 75%): R*_f_* 0.47 (Et_2_O/pentane, 2:3); ^1^H NMR (CDCl_3_, 500 MHz) δ 5.30 (t, *J* = 7.5 Hz, 1H), 5.11–5.08 (m, 1H), 5.04 (t, *J* = 7.2 Hz, 1H), 4.16 (s, 3H), 2.77 (t, *J* = 7.3 Hz, 2H), 2.07–2.03 (m, 4H), 2.01–1.98 (m, 2H), 1.80 (appd, *J* = 1.1 Hz, 3H), 1.68 (s, 3H), 1.60 (s, 3H), 1.38 (brs, 1H), 0.97 (t, *J* = 7.6 Hz, 3H); ^13^C NMR (CDCl_3_, 125 MHz) δ 141.5, 134.2, 131.4, 127.5, 124.3, 122.0, 61.7, 36.5, 26.8, 26.2, 25.7, 23.2, 21.3, 17.7, 13.1; HRMS (ESI/Q-ToF) *m*/*z*: [M + H]^+^ Calcd for C_15_H_27_O^+^ 223.2056; Found 223.2053 (− 1.3 ppm).

### (3*Z*,6*E*)-14-Methyl-α-farnesene (1b)

To a dry 100 mL round-bottom flask under an Ar atmosphere, containing allylic alcohol **19** (50 mg, 0.23 mmol) in anhydrous CH_2_Cl_2_ (5 mL), was added MnO_2_ (293 mg, 3.37 mmol). After stirring in darkness for 24 h at room temperature, the resulting black suspension was passed through a short plug of silica and eluted with Et_2_O. The filtrate was carefully concentrated under reduced pressure to afford a light yellow residue that was immediately purified by preparative thin-layer chromatography (Et_2_O/pentane, 1:40) to give α,β-unsaturated aldehyde intermediate **13** as a colorless oil which was then carried forward to the next reaction step without any further purification. In a separate dry 50 mL round-bottom flask under an Ar atmosphere, containing an ice-cold suspension of methyltriphenylphosphonium iodide (279 mg, 0.69 mmol) in anhydrous Et_2_O (5 mL), was added dropwise *n*-BuLi (2.5 M in hexane, 0.28 mL, 0.69 mmol). After stirring at 0 °C for 15 min, the resulting amber mixture was charged dropwise with a solution of α,β-unsaturated aldehyde 36 in anhydrous Et_2_O (3 mL). Following the complete addition of 36, the reaction mass was allowed to warm to room temperature and stir for a further 16 h. Upon cooling to 0 °C, the beige suspension was subsequently treated with sat. aq. NH_4_Cl (5 mL). The aqueous layer was further extracted with pentane (5 mL x 3) and the combined organic layers were then dried over anhydrous Na_2_SO_4_ and carefully concentrated under reduced pressure to afford a light amber residue that was further purified by preparative thin-layer chromatography (pentane) to give title compound **1b** as a colorless oil* (6 mg, 12% [over two steps from **19**]): R*_f_* 0.75 (pentane); ^1^H NMR (CDCl_3_, 500 MHz) δ 6.81 (ddd, *J* = 17.3, 10.8, 0.7 Hz, 1H, H2), 5.35 (t, *J* = 7.4 Hz, 1H,H4), 5.20 (d, *J* = 16.9 Hz, 1H, H1a), 5.11–5.06 (m, 3H, H1b/H6/H10), 2.88 (t, *J* = 7.4 Hz, 2H, H5), 2.10–1.98 (m, 6H, H8/H9/H14), 1.82 (d, *J* = 1.0 Hz, 3H, H16), 1.68 (s, 3H, H12), 1.60 (s, 3H, H13), 0.97 (t, *J* = 7.6 Hz, 3H, H15); ^13^C NMR (CDCl_3_, 125 MHz) δ 141.6 (C7), 133.7 (C2), 131.9 (C3), 131.4 (C11), 130.0 (C4), 124.4 (C10), 121.8 (C6), 113.5 (C1), 36.5 (C8), 26.9 (C9), 26.0 (C5), 25.7 (C12), 23.3 (C14), 19.8 (C16), 17.7 (C13), 13.2 (C15); HRMS (EI/Orbitrap) *m*/*z*: [M^+·^] Calcd for C_16_H_26_ 218.2029; Found 218.2031 (0.9 ppm). *Note: Due to the observed volatility of (3*Z*,6*E*)-14-methyl-α-farnesene (1b), NMR characterization was performed in the presence of residual Et_2_O and pentane.

## Supplementary Material



## Data Availability

NMR data (^1^H, ^13^C, ^1^H–^1^H COSY, ^1^H–^13^C HSQC, ^1^H–^13^C HMBC, and ^1^H–^1^H ROESY) for (3*Z*,6*E*)-14-Methyl-α-farnesene (**1b**) have been deposited in the Natural Products Magnetic Resonance Database (NP-MRD; www.np-mrd.org) and can be found at NP0351196 ((3Z,6E)-14-Methyl-alpha-farnesene).

## Notes

Comparative GC-EI-MS data for *O*. *biroi* larvae and pupae extracts; Detailed synthetic procedures, compound characterization, and spectroscopic data (PDF)
