# Supplementary material for: Identification and Stereoselective Total Synthesis of an Insect Homosesquiterpene from the Clonal Raider Ant Ooceraea biroi
Source: J Nat Prod. 2025 Aug 27;88(9):2107–16. doi: 10.1021/acs.jnatprod.5c00656 (PMC12481566; doi:10.1021/acs.jnatprod.5c00656)
Supplement: Supplementary file 1 [file np5c00656_si_001.pdf]

# Supporting Information

## Identification and stereoselective total synthesis of an insect homosesquiterpene from the Clonal raider ant *Ooceraea biroi*

Ryan M. Alam[a]*, Yoko Nakamura[a,b], Stefan Bartram[a], Nico Ueberschaar[c], Tim Zetzsche[d], Yuko Ulrich[d], Sarah E. O'Connor[a]*, and Tobias G. Köllner[a]*

[a]Department of Natural Product Biosynthesis, Max Planck Institute for Chemical Ecology, Hans-Knöll-Straße 8, D-07745, Jena, Germany.

[b]NMR/Biosynthesis Group, Max Planck Institute for Chemical Ecology, Hans-Knöll-Straße 8, D-07745, Jena, Germany.

[c]Lise Meitner Research Group Social Behavior, Max Planck Institute for Chemical Ecology, Hans-Knöll-Straße 8, D-07745, Jena, Germany.

[d]Institute for Inorganic and Analytical Chemistry, Mass Spectrometry Platform, Friedrich Schiller University Jena, Humboldtstraße 8, D-07743, Jena, Germany.

*Ryan M. Alam – Email: ralam@ice.mpg.de; Sarah E. O'Connor – Email: oconnor@ice.mpg.de;

Tobias G. Köllner – Email: koellner@ice.mpg.de

## Table of Contents

Comparison of extracted volatiles detected in *O. biroi* larvae, pupae, and workers......4
Synthesis of *rac*-4-methylhexan-3-one (**S1**)......5
Identification of 4-methylheptan-3-one (**S1**) and 4-methylheptan-3-ol (**S2**)......6
Identification of *n*-undecane......7
Synthesis of β-springene (**S3**)......8
(2*E*,6*E*,10*E*)-1-Methoxy-3,7,11,15-tetramethylhexadeca-2,6,10,14-tetraene (**S5**)......8
β-Springene (**S3**)......9
Identification of β-springene (**S3**)......10
Attempted synthesis of 14-methyl-α-farnesene stereoisomers **1a–d**[5]......11
Ethyl 5-methyl-2-propionylhex-4-enoate (**S6**)......11
7-Methyloct-6-en-3-one (**2**)......11
(4-Methylhexa-3,5-dien-1-yl)triphenylphosphonium iodide (**S8**)......12
6-Iodo-3-methylhexa-1,3-diene (**S11**)......12
(4-Methylhexa-3,5-dien-1-yl)triphenylphosphonium iodide (**S8**)......13
14-Methyl-α-farnesene (**1a–d**)......14
Non-stereoselective synthesis of 14-methyl-α-farnesene stereoisomers **1a–d**......15
Ethyl 3-ethyl-7-methylocta-2,6-dienoate (**6**)......15
3-Ethyl-7-methylocta-2,6-dien-1-ol (**7**)......16
4-Ethyl-8-methylnona-3,7-dienenitrile (**8**)......17
4-Ethyl-8-methylnona-3,7-dienoic acid (**5**)......17
5-[(4-Ethyl-8-methylnona-3,7-dien-1-yl)thio]-1-methyl-1*H*-tetrazole (**10**)......18
5-[(4-Ethyl-8-methylnona-3,7-dien-1-yl)sulfonyl]-1-methyl-1*H*-tetrazole (**11**)......19
14-Methyl-α-farnesene (**1a–d**)......20
Nominal EI-MS Spectra of 14-methyl-α-farnesene isomers **1a–d**......21
Synthesis of (*E*)-3-Ethyl-7-methylocta-2,6-dien-1-ol ((*E*)-**7**)......22
Ethyl 7-methyl-3-oxooct-6-enoate (**S14**)......22
Ethyl (*Z*)-7-methyl-3-{[(trifluoromethyl)sulfonyl]oxy}octa-2,6-dienoate (**S15**)......23
Ethyl (*E*)-3-ethyl-7-methylocta-2,6-dienoate ((*E*)-**6**)......23
(*E*)-3-Ethyl-7-methylocta-2,6-dien-1-ol ((*E*)-**7**)......24
NMR data......25
$^1H$ NMR (400 MHz, $CDCl_3$) **S1**......25
$^1H$ NMR (400 MHz, $CDCl_3$) **S5**......25
$^1H$ NMR (400 MHz, $CDCl_3$) **S3**......26
$^1H$ NMR (400 MHz, $CDCl_3$) **1a–d**......26

$^{1}H$ NMR (400 MHz, $CDCl_3$) **S14** ........ 27
$^{1}H$ NMR (400 MHz, $CDCl_3$) **S15** ........ 27
$^{1}H$ NMR (400 MHz, $CDCl_3$) (*E*)-**6** ........ 28
$^{1}H$ NMR (400 MHz, $CDCl_3$) (*E*)-**7** ........ 28
$^{1}H$ NMR (400 MHz, $CDCl_3$) (*E*)-**8** ........ 29
$^{13}C$ NMR (100 MHz, $CDCl_3$) (*E*)-**8** ........ 29
$^{1}H$ NMR (400 MHz, $CDCl_3$) **18** ........ 30
$^{13}C$ NMR (100 MHz, $CDCl_3$) **18** ........ 30
$^{1}H$ NMR (500 MHz, $CDCl_3$) (*E*)-**5** ........ 31
$^{13}C$ NMR (125 MHz, $CDCl_3$) (*E*)-**5** ........ 31
$^{1}H$ NMR (500 MHz, $CDCl_3$) **15** ........ 32
$^{13}C$ NMR (125 MHz, $CDCl_3$) **15** ........ 32
$^{1}H$ NMR (400 MHz, $CDCl_3$) **17** ........ 33
$^{1}H$ NMR (500 MHz, $CDCl_3$) **14** ........ 33
$^{13}C$ NMR (125 MHz, $CDCl_3$) **14** ........ 34
$^{1}H$-$^{1}H$ COSY NMR (500 MHz, $CDCl_3$) **14** ........ 34
$^{1}H$-$^{13}C$ HSQC (500 MHz, $CDCl_3$) **14** ........ 35
$^{1}H$-$^{13}C$ HMBC (500 MHz, $CDCl_3$) **14** ........ 35
$^{1}H$-$^{1}H$ ROESY (500 MHz, $CDCl_3$) **14** ........ 36
$^{1}H$ NMR (500 MHz, $CDCl_3$) **19** ........ 36
$^{13}C$ NMR (125 MHz, $CDCl_3$) **19** ........ 37
$^{1}H$ NMR (500 MHz, $CDCl_3$) **1b** ........ 37
$^{13}C$ NMR (125 MHz, $CDCl_3$) **1b** ........ 38
$^{1}H$-$^{13}C$ HSQC (500 MHz, $CDCl_3$) **1b** ........ 38
$^{1}H$-$^{13}C$ HMBC (500 MHz, $CDCl_3$) **1b** ........ 39
$^{1}H$-$^{1}H$ ROESY (500 MHz, $CDCl_3$) **1b** ........ 39
References ........ 40

## Comparison of extracted volatiles detected in *O. biroi* larvae, pupae, and workers

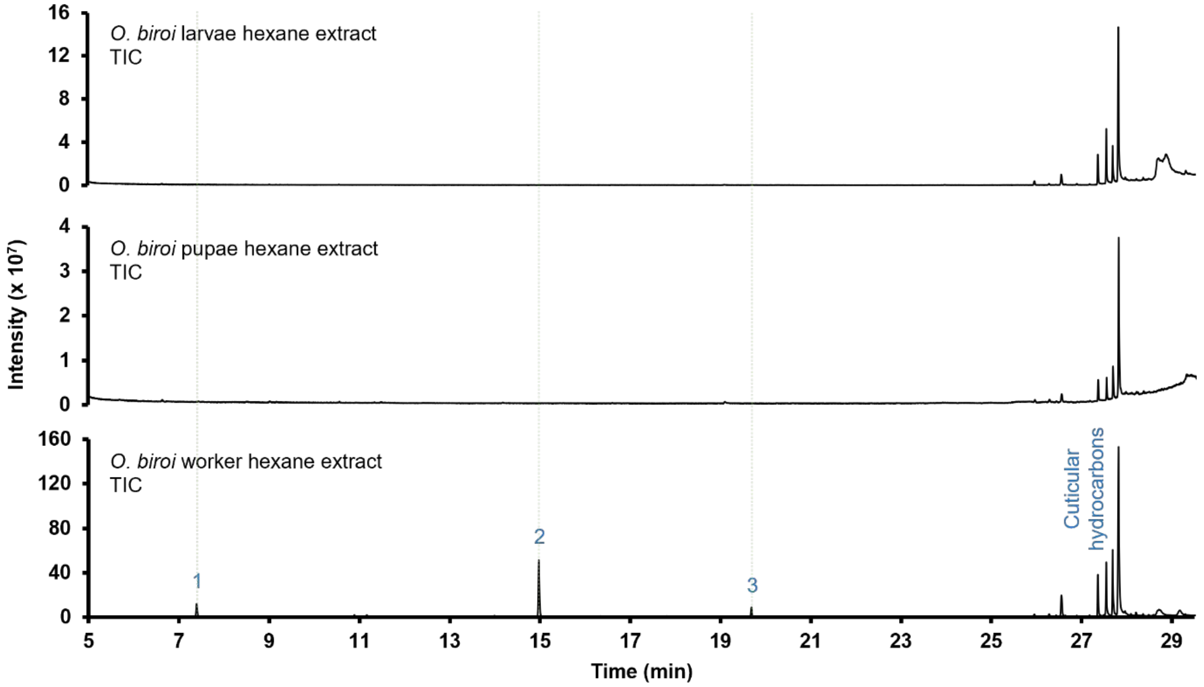


**Figure S1.** Comparison of GC-EI-MS data* obtained for *O. biroi* larvae (top), pupae (middle), and workers (bottom) shows that only workers contain *n*-undecane (peak 1), (3*Z*,6*E*)-14-methyl-α-farnesene (**1a**; peak 2), and β-springene (**S3**; peak 3).

*Data was acquired using a TRACE™ 1310 GC ultra-gas chromatograph equipped with a split–splitless injector and ISQ quadrupole mass spectrometer (ThermoFisher Scientific, Germering, Germany). Chromatographic separation was carried out using helium as the carrier gas applied at a constant flow rate of 1.1 mL/min with an injection volume of 1 µL in split mode and were all carried out using a PAL autosampler (CTC Analytics AG, Zwingen, Switzerland). The injector and transfer line temperatures were 230 °C and 280 °C, respectively. For chromatographic separation, an initial column oven temperature of 50 °C was held for 2 min and then increased by 8 °C/min to 250 °C, prior to being heated to 300 °C (100 °C/min ramp) and held at 300 °C for 2 min, using a Zebron ZB-5™ column (5% phenyl, 95% dimethylpolysiloxane; 30 m length, 0.25 mm inner diameter, 0.25 µm film thickness, 10 m pre-column, Phenomenex, Aschaffenburg, Germany). Solvent delay was set to 5 min. The ion source temperature was 250 °C. MS data acquisition was carried out in scan mode (mass range, 35–450 *m/z*). The ionization energy was 70 eV.

## Synthesis of *rac*-4-methylhexan-3-one (S1)

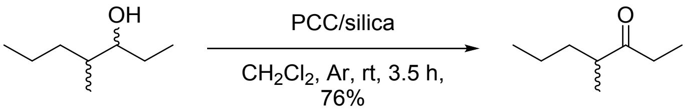


**S2** **S1**

To a dry 8 mL vial was added a freshly ground mixture of pyridinium chlorochromate (259 mg, 1.2 mmol) and silica (259 mg). The bright orange solid was suspended in anhydrous $CH_2Cl_2$ (4 mL) and charged dropwise with 4-methylheptan-3-ol (**S2**, 0.14 mL, 1 mmol). Upon stirring at room temperature for 3.5 h, the black reaction mass was passed through a short plug of silica and further eluted with $CH_2Cl_2$ (4 mL x 3) to give a colorless eluate that was then carefully concentrated under reduced pressure to afford title compound **S1** as a colorless oil (97 mg, 76%): $^1$H NMR ($CDCl_3$, 400 MHz) $\delta$ 2.54 (qt, *J* = 6.7, 6.7 Hz, 1H), 2.50–2.39 (m, 2H), 1.68–1.58 (m, 1H), 1.35–1.19 (m, 3H), 1.06 (overlapping d, *J* = 6.9 Hz, 3H), 1.04 (overlapping t, *J* = 7.3 Hz, 3H), 0.89 (t, *J* = 7.2 Hz, 3H). Spectral data agreed with those reported previously.[1]

## Identification of 4-methylheptan-3-one (S1) and 4-methylheptan-3-ol (S2)

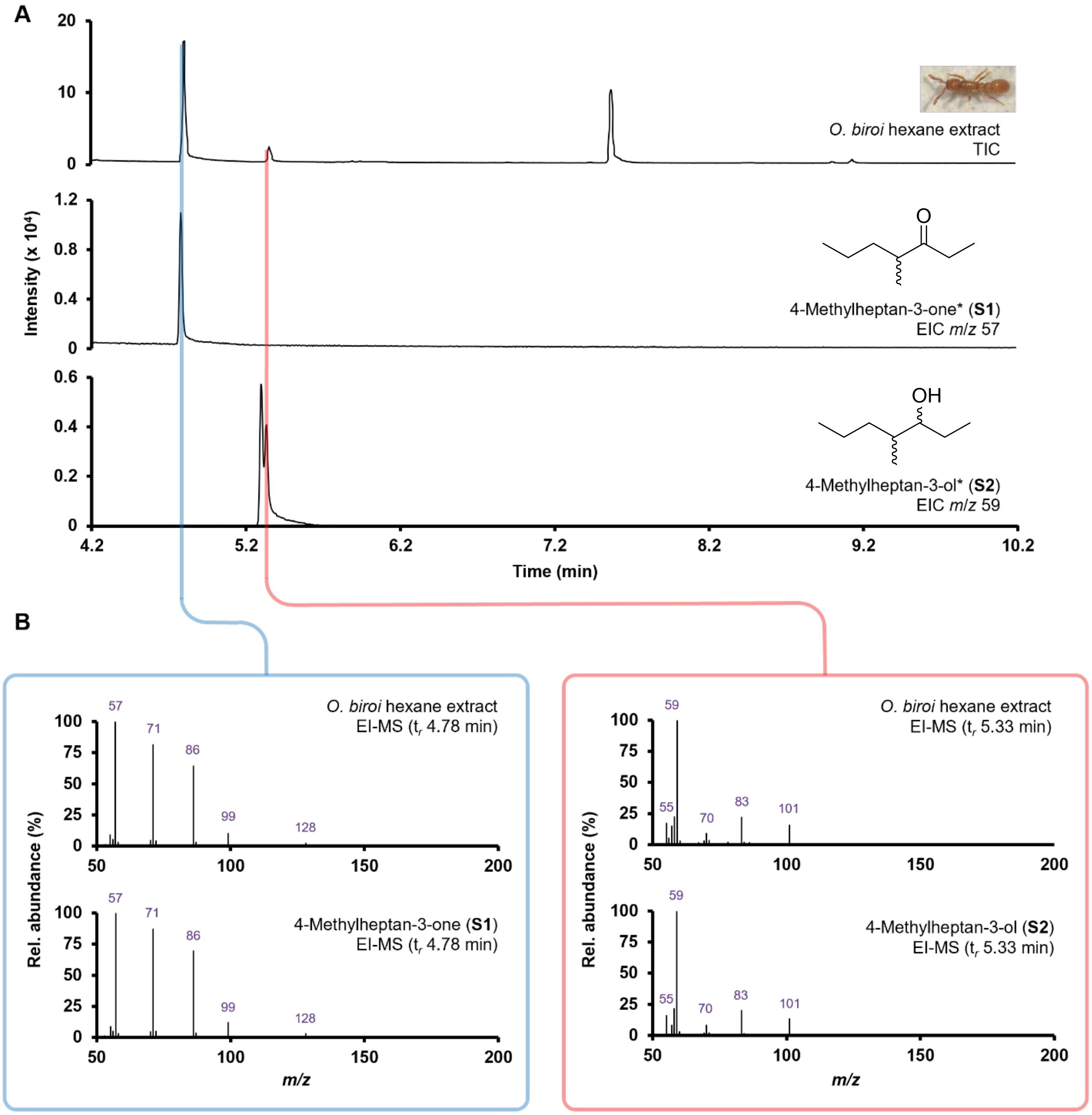


**Figure S2.** Comparison of GC-EI-MS chromatograms (**A**) and mass spectra (**B**) obtained from an *O. biroi* hexane extract (top) and synthetic *rac*-4-methylheptan-3-one (**S1**, middle) and 4-methylheptan-3-ol (**S2**, bottom) dissolved in hexane. Column: Zebron ZB-5$^{TM}$. *Mixture of stereoisomers.

## Identification of *n*-undecane

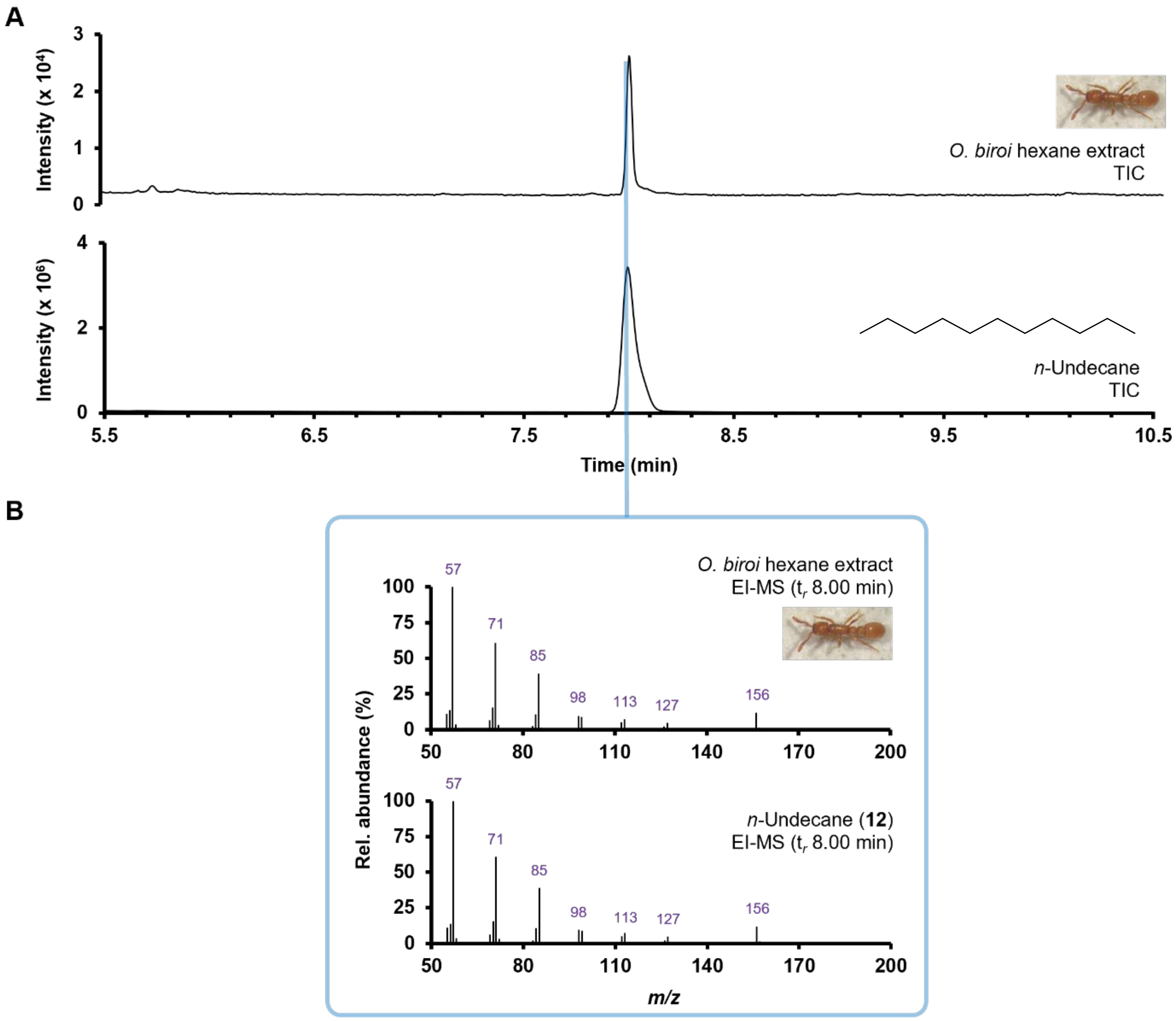


**Figure S3**. Comparison of GC-EI-MS chromatograms (**A**) and mass spectra (**B**) obtained from an *O. biroi* hexane extract (top) and an *n*-undecane standard (bottom) dissolved in $CH_2Cl_2$. Column: Zebron ZB-5$^{TM}$.

## Synthesis of β-springene (S3)

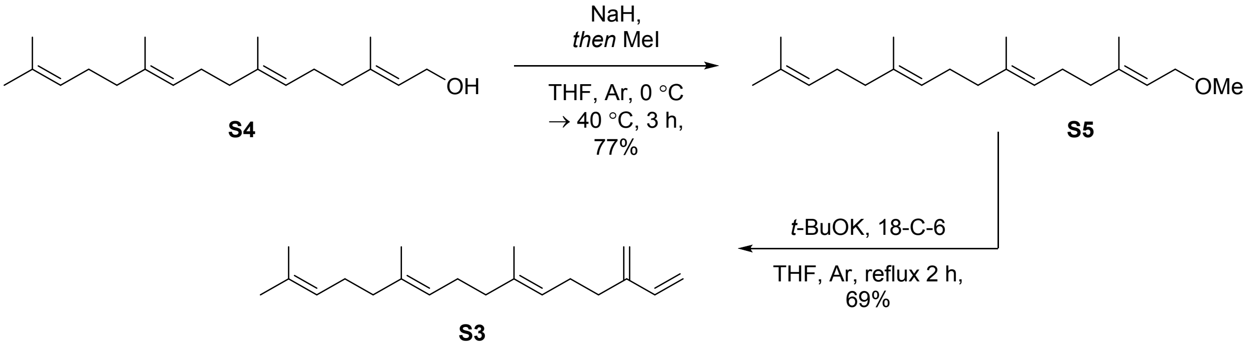


**Scheme S1** β-Springene (**S3**) was prepared over two steps from commercially available geranylgeraniol (**S4**), following Okuda's route.[2]

### (2*E*,6*E*,10*E*)-1-Methoxy-3,7,11,15-tetramethylhexadeca-2,6,10,14-tetraene (S5)

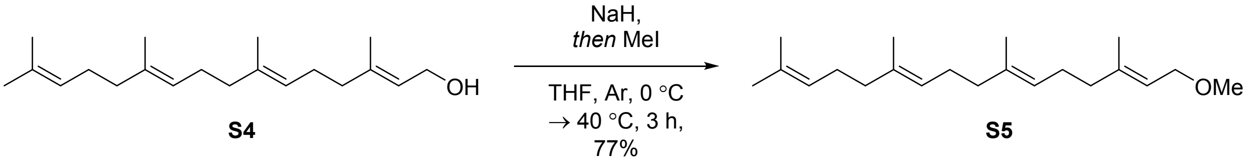


To a dry 10 mL round bottom flask under an Ar atmosphere, containing NaH (60% [w/w] dispersion in mineral oil, 76 mg, 1.89 mmol) in anhydrous $Et_2O$ (2.2 mL), was added geranylgeraniol (**S4**, 0.56 mL, 1.72 mmol). After stirring for 30 min at 0 °C, the mixture was charged dropwise with methyl iodide (1.39 mL, 2.24 mmol) and then heated to 40 °C (oil bath) for 3 h. The reaction mass was cooled to room temperature and diluted dropwise with $H_2O$ (15 mL). Following layer separation, the resulting aqueous phase was then extracted with $Et_2O$ (10 mL x 3) and the combined organic layers were washed with brine (10 mL), dried over anhydrous $Na_2SO_4$, and concentrated *in vacuo* to give an amber residue. Purification of the residue by gradient flash column chromatography (0–10% $Et_2O$ in pentane) afforded methyl ether **S5** as a yellow oil (405 mg, 77%): $R_f$ 0.32 ($Et_2O$/pentane, 1:9); $^1H$ NMR ($CDCl_3$, 400 MHz) $\delta$ 5.35 (td, $J$ = 6.8, 1.2 Hz, 1H), 5.13–5.08 (m, 3H), 3.93 (d, $J$ = 6.8 Hz, 2H), 3.32 (s, 3H), 2.15–1.95 (m,12H), 1.68 (s, 6H), 1.60 (s, 9H). Spectral data agreed with those reported previously.[3]

**β-Springene (S3)**

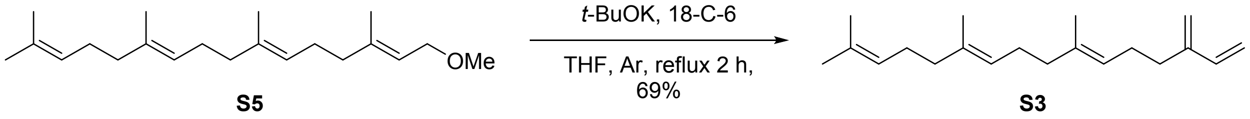


To a dry 50 mL round bottom flask under an Ar atmosphere was added methyl ether **S5** (280 mg, 0.92 mmol) and anhydrous THF (9 mL). The solution was sequentially charged with *t*-BuOK (1.032 g, 9.20 mmol) and 18-crown-6 (486 mg, 1.84 mmol) and heated to reflux (oil bath, 75 °C). After stirring at reflux for 2 h, the resulting dark blue reaction mixture was cooled and diluted with $H_2O$ (25 mL). The aqueous mixture was repeatedly extracted with $Et_2O$ (10 mL x 3) and the combined organic layers were then washed with brine (10 mL). Further drying of the organic phase over anhydrous $MgSO_4$ and concentration under reduced pressure afforded a dark brown residue that was purified by gradient flash column chromatography (pentane) to yield title compound **S3** as an amber oil (172 mg, 69%): $R_f$ 0.72 (pentane); $^1$H NMR ($CDCl_3$, 400 MHz) $\delta$ 6.38 (dd, $J$ = 17.6, 10.8 Hz, 1H), 5.25 (d, $J$ = 17.6 Hz, 1H), 5.18–5.15 (m, 1H), 5.13–5.07 (m, 2H), 5.06 (d, $J$ = 10.8 Hz, 1H), 5.01 (s, 1H), 5.00 (s, 1H), 2.27–2.16 (m, 4H), 2.12–2.04 (m, 4H), 2.02–1.96 (m, 4H), 1.68 (s, 3H), 1.60 (s, 9H). Spectral data agreed with those reported previously.[4]

## Identification of β-springene (S3)

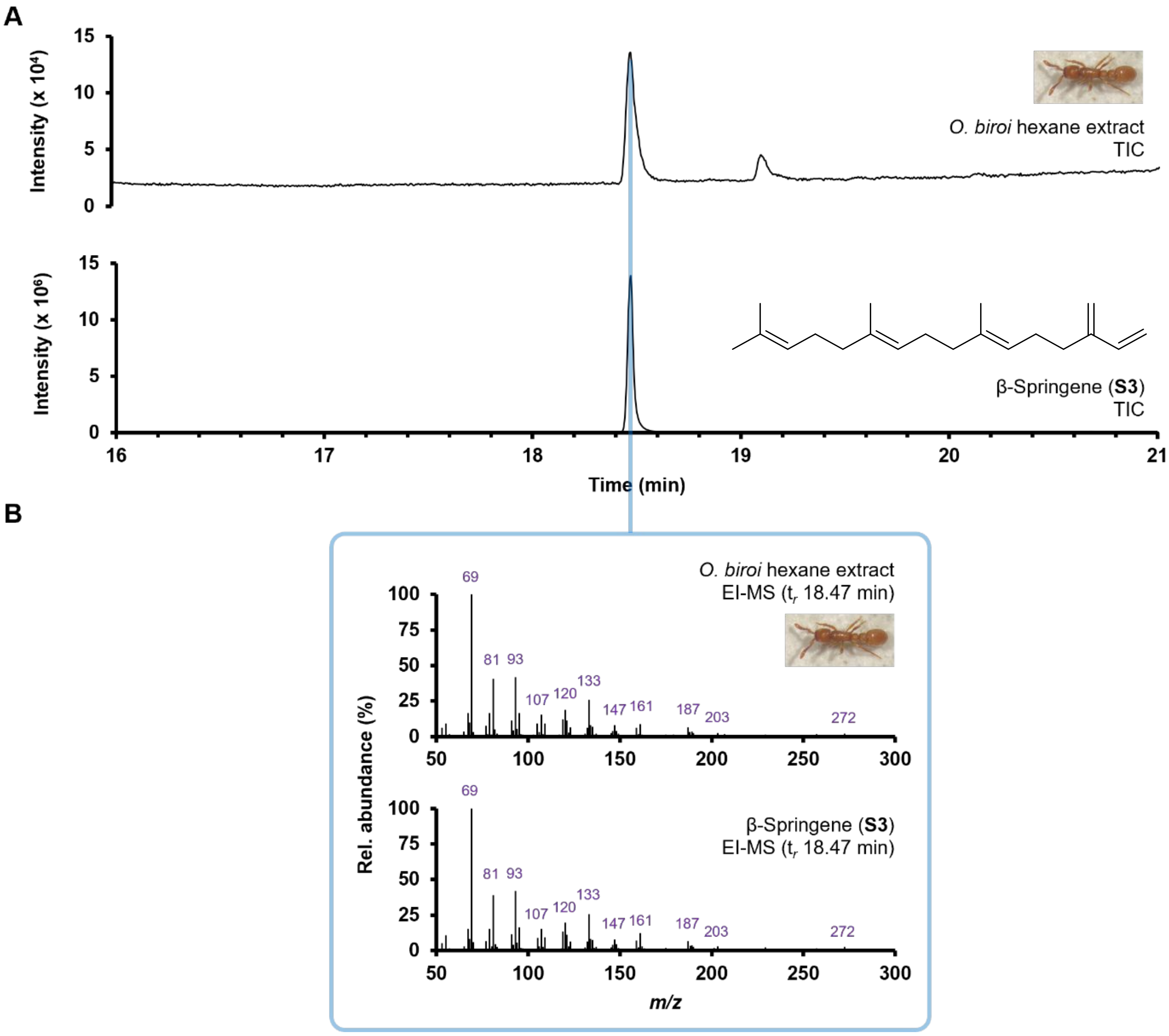


**Figure S4.** Comparison of GC-EI-MS chromatograms (**A**) and mass spectra (**B**) obtained from an *O. biroi* hexane extract (top) and a β-springene (**S3**, bottom) dissolved in $CH_2Cl_2$ solution standard. Column: Zebron ZB-5$^{TM}$.

## Attempted synthesis of 14-methyl-α-farnesene stereoisomers 1a–d[5]

### Ethyl 5-methyl-2-propionylhex-4-enoate (S6)

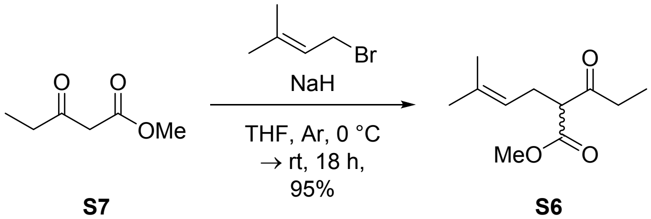


Following a previously reported method,[6] briefly a dry two-neck 250 mL round bottom flask fitted with a dropping funnel under an Ar atmosphere was added anhydrous THF (34 mL) and NaH (60% [w/w] dispersion in mineral oil, 1.477 g, 36.91 mmol). The solution was cooled to 0 °C and charged dropwise with a solution of β-keto ester **S7** (4.20 mL, 33.55 mmol). After stirring at 0 °C for 30 min, to the resulting suspension was added 3,3-dimethylallyl bromide (3.85 mL, 33.55 mmol) in anhydrous THF (17 mL) and subsequently warmed to room temperature. After stirring at room temperature for 18 h, the reaction mass was cooled to 0 °C and slowly quenched with $H_2O$ (10 mL) and then partitioned between a mixture of $H_2O$ (90 mL) and $CH_2Cl_2$ (40 mL). The aqueous layer was further extracted with $CH_2Cl_2$ (40 mL x 3). The combined organic layers were dried over anhydrous $Na_2SO_4$ and carefully concentrated under reduced pressure to give a bright yellow residue that was purified by gradient flash column chromatography (0–10% $Et_2O$ in hexane) to afford title compound **S6** as a colorless oil (6.319 g, 95%):$^1$H NMR ($CDCl_3$, 400 MHz) *δ* 5.03–4.98 (m, 1H), 3.71 (s, 3H), 3.47 (t, *J* = 7.5 Hz, 1H), 2.63–2.43 (m, 4H), 1.67 (d, *J* = 0.8 Hz, 3H), 1.62 (s, 3H), 1.05 (t, *J* = 7.2 Hz, 3H).

### 7-Methyloct-6-en-3-one (2)

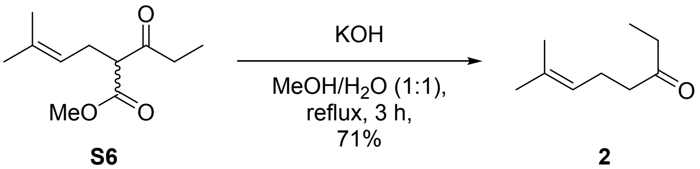


To a 100 mL round bottom flask containing β-keto ester **S6** (6.280 g, 31.68 mmol) was added MeOH (17.5 mL) and $H_2O$ (17.5 mL). The solution was then charged with KOH (2.919 g, 52.02 mmol) and heated to reflux. After stirring for 3 h, the reaction mass was cooled to room temperature, diluted with $H_2O$ (35 mL), and extracted with $Et_2O$ (25 mL x 3). The combined organic layers were then subsequently washed with $H_2O$ (25 mL) and brine (25 mL) and dried over

anhydrous $MgSO_4$. Careful concentration under reduced pressure afforded a colorless oil that was passed through a short plug of silica (0–10% $Et_2O$ in hexane). The resulting eluate was subsequently concentrated *in vacuo* to afford title compound **2** as a colorless oil (3.154 g, 71%): $^1H$ NMR ($CDCl_3$, 400 MHz) $\delta$ 5.06 (apptsept, $J$ = 7.1, 1.4 Hz, 1H), 2.41 (q, $J$ = 7.4 Hz, 4H), 2.25 (q, $J$ = 7.1 Hz, 2H), 1.67 (d, $J$ = 1.1 Hz, 3H), 1.61 (s, 3H), 1.05 (t, $J$ = 7.4 Hz, 3H). Spectral data agreed with those reported previously.[5]

### (4-Methylhexa-3,5-dien-1-yl)triphenylphosphonium iodide (S8)

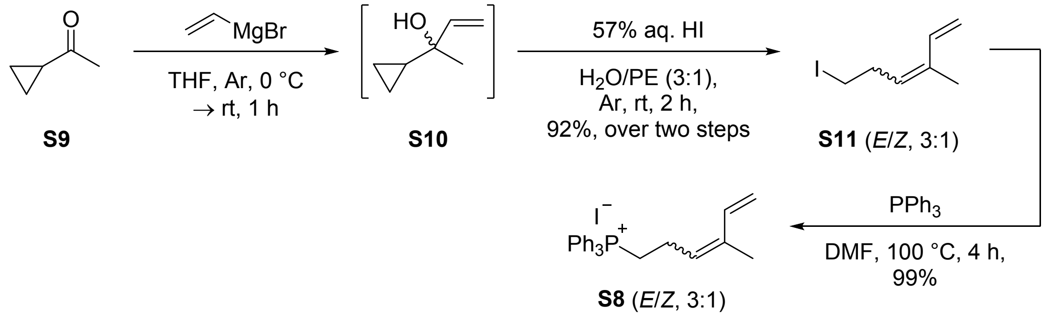


**Scheme S2.** Phosphonium iodide (**S8**) was prepared over four steps from commercially available cyclopropyl methyl ketone **S9**.

### 6-Iodo-3-methylhexa-1,3-diene (S11)

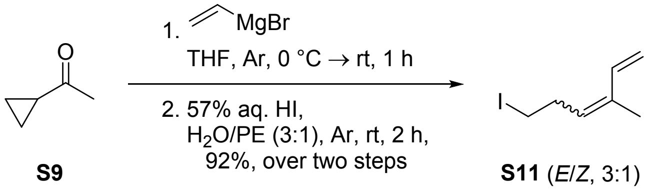


To a dry 250 mL round bottom flask under an Ar atmosphere was added 1 M vinylmagnesium bromide in THF (59.4 mL, 59.4 mmol). The orange solution was cooled to 0 °C and slowly charged dropwise with a solution of cyclopropyl methyl ketone (**S9**, 2.95 mL, 29.73 mmol) in anhydrous THF (10 mL) and then allowed to warm to room temperature. After stirring for 1 h, the reaction mass was cooled to 0°C and cautiously treated dropwise with $H_2O$ (5 mL) and further diluted with $H_2O$ (45 mL) and $CH_2Cl_2$ (25 mL) and separated. The aqueous layer was extracted with $CH_2Cl_2$ (25 mL x 3). The combined organic layers were dried over anhydrous $Na_2SO_4$ and carefully concentrated under reduced pressure to afford alcohol **S10** as a colorless oil that was then charged with a mixture of petroleum ether (b.p. 40–60 °C, 3.75 mL) and $H_2O$ (11.25 mL). Following the slow addition of 57% (w/w) aq. hydroiodic acid (13.74 mL, 104.06 mmol) to the

aforementioned mixture *via* a dropping funnel whilst vigorously stirring, the reaction mass was allowed to stir at room temperature for a further 2 h. To the resulting brown suspension was then added pentane (10 mL). Following layer separation, the aqueous phase was further extracted with pentane (10 mL x 3). The combined organic layers were washed with sat. aq. $Na_2S_2O_3$ (10 mL) and brine (10 mL), prior to being dried over anhydrous $MgSO_4$ and carefully concentrated *in vacuo* to afford a deep amber oil that was subsequently purified by vacuum distillation from silver foil to afford title compound **S11** (mixture of isomers [ratio *E*:*Z* = 3:1]) as a pale amber oil (6.070 g, 92% [over two steps from ketone **S9**]): b.p. 65–68 °C/5 mbar; $^1$H NMR ($CDCl_3$, 400 MHz) mixture of isomers* $\delta$ 6.69 (ddd, *J* = 17.2, 10.8, 0.8 Hz, 0.25H), 6.37 (dd, *J* = 17.4, 10.7 Hz, 0.75H), 5.43 (t, *J* = 7.1 Hz, 0.75H), 5.33 (t, *J* = 7.4 Hz, 0.25H), 5.30–5.02 (m, 2H), 3.15 (overlapping t, *J* = 7.3 Hz, 1.5H), 3.14 (overlapping t, *J* = 7.2 Hz, 0.5H), 2.78–2.72 (m, 2H), 1.83 (m, 0.75H), 1.75 (m, 2.25H). Spectral data for (*Z*)-**S11** agreed with those reported previously [5]

*Note: Both (*E*)-**S11** and (*Z*)-**S11** stereoisomers are described.

**(4-Methylhexa-3,5-dien-1-yl)triphenylphosphonium iodide (S8)**

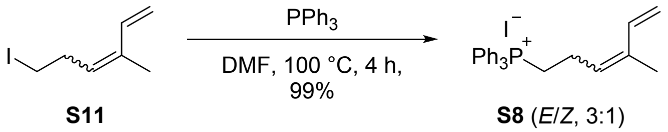


To a 50 mL round bottom flask containing alkyl iodide **S9** (6.007 g, 27.05 mmol) dissolved in DMF (10.8 mL) was added triphenylphosphine (7.095 g, 27.05 mmol). The resulting solution was heated to 100 °C and allowed to stir for 4 h. The reaction mass was then concentrated under reduced pressure to afford a viscous residue that was further purified by gradient flash column chromatography (0–20% MeOH in $CH_2Cl_2$) to afford phosphonium salt **S10** (mixture of isomers [ratio *E*:*Z* = 3:1]) as a viscous pale yellow oil which solidified on standing at room temperature (13.009 g, 99%): $^1$H NMR ($CDCl_3$, 400 MHz) mixture of isomers* $\delta$ 7.86–7.78 (m, 9H), 7.73–7.68 (m, 6H), 6.31 (dd, *J* = 17.2, 10.8 Hz, 0.25H), 6.19 (dd, *J* = 17.4, 10.8 Hz, 0.75H), 5.54 (overlapping t, *J* = 7.3 Hz, 0.75H), 5.50 (overlapping t, *J* = 7.3 Hz, 0.25H), 5.20–4.91 (m, 2H), 3.84–3.76 (m, 2H), 2.65–2.54 (m, 2H), 1.67 (s, 0.75H), 1.52 (s, 2.25H). Spectral data for (*E*)-**S10**[7] and (*Z*)-**S10**[5] agreed with those reported previously.

*Note: Both (*E*)-**S8** and (*Z*)-**S8** stereoisomers are described.

## 14-Methyl-α-farnesene (1a–d)

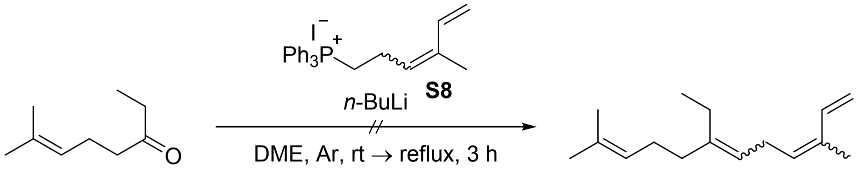


2 1a–d

To a dry 5 mL round bottom flask under Ar was added phosphonium salt **S8** (100 mg, 0.21 mmol) and anhydrous DME (1 mL). The mixture was treated dropwise with 2.5 M *n*-BuLi in hexane (0.08 mL, 0.21 mmol) and allowed to stir for 15 min at room temperature. The resulting deep red solution was then charged with ketone **2** (34 mg, 0.24 mmol), and heated to reflux (oil bath, 95 °C). After stirring at reflux for 3 h, the reaction mass was cooled to 0 °C and diluted with $H_2O$ (2 mL). The aqueous mixture was extracted with pentane (0.5 mL x 5) and the combined organic layers dried over anhydrous $Na_2SO_4$. Careful concentration of the combined organic phases under a gentle stream of Ar gave a deep amber residue. Analysis of the crude residue using $^1$H NMR and GC-EI-MS gave no detectable evidence for the formation of title compounds **1a–d**.

## Non-stereoselective synthesis of 14-methyl-α-farnesene stereoisomers 1a–d

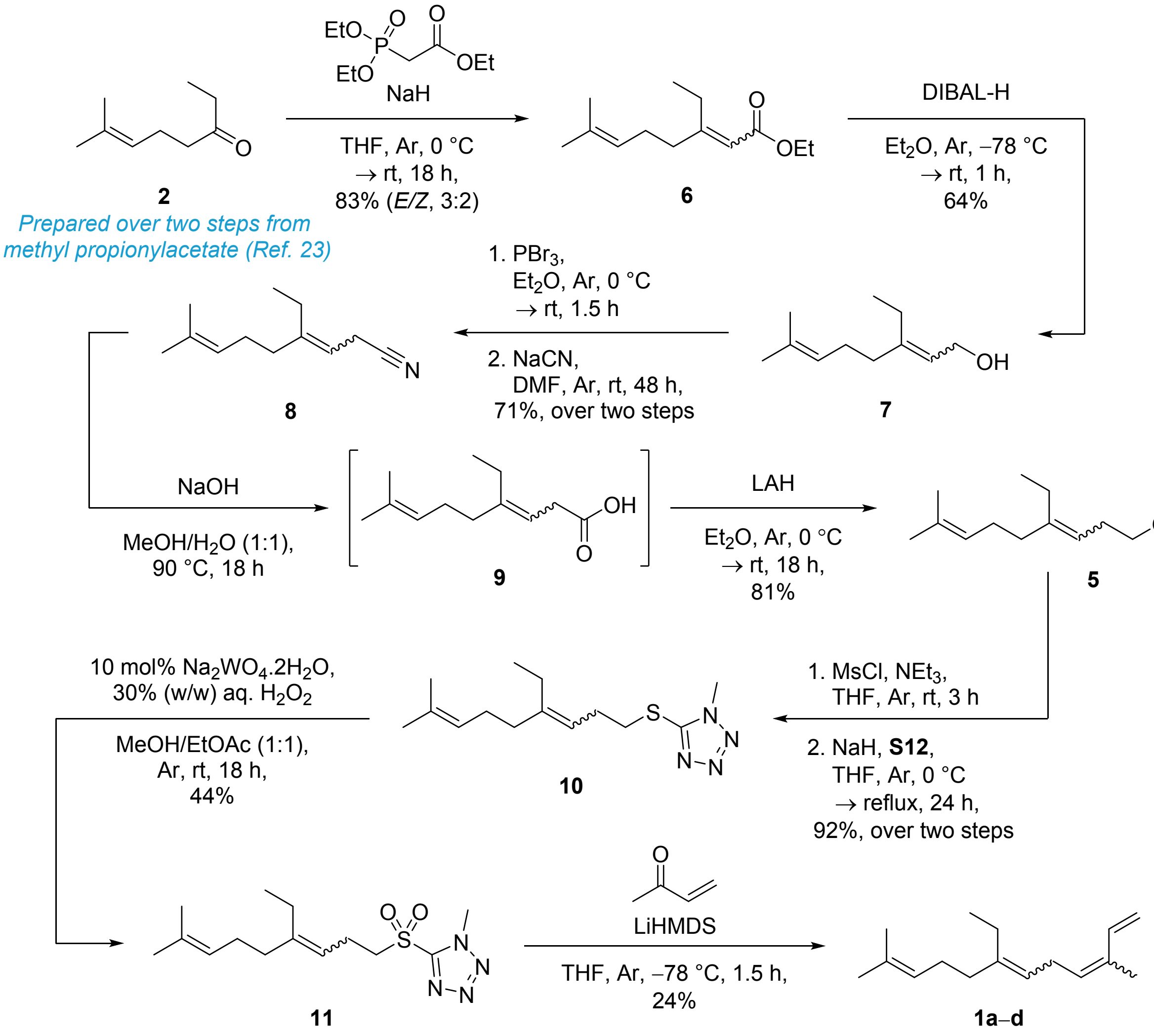


**Scheme S3.** Non-stereoselective synthesis of α-homofarnesene isomers **1a**–**d**.

### Ethyl 3-ethyl-7-methylocta-2,6-dienoate (6)

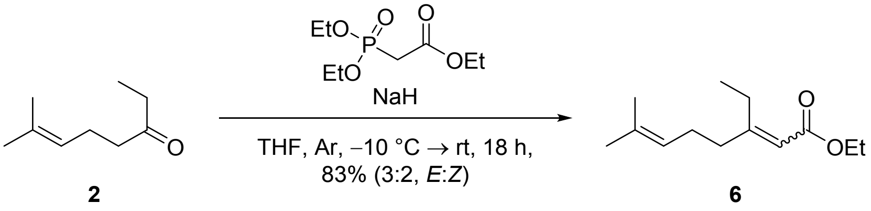


To a dry 100 mL round bottom flask under an Ar atmosphere, containing NaH (60% [w/w] in mineral oil, 1.712 g, 42.79 mmol) in anhydrous THF (15 mL) at −10 °C, was added dropwise triethyl phosphonoacetate (8.49 mL, 42.79 mmol). After warming to room temperature and stirring

for a further 30 min, the reaction mass was slowly charged with ketone **2** (2 g, 14.26 mmol) and then allowed to stir for 18 h. The resulting mixture was poured onto ice-cold $H_2O$ (100 mL) and extracted with $CH_2Cl_2$ (30 mL x 4). The combined organic layers were washed with $H_2O$ (20 mL) and brine (20 mL), dried over anhydrous $Na_2SO_4$, and concentrated *in vacuo* to give a residue that was purified by gradient flash column chromatography (0–5 % $Et_2O$ in hexane) to give title compound **6** as a colorless oil (2.498 g, 83%): $^1$H NMR ($CDCl_3$, 400 MHz) mixture of isomers* $\delta$ 5.62 (s, 0.4H), 5.61 (s, 0.6H), 5.18–5.13 (m, 0.4H), 5.11–5.07 (m, 0.6H), 4.14 (overlapping q, $J$ = 7.1 Hz, 0.8H), 4.14 (overlapping q, $J$ = 7.1 Hz, 1.2H), 2.64–2.59 (m, 2H), 2.22–2.11 (m, 4H), 1.69–1.68 (m, 3H), 1.61 (m, 3H), 1.28 (t, $J$ = 7.1 Hz, 3H), 1.07 (t, $J$ = 7.5 Hz, 1.8H), 1.06 (t, $J$ = 7.4 Hz, 1.2H). Physical and spectral data for both (*E*)-**6**[8] and (*Z*)-**6**[9] agreed with those reported previously.

*Note: Both (*E*)-**6** and (*Z*)-**6** stereoisomers are described.

**3-Ethyl-7-methylocta-2,6-dien-1-ol (7)**

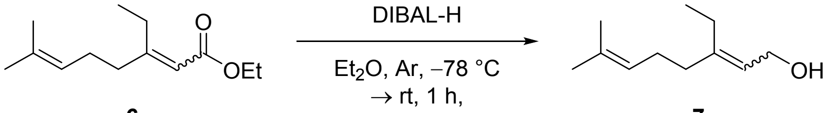


6 7

To a dry 100 mL round bottom flask under an Ar atmosphere, containing ethyl ester **6** (2 g, 9.51 mmol) in anhydrous $Et_2O$ (19 mL) cooled to –78 °C, was added dropwise 1 M DIBAL-H in THF (28.53 mL, 28.53 mmol). Following the complete addition of ethyl ester **6**, the reaction mass was allowed to warm to room temperature and stir for a further 1.5 h and then cooled to 0 °C. The resulting mixture was treated dropwise with sat. aq. Na-K-tartrate (30 mL) and stirred at room temperature for a further 1 h. The biphasic mixture was separated and the resulting aqueous layer repeatedly extracted with $Et_2O$ (15 mL x 3). The combined organic phases were dried over anhydrous $MgSO_4$ and concentrated under reduced pressure to afford a colorless residue that was further purified by gradient flash column chromatography (0–50% $Et_2O$ in pentane) to yield title compound **7** as a colorless oil (1.024 g, 64%): $^1$H NMR ($CDCl_3$, 400 MHz) mixture of isomers* $\delta$ 5.41 (t, $J$ = 7.1 Hz, 0.4H), 5.36 (t, $J$ = 7.0 Hz, 0.6H), 5.10 (t, $J$ = 6.4 Hz, 1H), 4.15 (d, $J$ = 7.0 Hz, 1.2H), 4.12 (d, $J$ = 7.1 Hz, 0.8H), 2.12–2.05 (m, 6H), 1.68 (s, 3H), 1.60 (s, 1.8H), 1.59 (s, 1.2H), 1.51 (brs, 1H), 1.02 (t, $J$ = 7.4 Hz, 1.2H), 0.98 (t, $J$ = 7.5 Hz, 1.8H).

*Note: Both (*E*)-**7** and (*Z*)-**7** stereoisomers are described.

**4-Ethyl-8-methylnona-3,7-dienenitrile (8)**

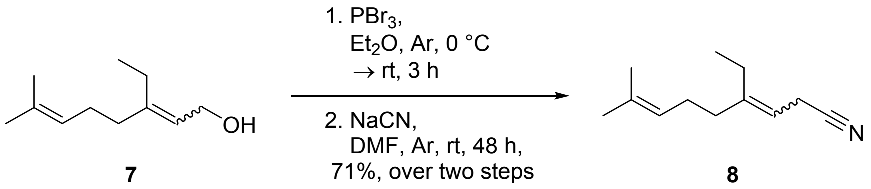


To a dry 50 mL round bottom flask under an Ar atmosphere, containing a cooled solution (0 °C) of allylic alcohol **7** (1 g, 5.94 mmol) in anhydrous $Et_2O$ (12 mL), was added dropwise $PBr_3$ (0.19 mL, 1.98 mmol). Upon warming to room temperature and stirring for a further 3 h, the reaction mass was charged with brine (10 mL) and repeatedly extracted with $Et_2O$ (5 mL x 3). The combined organic extracts were washed with brine (10 mL) and dried over anhydrous $Na_2SO_4$. Careful concentration of the crude mixture afforded an amber oil that was immediately carried forward to the next reaction step without any further purification. In a separate dry 25 mL round bottom flask under an Ar atmosphere, containing a vigorously stirring suspension of NaCN (320 mg, 6.53 mmol) in anhydrous DMF (5 mL), was added a solution of crude allyl bromide in DMF (5 mL). After stirring for two days at room temperature, the reaction mass was partitioned between $Et_2O$ (25 mL) and brine (40 mL). The aqueous layer was further extracted with $Et_2O$ (20 mL x 3). The combined organic layers were washed with brine (20 mL x 3), dried over anhydrous $Na_2SO_4$, and carefully concentrated under reduced pressure to afford a residue that was further purified by gradient flash column chromatography (0–5% $Et_2O$ in pentane) to afford title compound **8** as a colorless oil (748 mg, 71% [over two steps from **7**]): $^1$H NMR ($CDCl_3$, 400 MHz) mixture of isomers* $\delta$ 5.17–5.06 (m, 2H), 3.06 (d, $J$ = 7.1 Hz, 2H), 2.08–2.03 (m, 6H), 1.69 (s, 3H), 1.60 (s, 3H), 1.04–0.99 (m, 3H).

*Note: Both (*E*)-**8** and (*Z*)-**8** stereoisomers are described.

**4-Ethyl-8-methylnona-3,7-dienoic acid (5)**

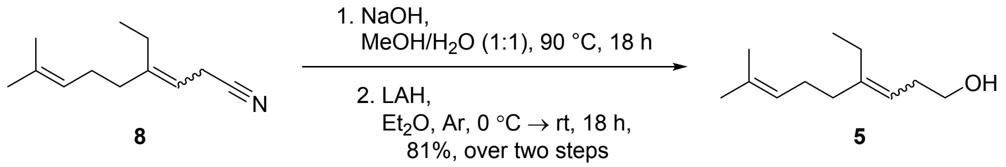


To a 50 mL round bottom flask, containing a solution of nitrile **8** (410 mg, 2.31 mmol) dissolved in MeOH/$H_2O$ (1:1, 4.6 mL), was added freshly powdered NaOH (370 mg, 9.25 mmol). The resulting mixture was heated to reflux (oil bath, 90 °C) and allowed to stir for 18 h. Upon cooling to 0 °C,

the amber reaction mass was diluted with $H_2O$ (15 mL) and adjusted to pH 1 using 10 M aq. HCl. The aqueous mixture was then extracted with $CH_2Cl_2$ (10 mL x 3) and the combined organic layers were dried over anhydrous $Na_2SO_4$ and concentrated under reduced pressure to afford an amber oil. Purification of the residue using gradient flash column chromatography (0–50% $Et_2O$ in hexane) yielded a yellow oil that was carried forward to the next reaction step without any further purification. To a dry 10 mL round bottom flask under an Ar atmosphere, containing an ice-cold stirring suspension of $LiAlH_4$ (218 mg, 5.75 mmol) in anhydrous $Et_2O$ (3 mL), was added dropwise a solution of carboxylic acid **9** (451 mg, 2.30 mmol) in anhydrous $Et_2O$ (1.5 mL). Following the complete addition of **9**, the resulting suspension was warmed to room temperature. After stirring for 3 h, the reaction mass was cooled to 0 °C and sequentially treated dropwise with $H_2O$ (0.1 mL), 15% (w/v) aq. NaOH (0.1 mL), and $H_2O$ (0.3 mL), to give a colorless suspension that was diluted with $Et_2O$ (10 mL) and allowed to stir at room temperature for 30 min. The mixture was then filtered through a short pad of Celite® and carefully concentrated under reduced pressure to afford a colorless residue that was further purified by gradient flash column chromatography (0–40% $Et_2O$ in pentane) to yield homoallylic alcohol **5** (365 mg, 87% [over two steps from **8**]): $^1H$ NMR ($CDCl_3$, 400 MHz) mixture of isomers* $\delta$ 5.13–5.05 (m, 2H), 3.62 (overlapping t, $J$ = 6.5 Hz, 1H), 3.61 (t, $J$ = 6.4 Hz, 1H), 2.33–2.27 (m, 2H), 2.09–2.02 (m, 6H), 1.68 (s, 3H), 1.60 (s, 3H), 1.50 (brs, 1H), 1.01 (overlapping t, $J$ = 7.4 Hz, 1.5H), 0.97 (overlapping t, $J$ = 7.6 Hz, 1.5H).

*Note: Both (*E*)-**5** and (*Z*)-**5** stereoisomers are described.

### 5-[(4-Ethyl-8-methylnona-3,7-dien-1-yl)thio]-1-methyl-1*H*-tetrazole (10)

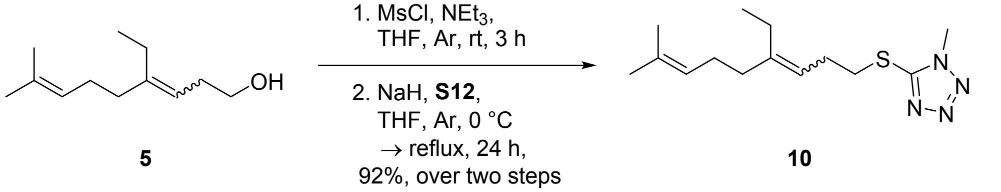


To a dry 25 mL round bottom flask under an Ar atmosphere, containing a solution of homoallylic alcohol **5** (215 mg, 1.18 mmol) in anhydrous THF (3.54 mL), was added MsCl (0.11 mL, 1.42 mmol) and $NEt_3$ (0.21 mL, 1.53 mmol). After stirring at room temperature for 3 h, the resulting mixture was cooled to 0 °C and charged with a suspension of 1-methyl-5-mercaptotetrazole (**S12**, 178 mg, 1.53 mmol) and NaH (60% [w/w] dispersion in mineral oil, 123 mg, 1.53 mmol) in anhydrous THF (5 mL) that had previously been aged at 0 °C for 30 min. Following the complete addition of **S12** sodium salt, the mixture was heated to reflux (oil bath, 70 °C). After stirring at

reflux for 24 h, the reaction mass was cooled to room temperature and charged with sat. aq. $NH_4Cl$ (10 mL). The mixture was then extracted with EtOAc (10 mL x 3) and the combined organic layers were washed with brine (10 mL), dried over anhydrous $MgSO_4$, and concentrated under reduced pressure to afford a residue that was further purified by gradient flash column chromatography (0–20% EtOAc in hexane) to yield title compound **10** as a colorless oil (303 mg, 92% [over two steps from **5**]): $^1$H NMR ($CDCl_3$, 400 MHz) mixture of isomers* $\delta$ 5.16–5.08 (m, 2H), 3.90 (s, 3H), 3.35 (overlapping t, *J* = 7.2 Hz, 1H), 3.35 (overlapping t, *J* = 7.3 Hz, 1H), 2.52 (q, *J* = 7.3 Hz, 2H), 2.06–2.00 (m, 6H), 1.68–1.67 (m, 3H), 1.60–1.59 (m, 3H), 0.98 (overlapping t, *J* = 7.4 Hz, 1.5H), 0.95 (overlapping t, *J* = 7.6 Hz, 1.5H).

*Note: Both (*E*)-**10** and (*Z*)-**10** stereoisomers are described.

**5-[(4-Ethyl-8-methylnona-3,7-dien-1-yl)sulfonyl]-1-methyl-1*H*-tetrazole (11)**

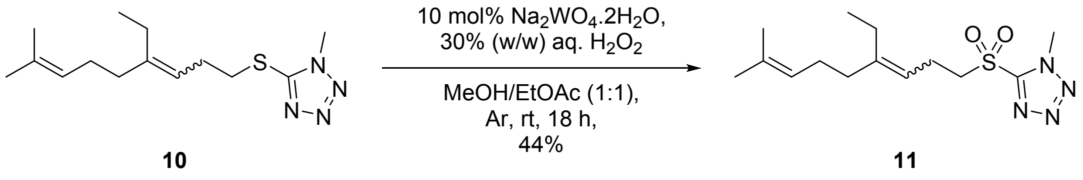


To a dry 10 mL round bottom flask under an Ar atmosphere, containing tetrazole **10** (298 mg, 1.06 mmol) in a mixture of MeOH (1.05 mL) and EtOAc (1.05 mL), was added $Na_2WO_4$ (36 mg, 0.11 mmol) and 30% (w/w) aq. $H_2O_2$ (0.95 mL, 9.33 mmol). After stirring at room temperature for 18 h, the reaction mass was cooled to 0 °C and slowly treated with sat. aq. $Na_2SO_3$ (10 mL). The mixture was then extracted with $CH_2Cl_2$ (5 mL x 3). The combined organic extracts were dried over anhydrous $MgSO_4$ and concentrated under reduced pressure to give a residue that was purified using gradient flash column chromatography (0–15% EtOAc in hexane) to yield title compound **11** as a colorless oil (146 mg, 44%): $^1$H NMR ($CDCl_3$, 400 MHz) mixture of isomers* $\delta$ 5.11–5.00 (m, 2H), 4.35 (s, 3H), 4.35 (s, 3H), 3.69–3.64 (m, 2H), 2.68–2.62 (m, 2H), 2.08–2.00 (m, 6H), 1.68 (s, 3H), 1.60–1.59 (m, 3H).

*Note: Both (*E*)-**11** and (*Z*)-**11** stereoisomers are described.

**14-Methyl-α-farnesene (1a–d)**

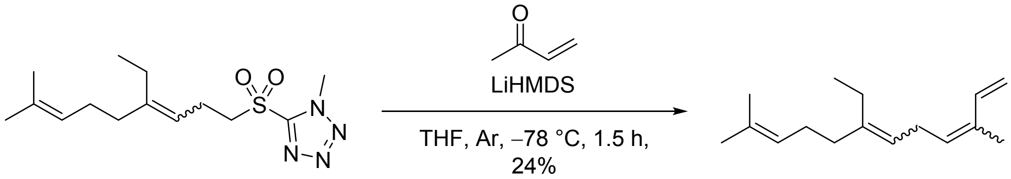


**11** **1a–d**

To a dry 10 mL round bottom flask under an Ar atmosphere, containing sulfone **11** (144 mg, 0.46 mmol) and methyl vinyl ketone (38 µL, 0.47 mmol) in anhydrous THF (1.57 mL) at −78 °C was added LiHMDS (1 M in THF, 0.52 mL, 0.52 mmol). The mixture was then treated with an additional portion of LiHMDS (1 M in THF, 0.19 mL, 0.19 mmol) at 30 min intervals (x 2). Following the complete addition of the third portion of LiHMDS, the reaction was allowed to stir at −78 °C for a further 30 min, prior to the addition of sat. aq. $NH_4Cl$ (5 mL). The reaction mass was then repeatedly extracted with pentane (2 mL x 5) and the combined organic layers dried over anhydrous $MgSO_4$. Careful concentration of the resulting organic phase under a gentle stream of Ar gave a residue that was further purified by isocratic flash column chromatography (pentane) to give title compound (**1a**–**d**) as a colorless oil (25 mg, 24%): $^1H$ NMR ($CDCl_3$, 400 MHz) mixture of isomers* *δ* 6.81 (dd, *J* = 17.3, 10.8 Hz, 0.4H), 6.37 (dd, *J* = 17.5, 10.6 Hz, 0.5H), 5.45 (t, *J* = 7.6 Hz, 0.5H), 5.35 (t, *J* = 7.4 Hz, 0.4H), 5.20 (d, *J* = 17.4 Hz, 0.5H), 5.14–5.05 (m, 3H), 4.93 (d, *J* = 10.6 Hz, 0.5H), 2.90–2.83 (m, 2H), 2.09–1.99 (m, 6H), 1.82 (s, 1.3H), 1.76 (s, 1.4H), 1.69–1.68 (m, 3H), 1.61–1.60 (m, 2.8H), 1.54 (s, 1.2H), 1.01–0.96 (m, 3H).

*Note: (*Z,E*)-, (*Z,Z*)-, (*E,Z*)-, and (*E,E*)-stereoisomers **1a**–**d** are described.

## Nominal EI-MS Spectra of 14-methyl-α-farnesene isomers 1a–d

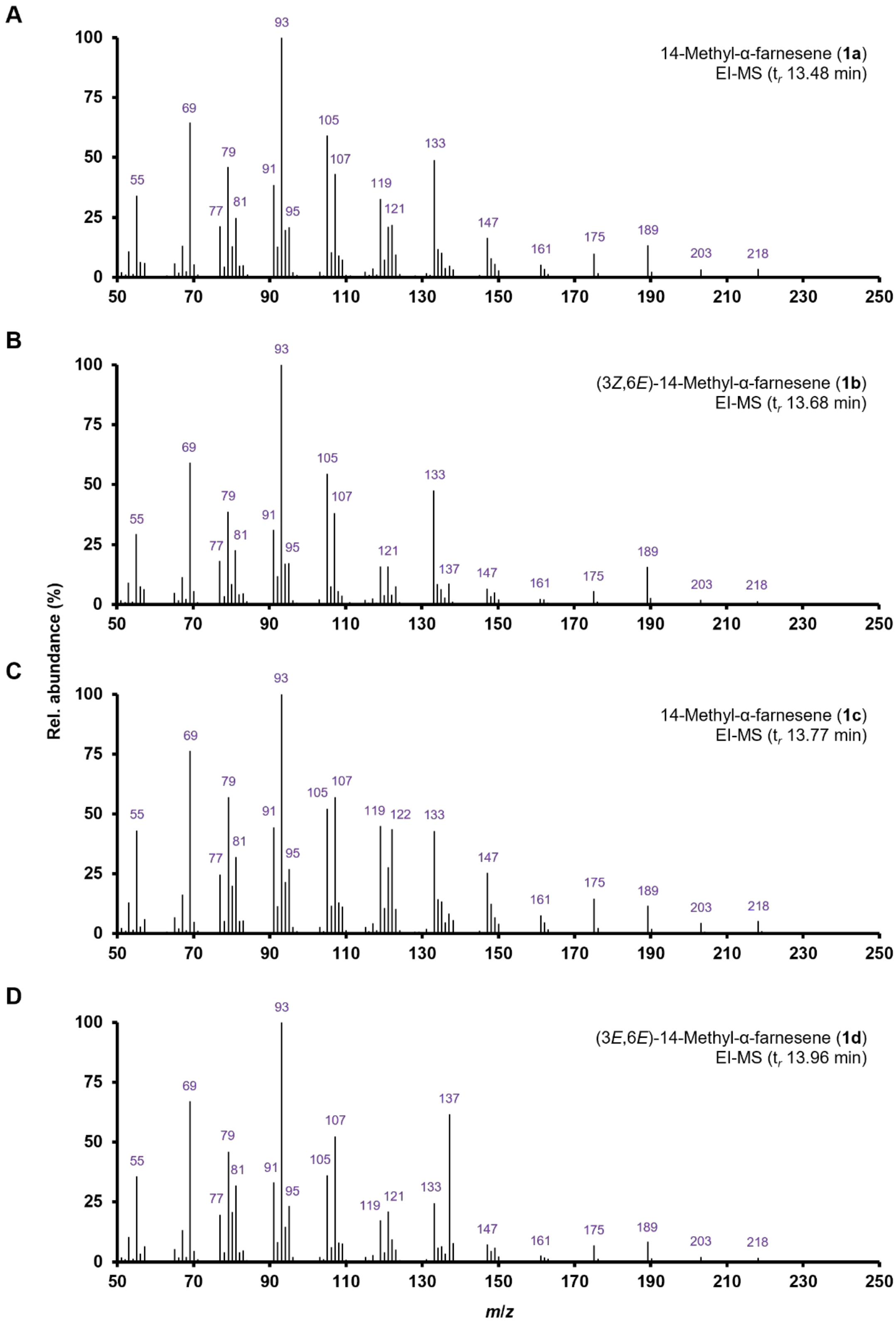


**Figure S5.** Nominal EI-MS spectra of 14-methyl-α-farnesene isomers **1a**–**d**.

## Synthesis of (*E*)-3-Ethyl-7-methylocta-2,6-dien-1-ol ((*E*)-7)

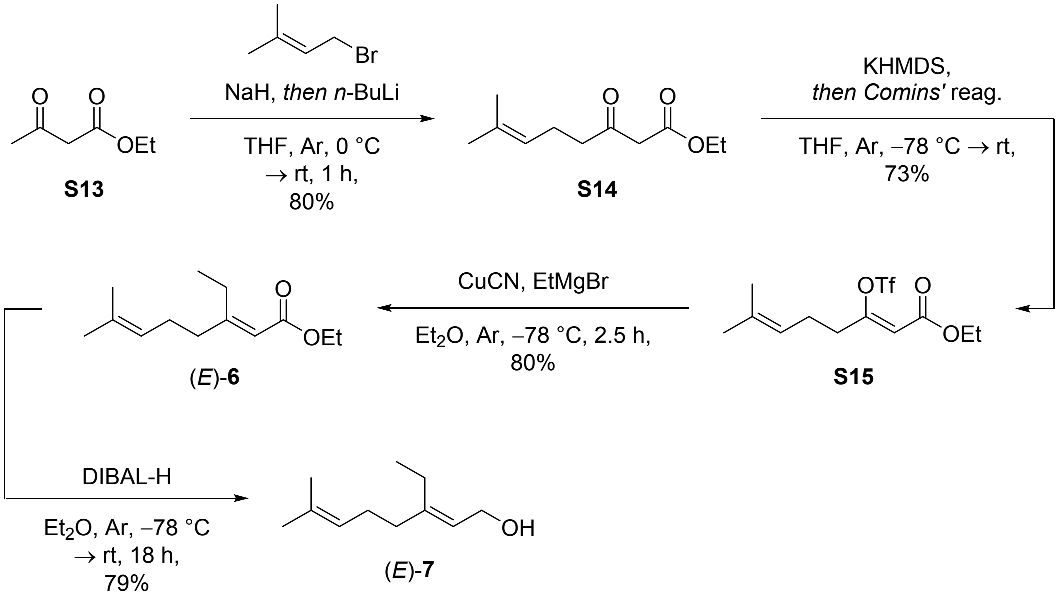


**Scheme S4.** Following Gibb's previously reported route,[8] known allyl alcohol (*E*)-**7** was prepared over four steps from commercially available ethyl acetoacetate (**S13**).

## Ethyl 7-methyl-3-oxooct-6-enoate (S14)

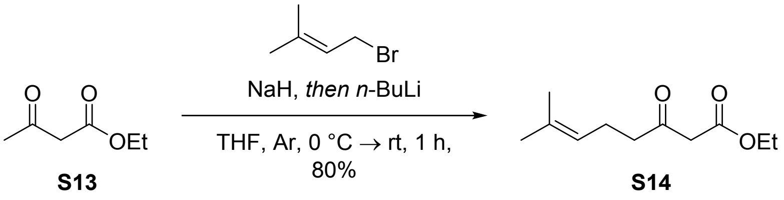


Following a previously reported procedure,[10] to a dry 100 mL round bottom flask under an Ar atmosphere was added ethyl acetoacetate (**S13**, 1.27 mL, 10 mmol) and anhydrous THF (10 mL). Upon cooling to 0 °C, the colorless solution was charged with NaH (60% [w/w] dispersion in mineral oil, 440 mg, 11 mmol) and allowed to stir at 0 °C for 10 min. To the resulting mixture was slowly added *n*-BuLi (2.5 M in hexane, 4.2 mL, 10.5 mmol) to give a bright yellow solution that was stirred for a further 10 min at 0 °C and then sequentially charged dropwise with prenyl bromide (1.27 mL, 11 mmol) and warmed to room temperature. After stirring for 1 h, the yellow reaction mass was quenched with sat. aq. $NH_4Cl$ (10 mL) and further diluted with $H_2O$ (10 mL) and $Et_2O$ (20 mL) and separated. The resulting aqueous layer was repeatedly extracted with $Et_2O$ (10 mL x 2) and the combined organic layers were dried over anhydrous $MgSO_4$ and concentrated under reduced pressure to afford an oil that was further purified by gradient flash column

chromatography (0–5% $Et_2O$ in hexane) to yield title compound **S14** as a colorless oil (1.593 g, 80%): $R_f$ 0.17 ($Et_2O$/hexane, 1:9); $^1H$ NMR ($CDCl_3$, 400 MHz) mixture of tautomers* $\delta$ 12.09 (s, 0.07H, enol), 5.08–5.03 (m, 1H), 4.97 (s, 0.08H, enol), 4.22–4.17 (m, 4.22–4.15), 3.42 (s, 2H), 2.56 (t, $J$ = 7.40 Hz, 2H), 2.30–2.25 (m, 2H), 1.67 (s, 3H), 1.61 (s. 3H), 1.58–1.51 (m, 1H), 1.30–1.24 (m, 3H). Physical and spectral data agreed with those reported previously.[10]

*$^1H$ NMR analysis indicated a keto:enol ratio of 12:1.

### Ethyl (*Z*)-7-methyl-3-{[(trifluoromethyl)sulfonyl]oxy}octa-2,6-dienoate (S15)

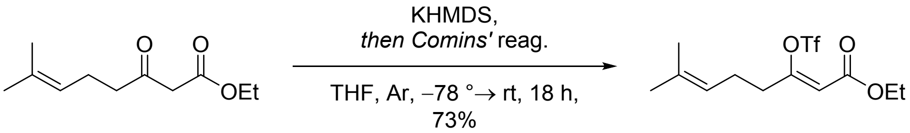


**S14** **S15**

To a dry 100 mL round bottom flask under an Ar atmosphere, containing a solution of β-keto ester **S14** (1.317 g, 6.64 mmol) in anhydrous THF (8.3 mL) cooled to −78 °C, was added 1 M KHMDS in THF (7.97 mL, 7.97 mmol). After stirring for 10 min at −78 °C, the resulting mixture was charged with 2-[*N,N*-bis(trifluoromethylsulfonyl)amino]-5-chloropyridine (3.130 g, 7.97 mmol) and allowed to warm to room temperature. Upon stirring at room temperature for 18 h, the resulting brown solution was diluted with $Et_2O$ (30 mL) and successively washed with 10% (w/v) aq. citric acid (30 mL x 2) and $H_2O$ (10 mL). The organic phase was dried over anhydrous $MgSO_4$ and passed through a short pad of Celite® to give a filtrate that was concentrated under reduced pressure to afford an orange suspension. Purification of the residue by gradient flash column chromatography (0–10% $Et_2O$ in hexane) gave title compound **S15** as a yellow oil (1.605 g, 73%): $R_f$ 0.10 ($Et_2O$/hexane, 1:9); $^1H$ NMR ($CDCl_3$, 400 MHz) $\delta$ 5.74 (s, 1H), 5.07–5.03 (m, 1H), 4.25 (q, $J$ = 7.1 Hz, 2H), 2.40 (t, $J$ = 7.4 Hz, 2H), 2.25 (q, $J$ = 7.2 Hz, 2H), 1.70 (s, 3H), 1.62 (s, 3H), 1.31 (t, $J$ = 7.1 Hz, 3H). Physical and spectral data agreed with those reported previously.[8]

### Ethyl (*E*)-3-ethyl-7-methylocta-2,6-dienoate ((*E*)-6)

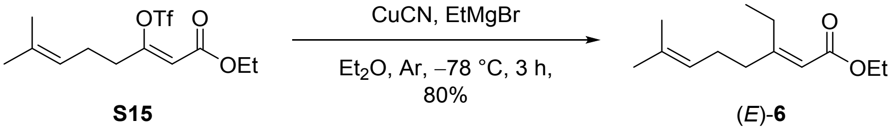


**S15** (*E*)-**6**

To a dry 100 mL round bottom flask under an Ar atmosphere, containing a suspension of CuCN (1.108 g, 12.37 mmol) in anhydrous $Et_2O$ (16 mL) cooled to −78 °C, was charged dropwise with

2 M EtMgCl in $Et_2O$ (3.79 mL, 7.57 mmol). Upon warming to 0 °C and stirring for 5 min, the resulting brown suspension was again cooled to −78 °C and treated dropwise with a solution of triflate **S15** (1.601 g, 4.85 mmol) in anhydrous $Et_2O$ (16 mL). After stirring at −78 °C for 3 h, the reaction mass was slowly diluted with sat. aq. $NH_4Cl$ (15 mL) and repeatedly extracted with $Et_2O$ (15 mL x 3). The combined organic layers were dried over anhydrous $MgSO_4$ and carefully concentrated under reduced pressure to afford a green residue that was subsequently purified using gradient flash column chromatography (0–2% $Et_2O$ in hexane) to give title compound (*E*)-**6** as a colorless oil (818 mg, 80%): $R_f$ 0.43 ($Et_2O$/hexane, 1:19); $^1$H NMR ($CDCl_3$, 400 MHz) $\delta$ 5.61 (s, 1H), 5.10–5.08 (m, 1H), 4.14 (q, $J$ = 7.1 Hz, 2H), 2.61 (q, $J$ = 7.5 Hz, 2H), 2.20 (m, 4H), 1.69 (s, 3H), 1.60 (s, 3H), 1.28 (t, $J$ = 7.1 Hz, 3H), 1.07 (t, $J$ = 7.5 Hz, 3H). Physical and spectral data agreed with those reported previously.[8]

### (*E*)-3-Ethyl-7-methylocta-2,6-dien-1-ol ((*E*)-7)

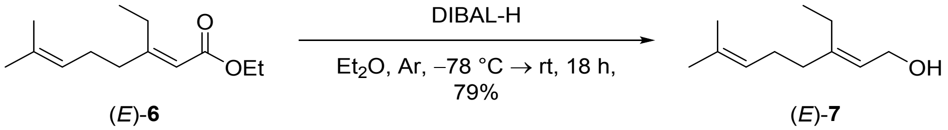


(*E*)-**6** (*E*)-**7**

To a dry 50 mL round bottom flask under an Ar atmosphere, containing ethyl ester (*E*)-**6** (818 mg, 3.89 mmol) in anhydrous $Et_2O$ (7.8 mL) cooled to −78 °C, was added dropwise 1 M DIBAL-H in THF (11.7 mL, 11.7 mmol). After stirring at −78 °C for 18 h, the reaction mass was slowly treated with sat. aq. Na-K-tartrate (25 mL) and allowed to gently stir at room temperature for a further 2 h. The biphasic mixture was separated and the resulting aqueous layer repeatedly extracted with $Et_2O$ (15 mL x 3). The combined organic phases were dried over anhydrous $MgSO_4$ and concentrated under reduced pressure to afford a colorless residue that was further purified by gradient flash column chromatography (0–50% $Et_2O$ in pentane) to yield title compound (*E*)-**7** as a colorless oil (517 mg, 79%): $R_f$ 0.43 ($Et_2O$/pentane, 2:3); $^1$H NMR ($CDCl_3$, 400 MHz) $\delta$ 5.37 (t, $J$ = 7.0 Hz, 1H), 5.12–5.09 (m, 1H), 4.16 (d, $J$ = 7.0 Hz, 2H), 2.12–2.02 (m, 6H), 1.69 (s, 3H), 1.61 (s, 3H), 1.28 (brs, 1H), 0.99 (t, $J$ = 7.6 Hz, 3H). Physical and spectral data agreed with those reported previously.[11]

## NMR data

$^1$H NMR (400 MHz, $CDCl_3$) **S1**

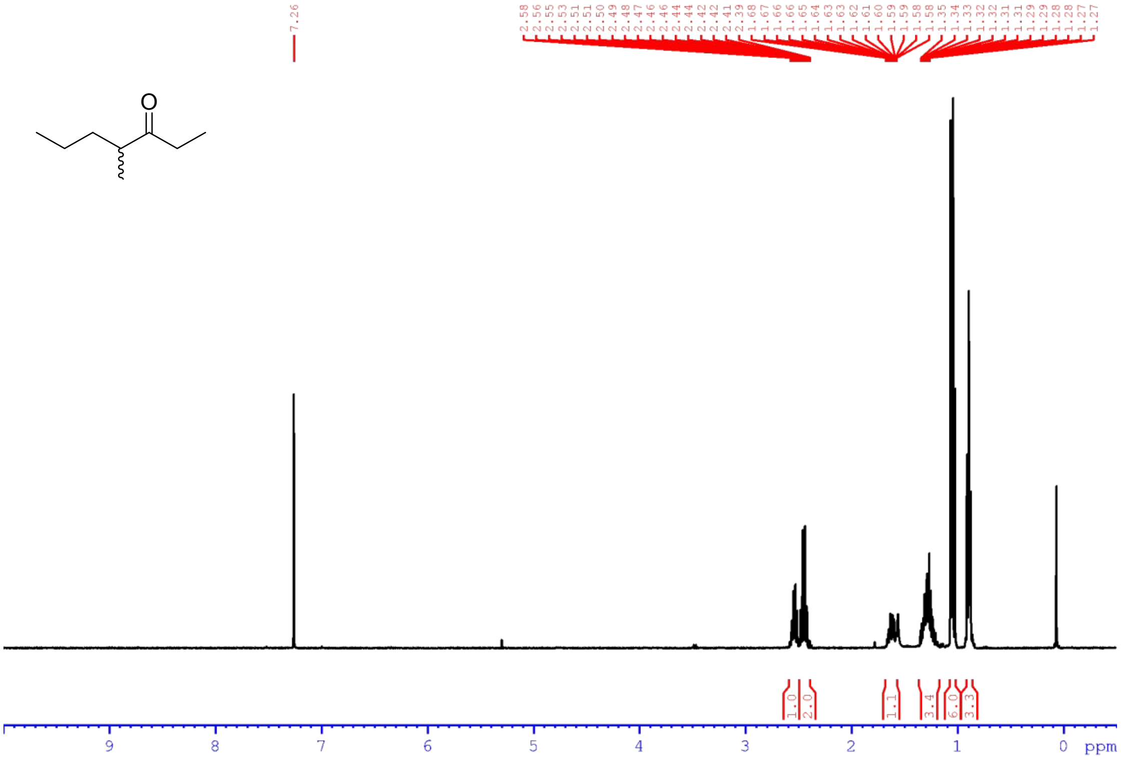

$^1$H NMR (400 MHz, $CDCl_3$) **S5**

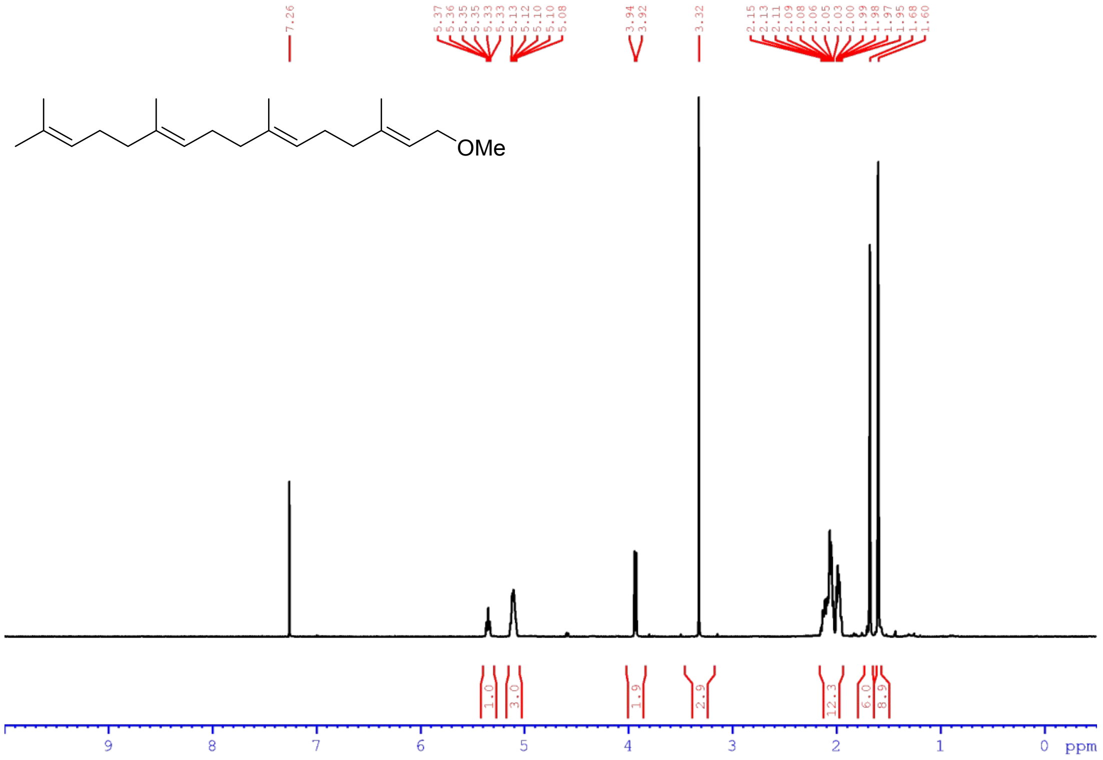

$^{1}$H NMR (400 MHz, $CDCl_3$) **S3**

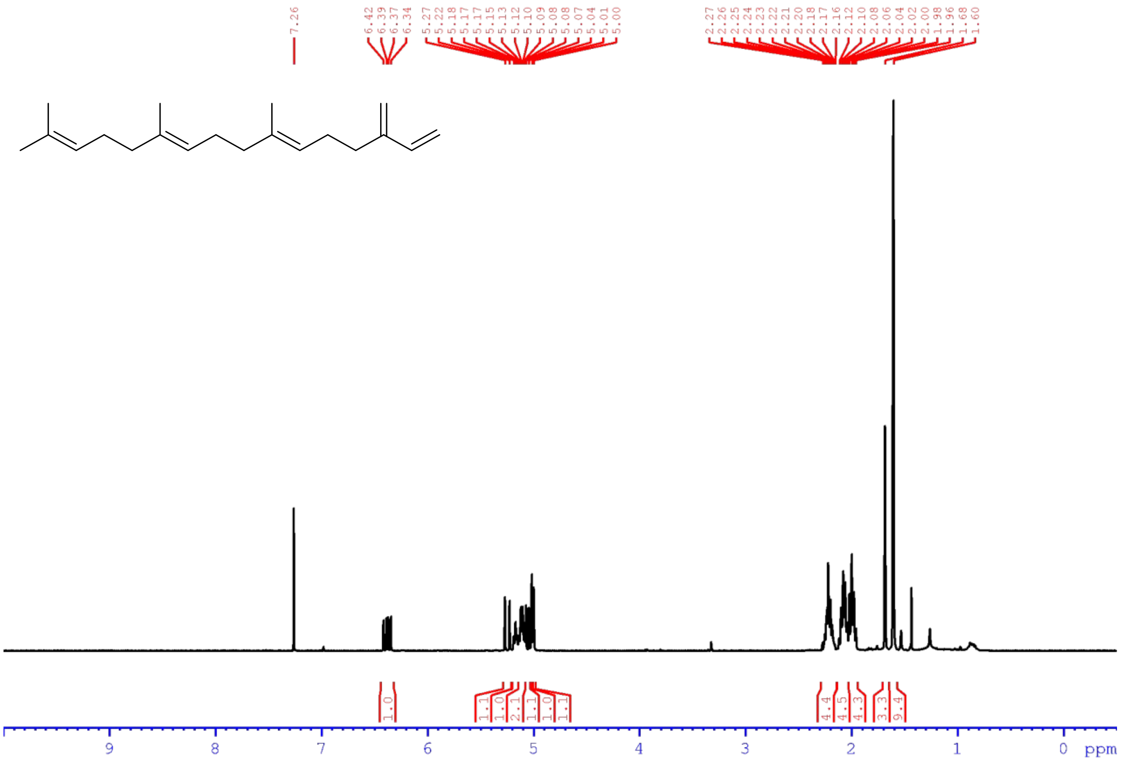


$^{1}$H NMR (400 MHz, $CDCl_3$) **1a–d**

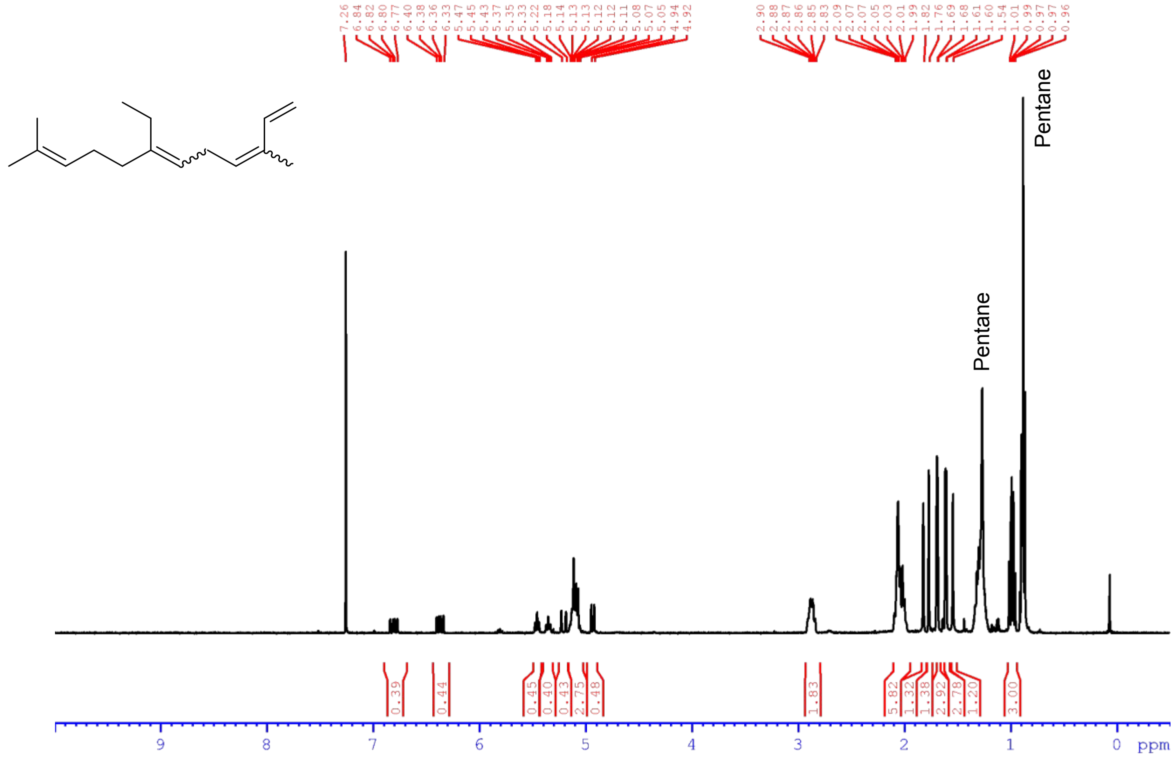

$^{1}H$ NMR (400 MHz, $CDCl_3$) **S14**

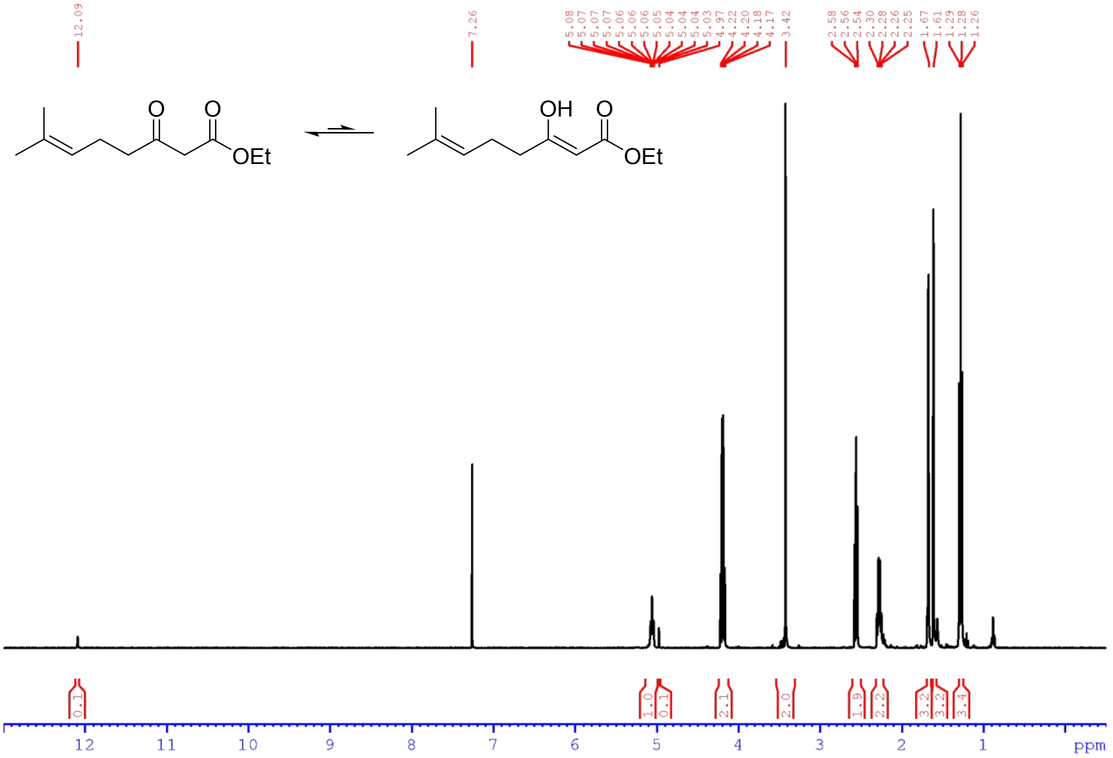


$^{1}H$ NMR (400 MHz, $CDCl_3$) **S15**

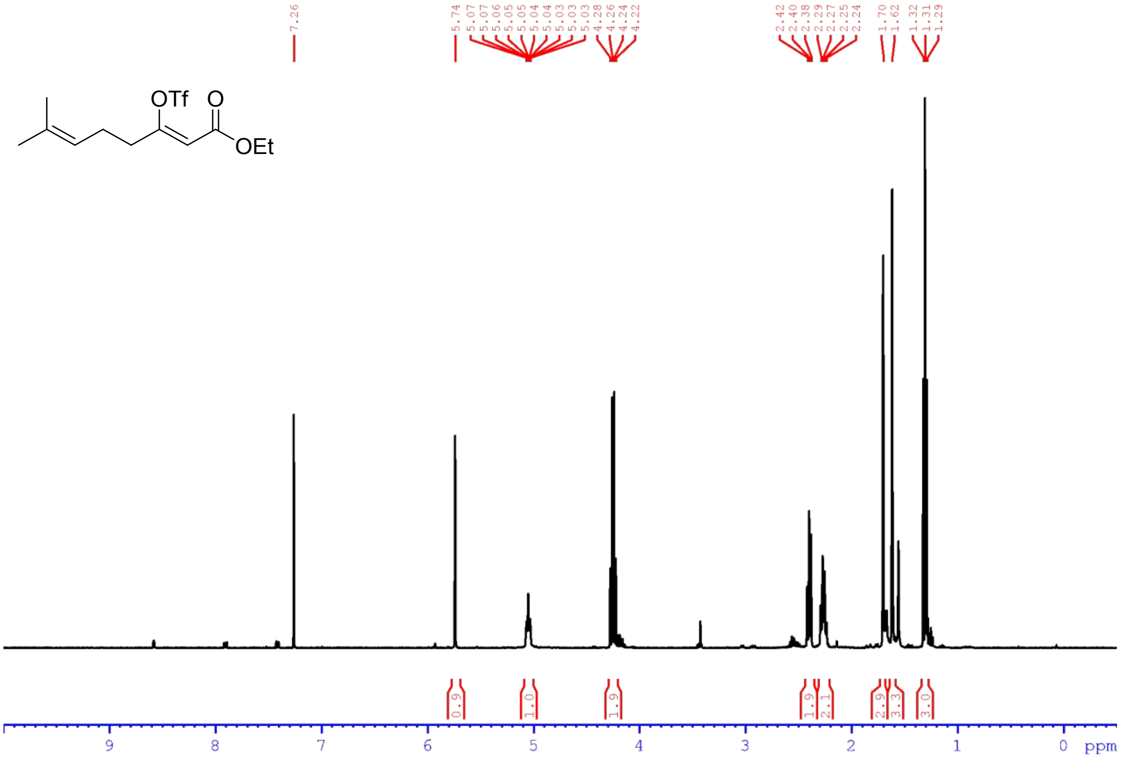

$^{1}H$ NMR (400 MHz, $CDCl_3$) (*E*)-**6**

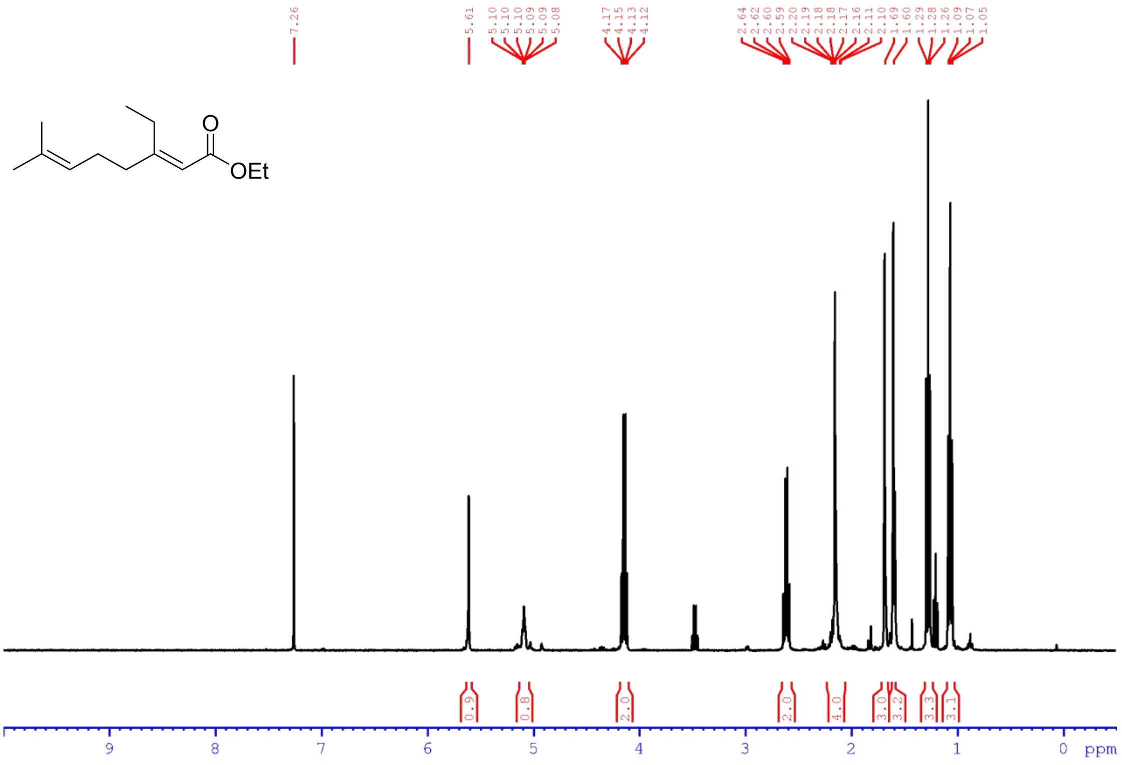

$^{1}H$ NMR (400 MHz, $CDCl_3$) (*E*)-**7**

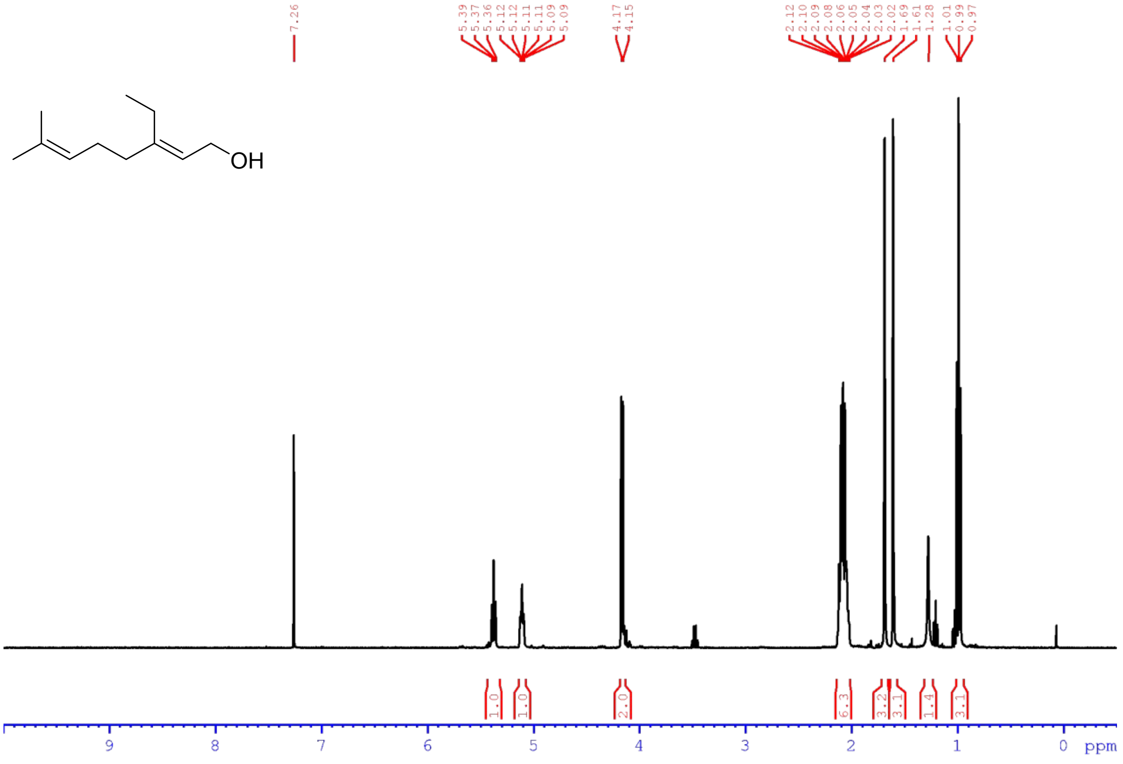

$^1H$ NMR (400 MHz, $CDCl_3$) (*E*)-**8**

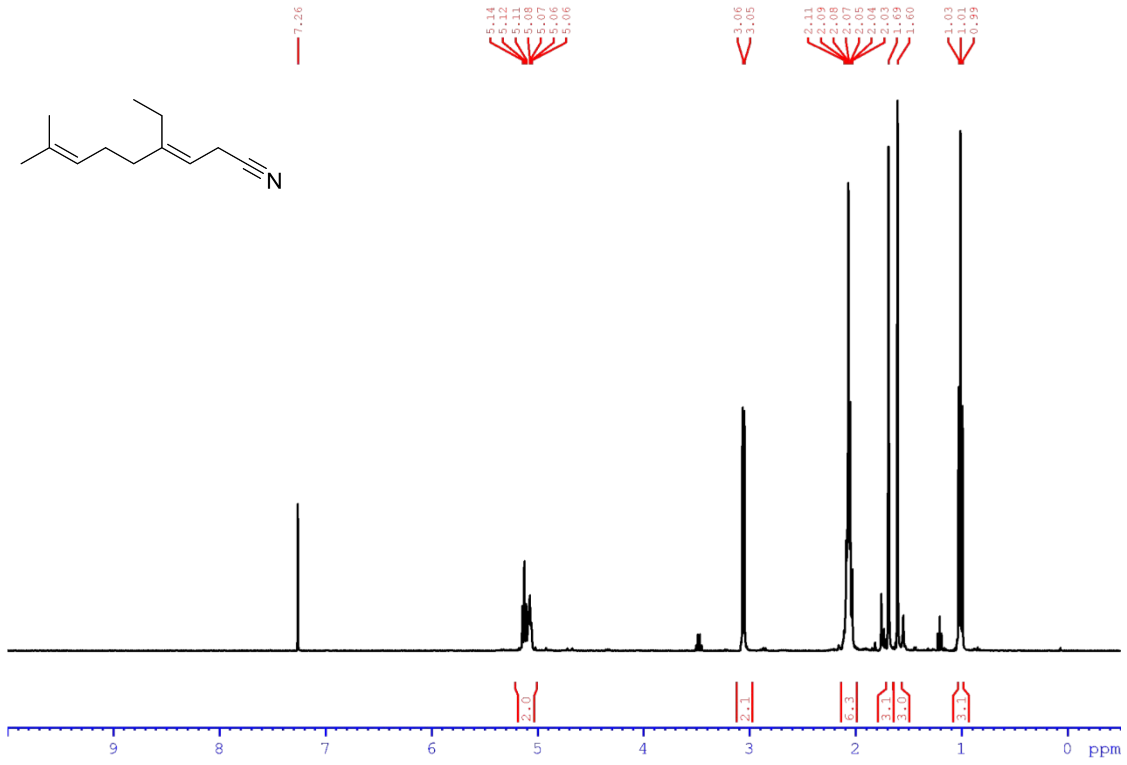


$^{13}C$ NMR (100 MHz, $CDCl_3$) (*E*)-**8**

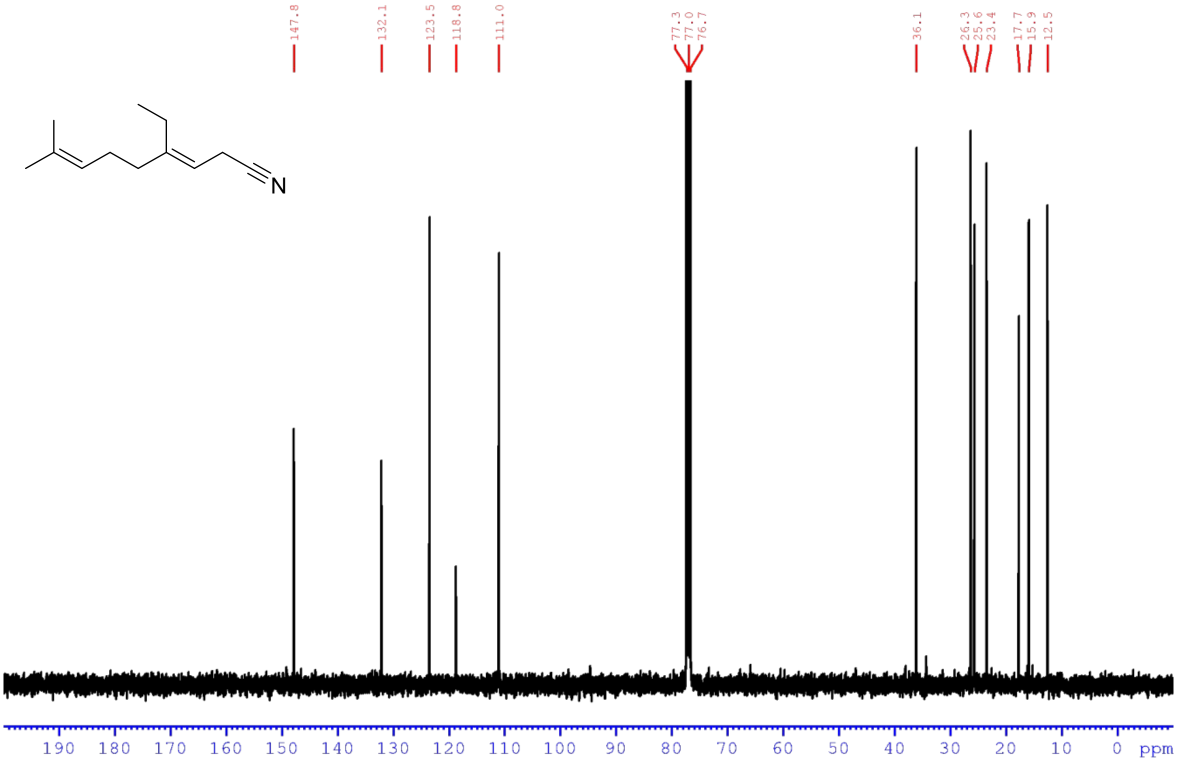

$^{1}H$ NMR (400 MHz, $CDCl_3$) **18**

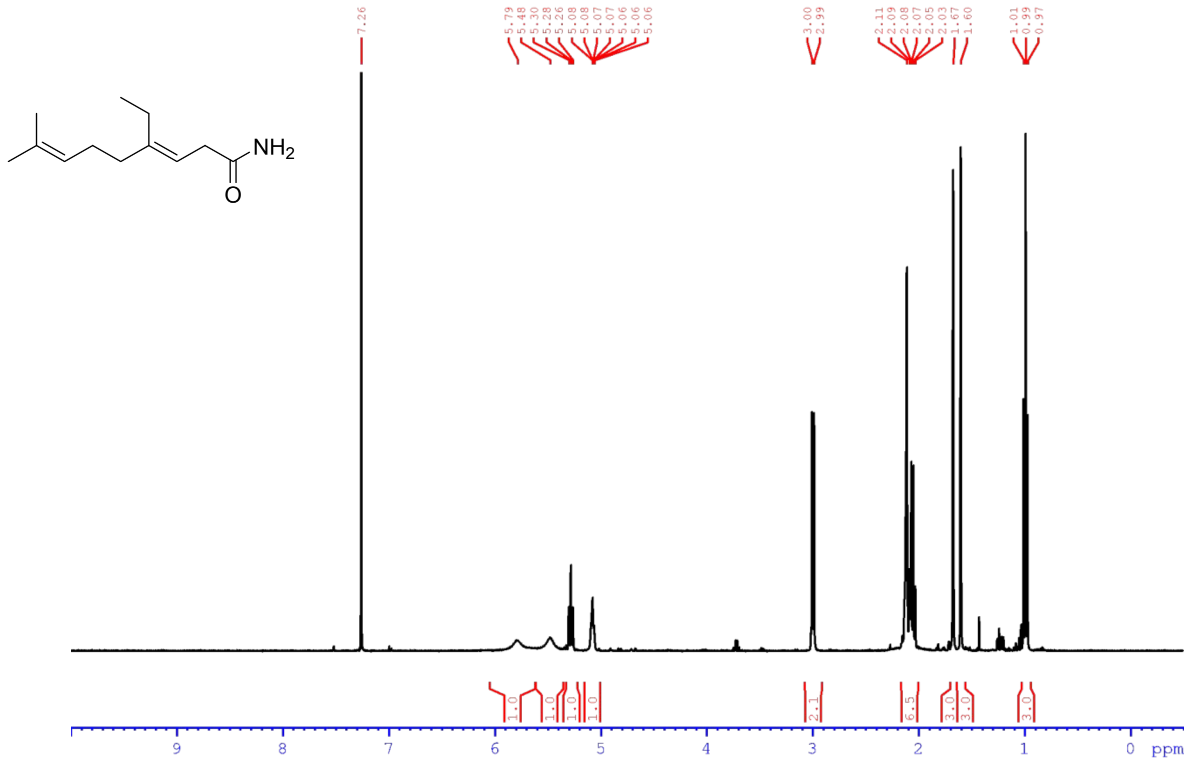


$^{13}C$ NMR (100 MHz, $CDCl_3$) **18**

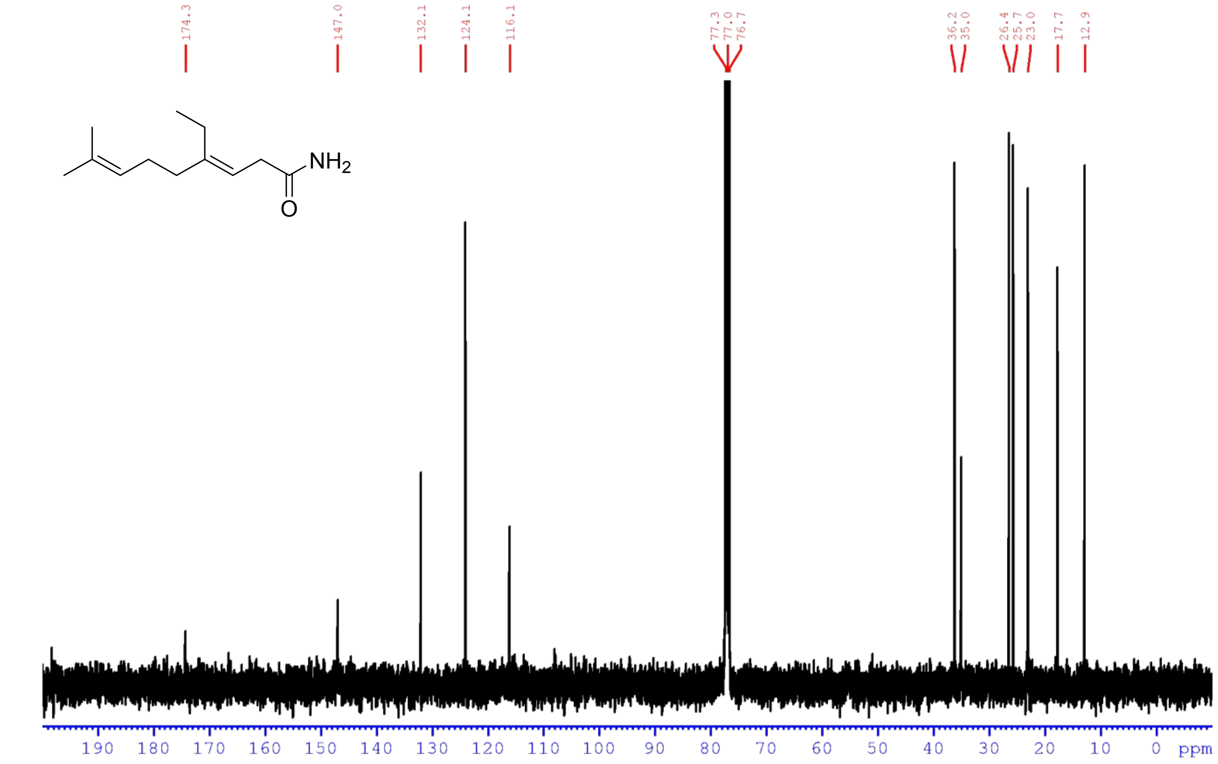

$^{1}$H NMR (500 MHz, $CDCl_3$) (*E*)-**5**

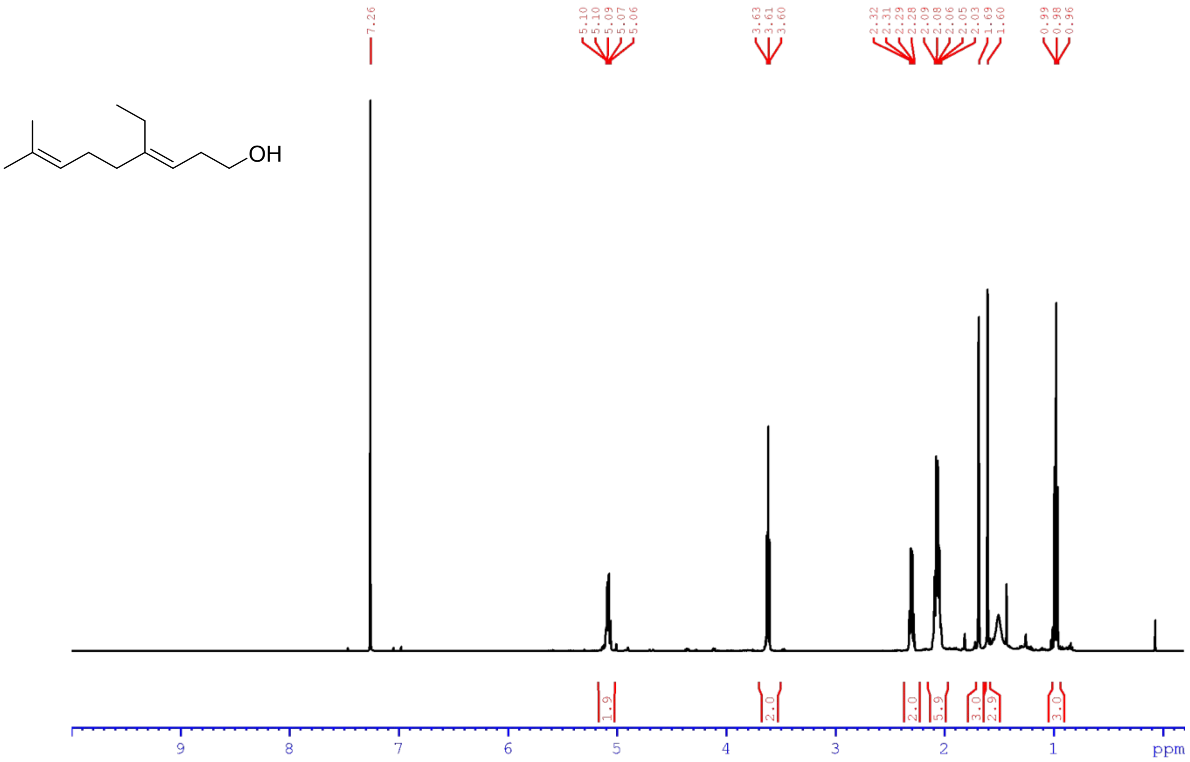


$^{13}$C NMR (125 MHz, $CDCl_3$) (*E*)-**5**

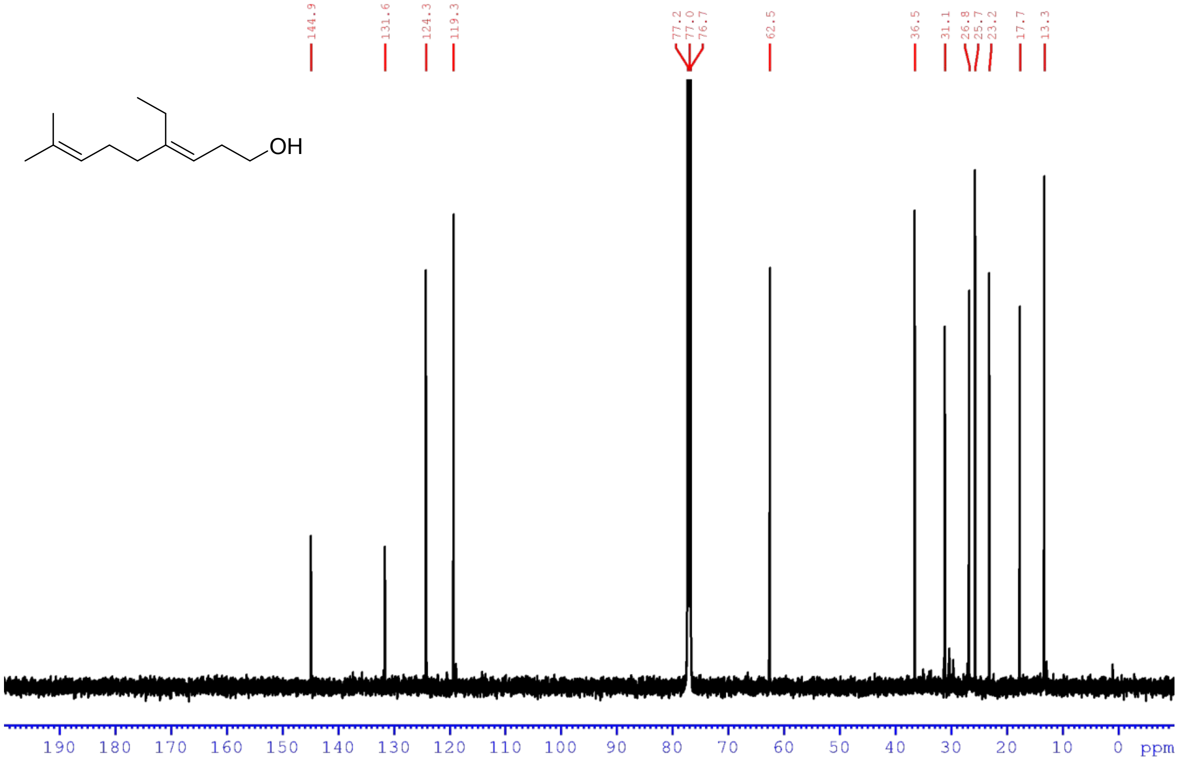

$^{1}$H NMR (500 MHz, $CDCl_3$) **15**

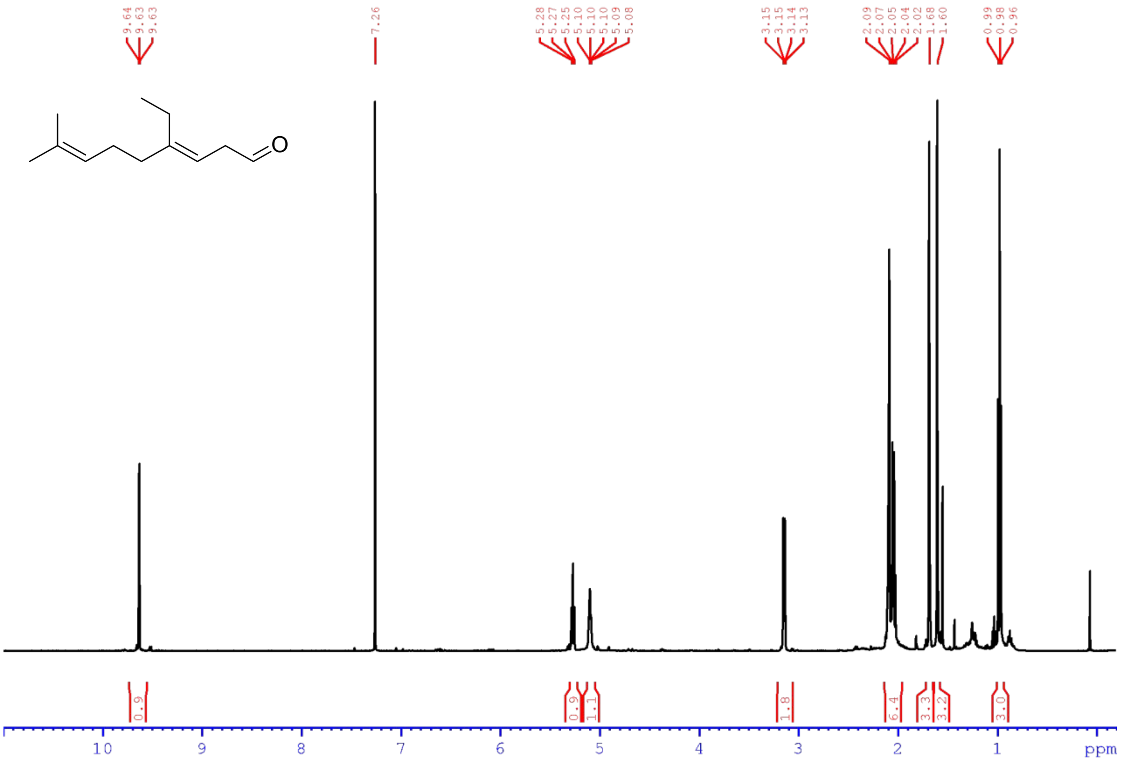


$^{13}$C NMR (125 MHz, $CDCl_3$) **15**

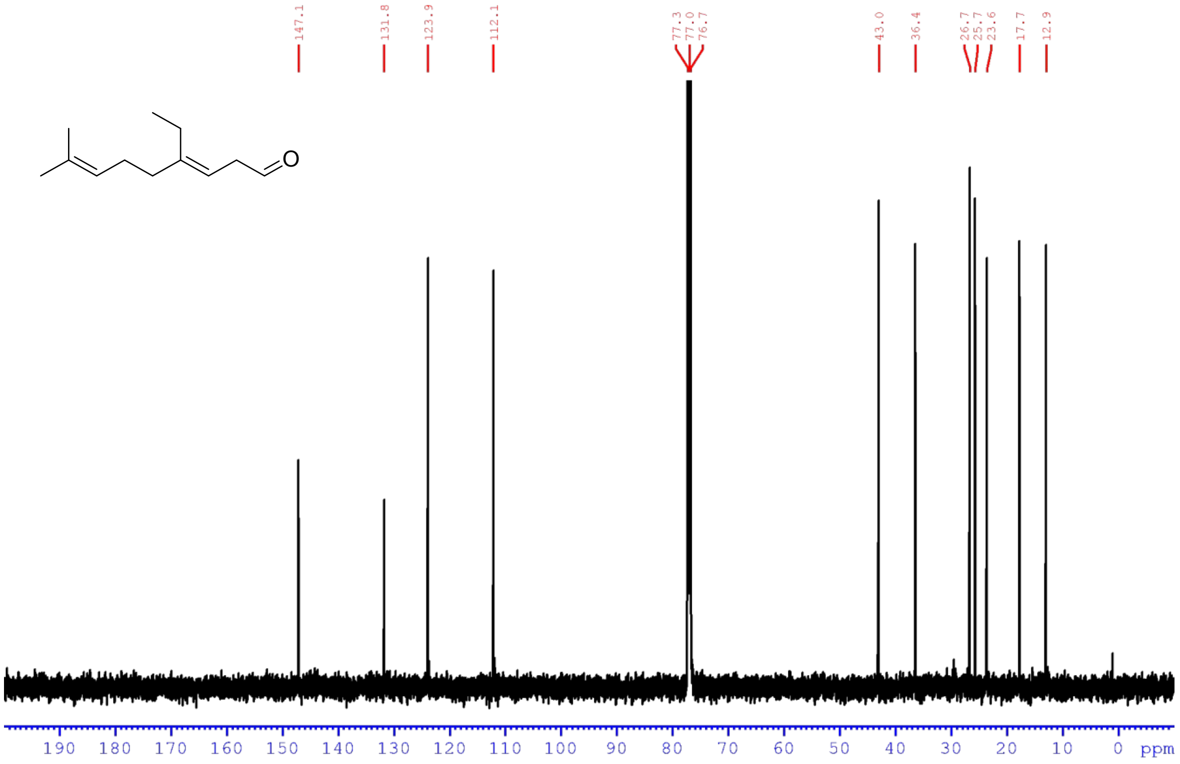

$^{1}H$ NMR (400 MHz, $CDCl_3$) **17**

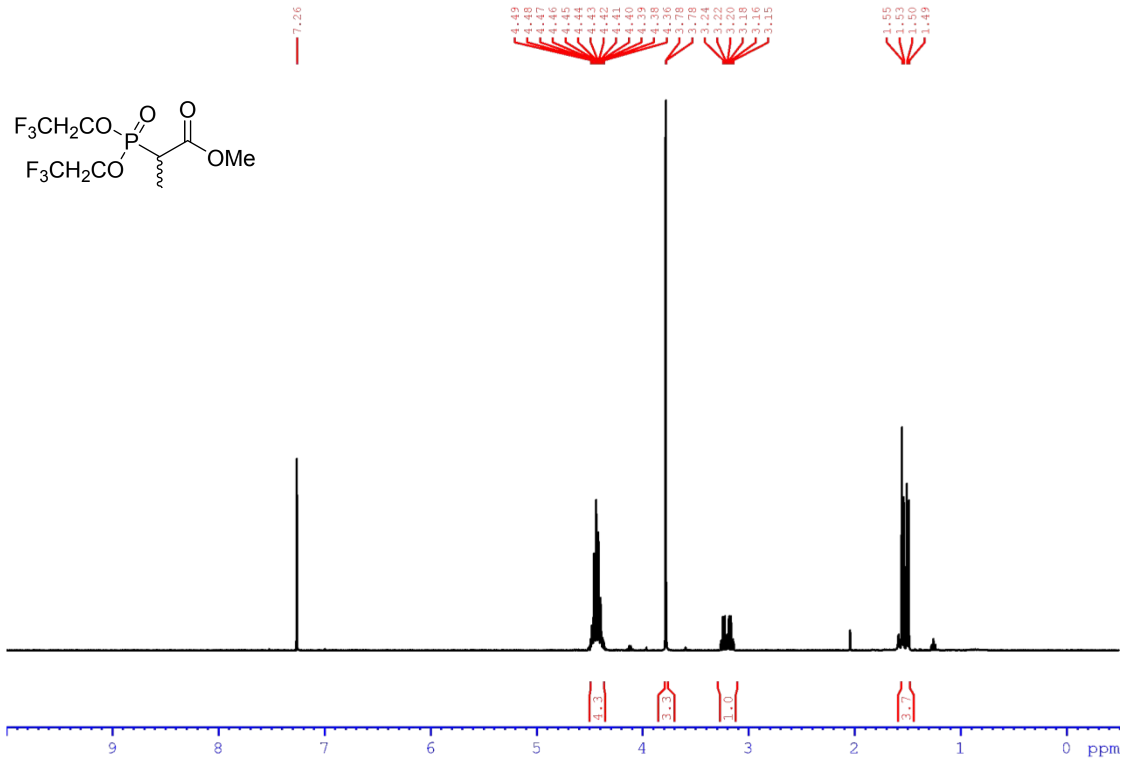


$^{1}H$ NMR (500 MHz, $CDCl_3$) **14**

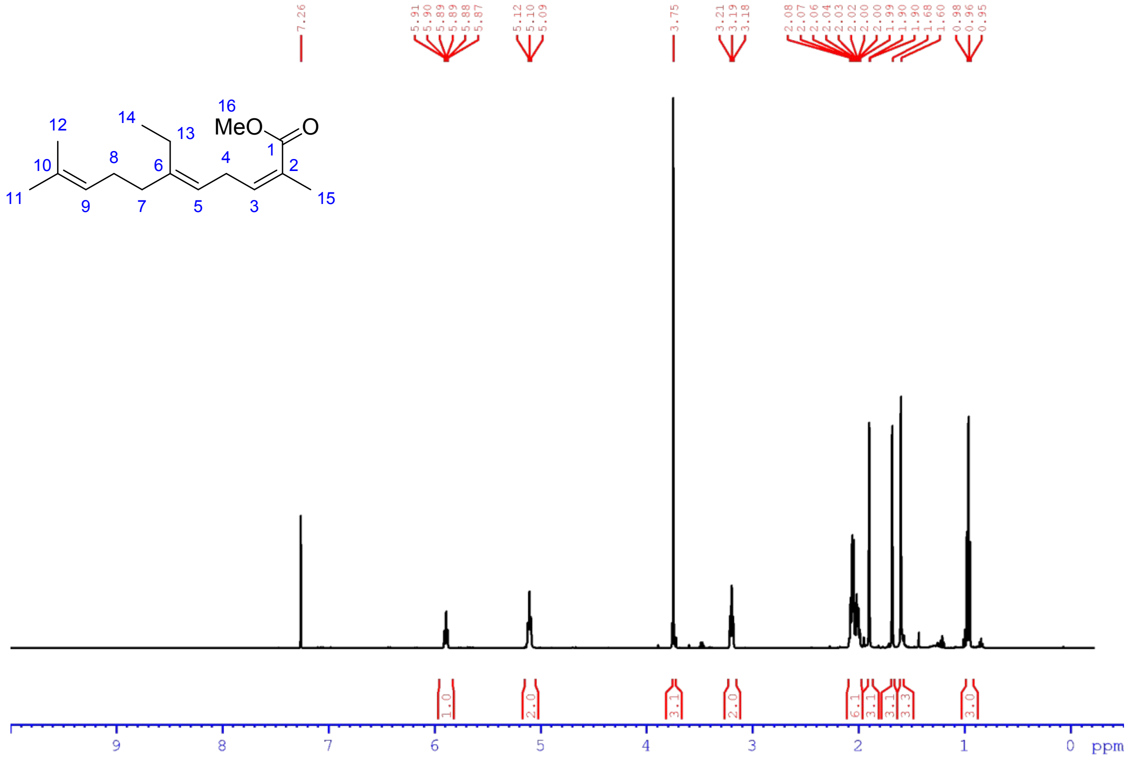

$^{13}$C NMR (125 MHz, $CDCl_3$) **14**

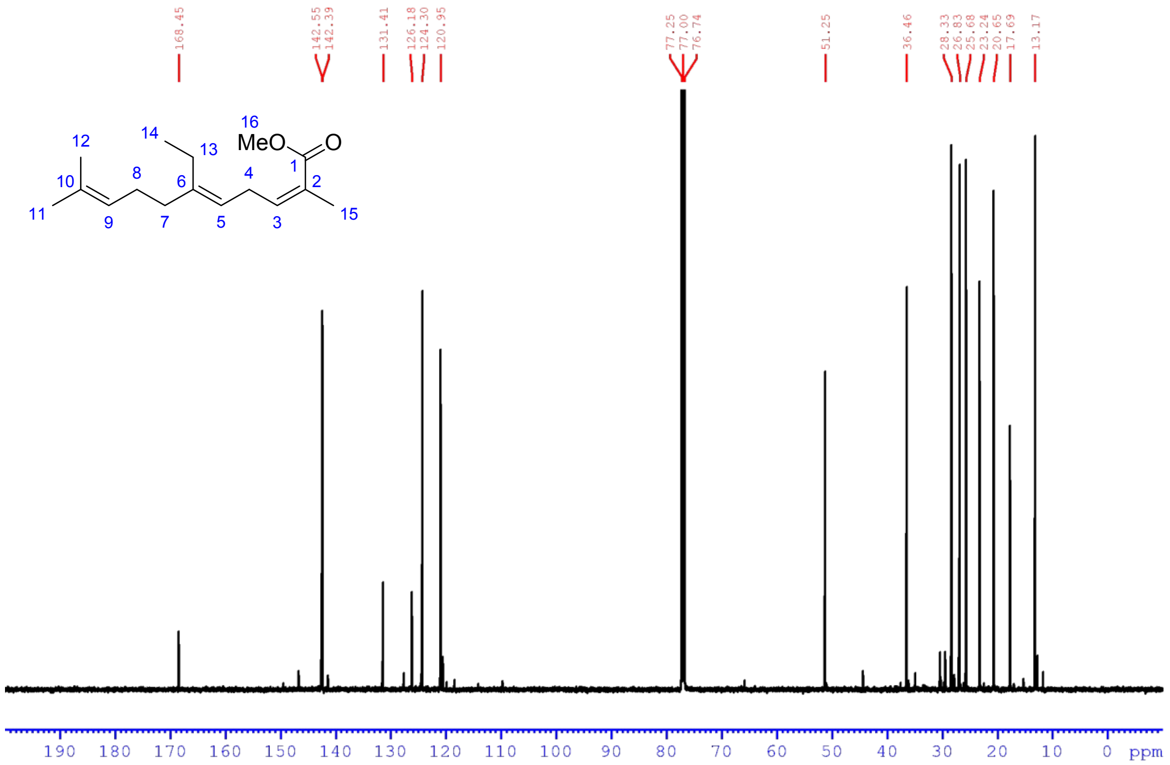


$^{1}$H-$^{1}$H COSY NMR (500 MHz, $CDCl_3$) **14**

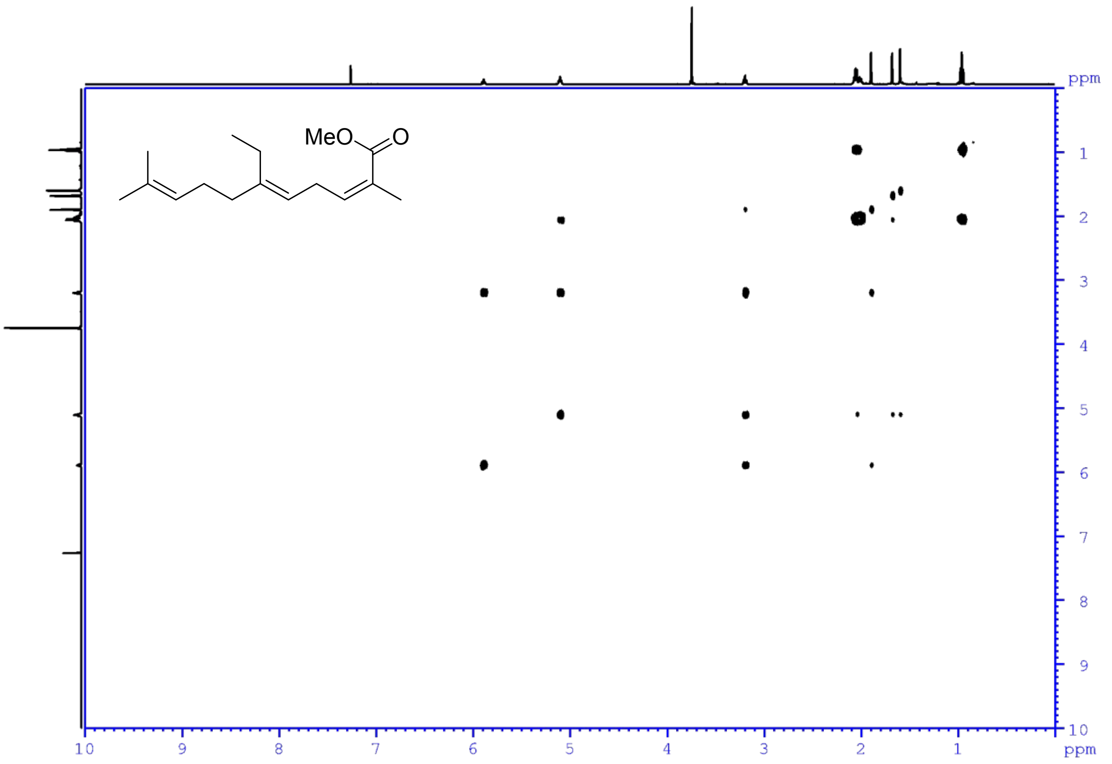

$^{1}H$-$^{13}C$ HSQC (500 MHz, $CDCl_3$) **14**

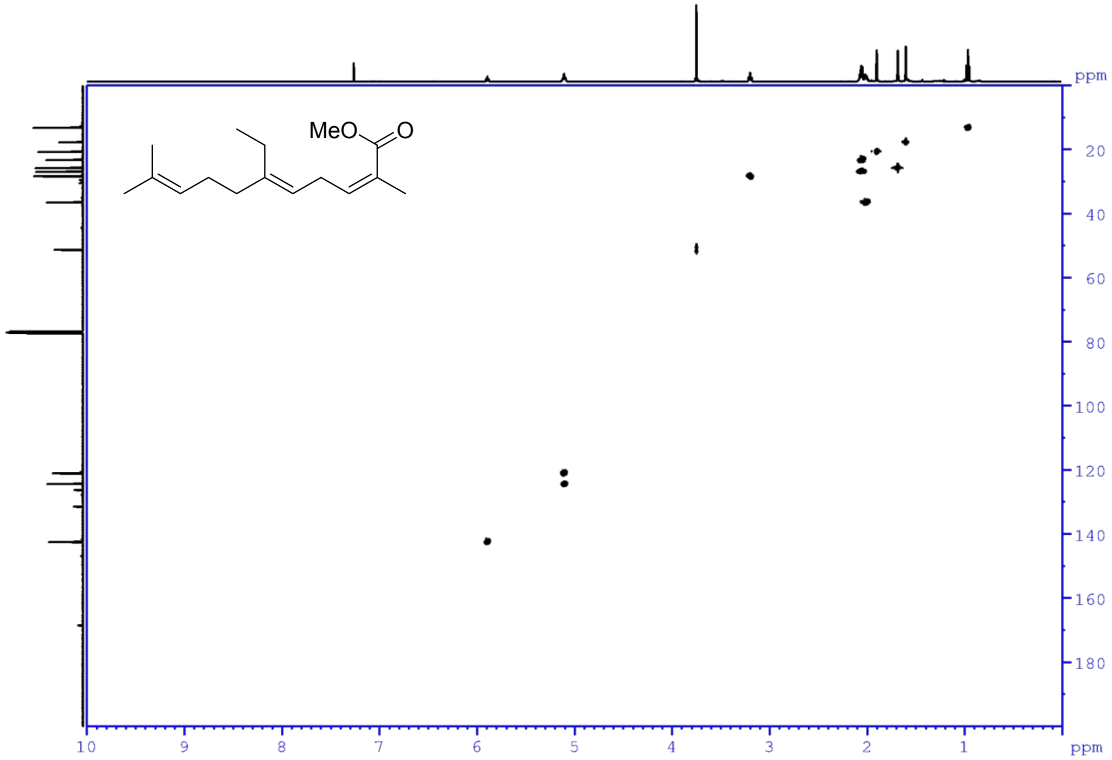


$^{1}H$-$^{13}C$ HMBC (500 MHz, $CDCl_3$) **14**

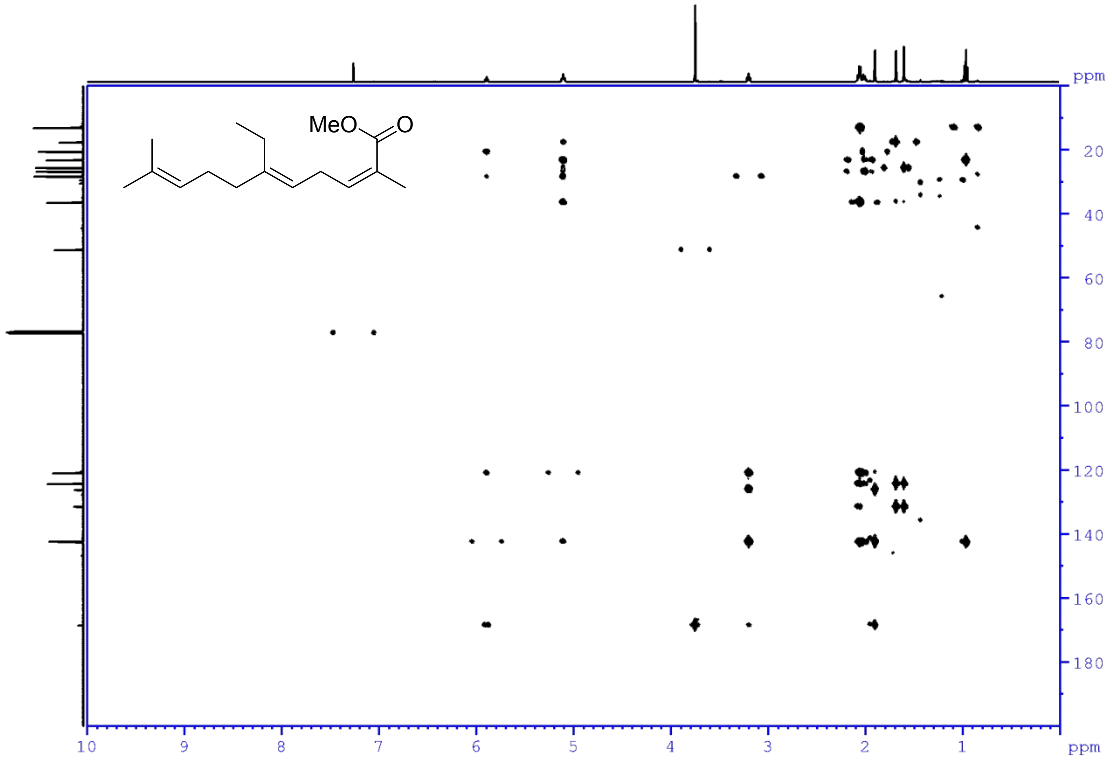

$^1H$-$^1H$ ROESY (500 MHz, $CDCl_3$) **14**

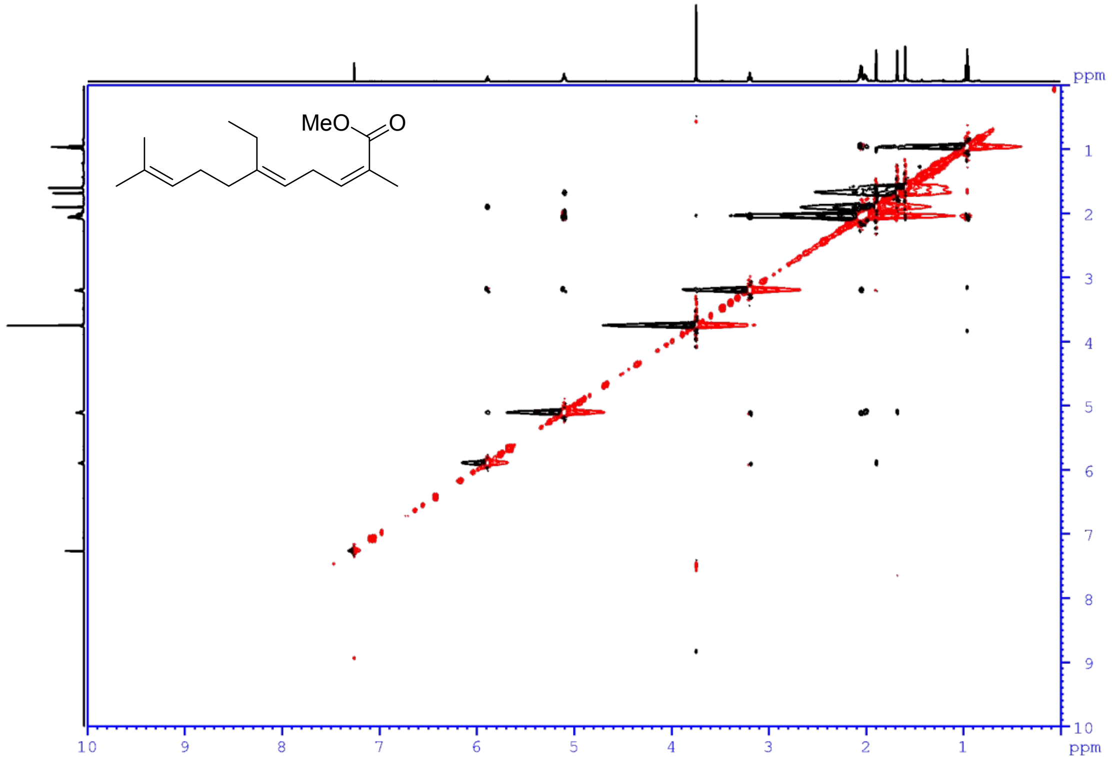

$^1H$ NMR (500 MHz, $CDCl_3$) **19**

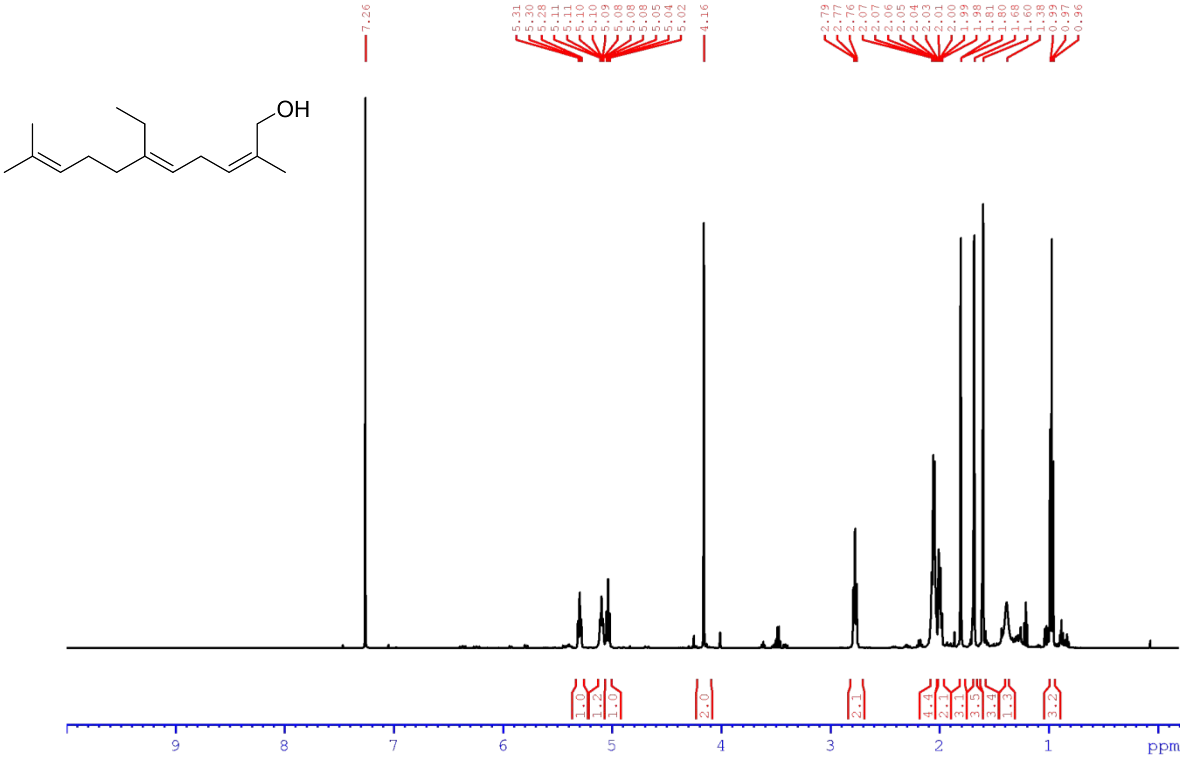

$^{13}C$ NMR (125 MHz, $CDCl_3$) **19**

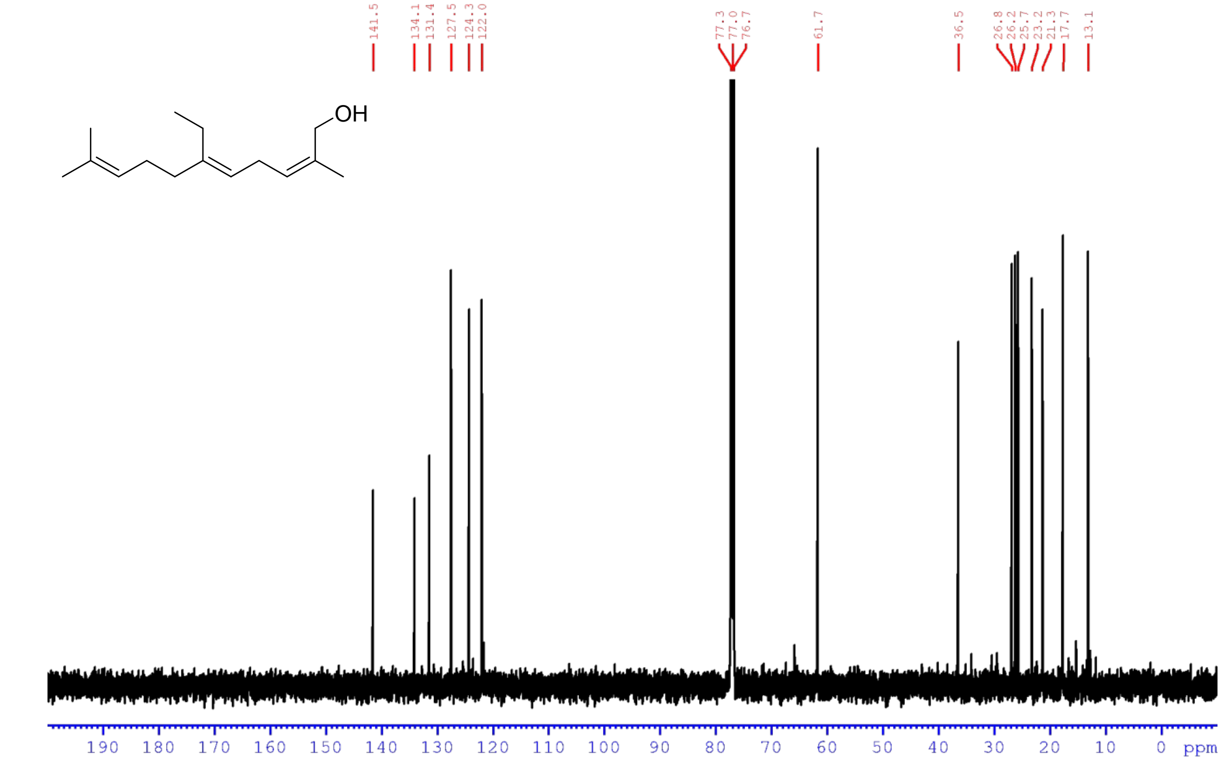


$^{1}H$ NMR (500 MHz, $CDCl_3$) **1b**

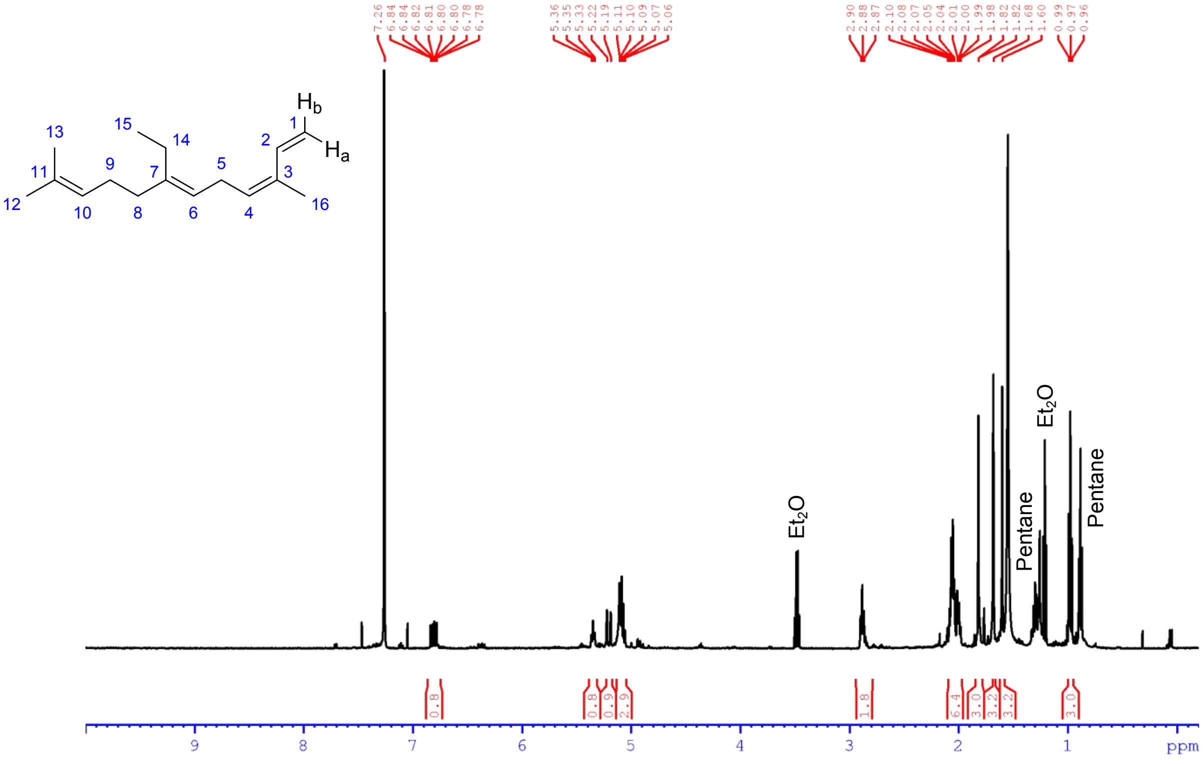

$^{13}C$ NMR (125 MHz, $CDCl_3$) **1b**

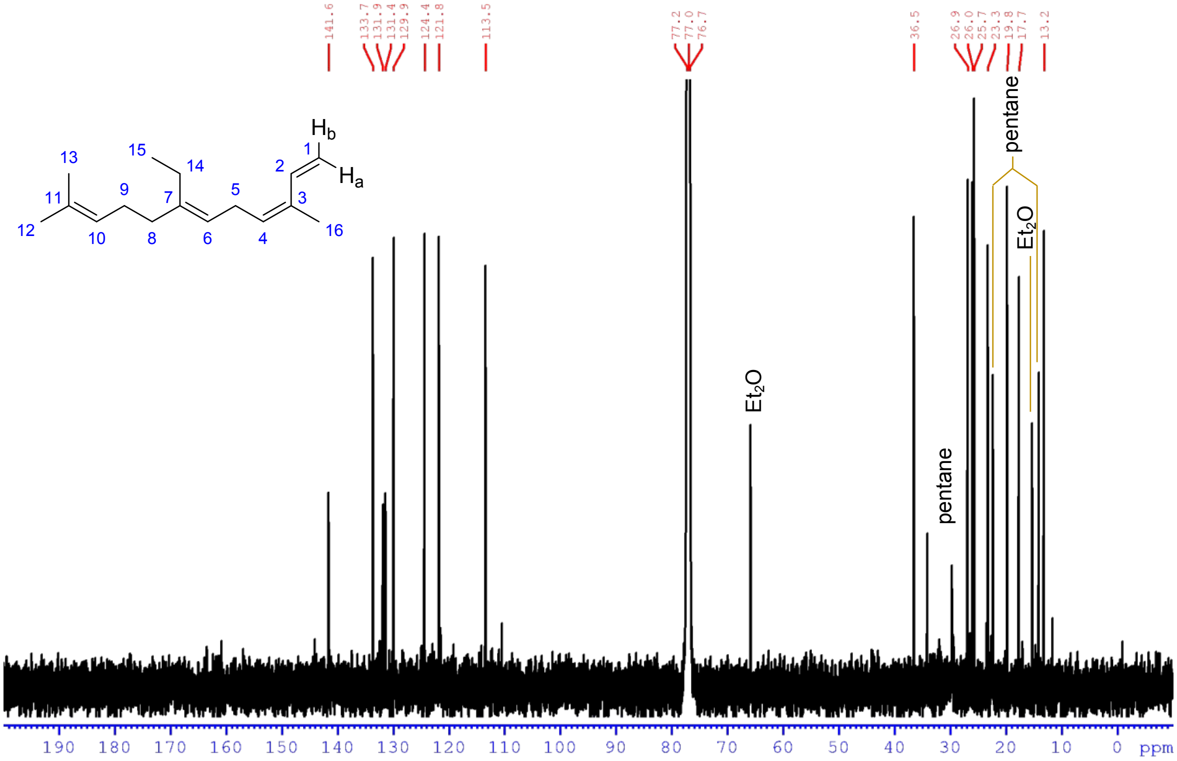


$^{1}H$-$^{13}C$ HSQC (500 MHz, $CDCl_3$) **1b**

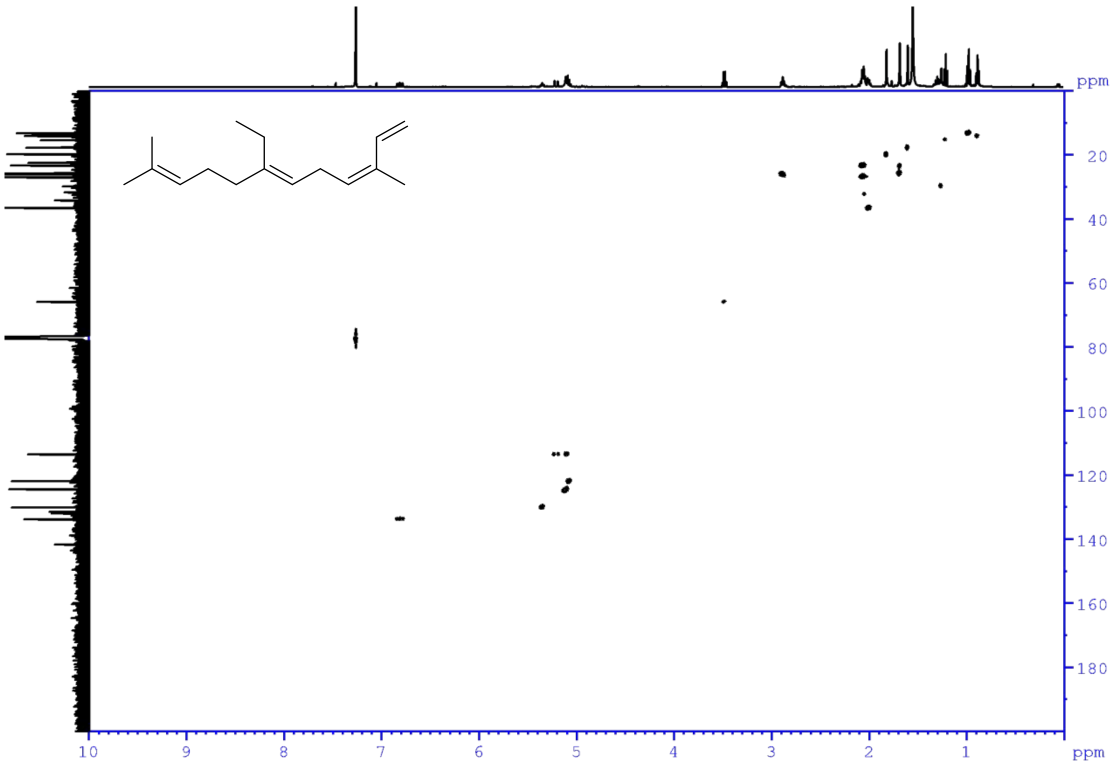

$^{1}H$-$^{13}C$ HMBC (500 MHz, $CDCl_3$) **1b**

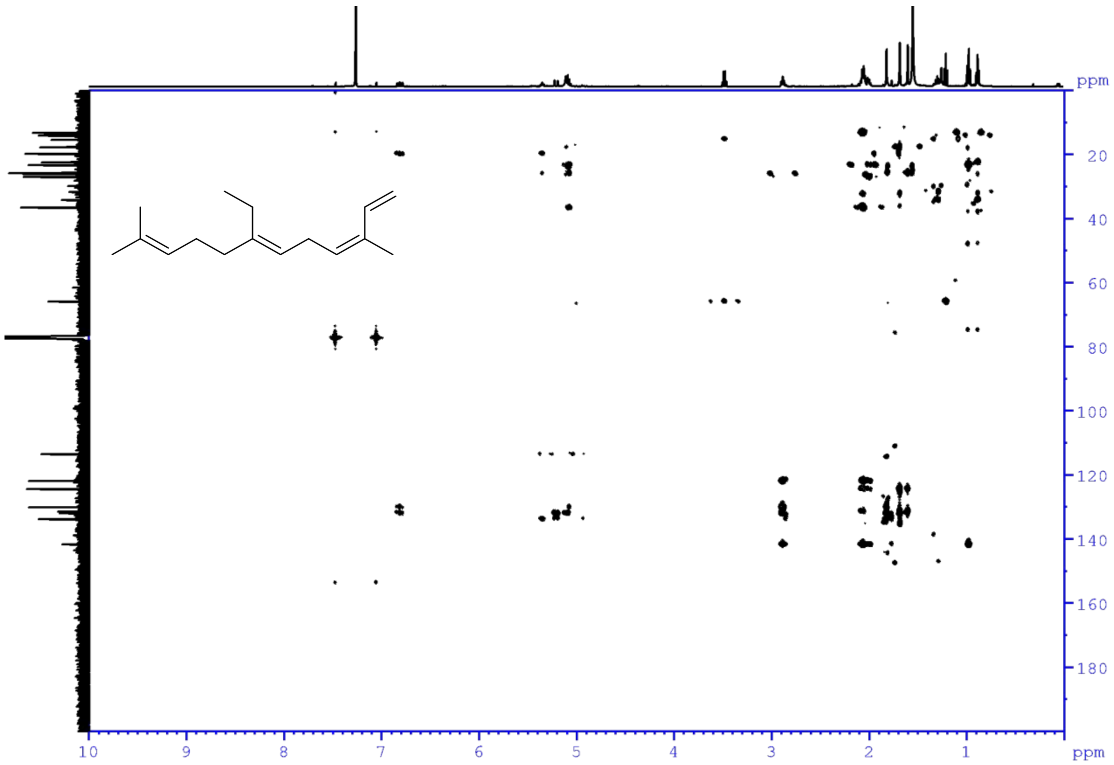


$^{1}H$-$^{1}H$ ROESY (500 MHz, $CDCl_3$) **1b**

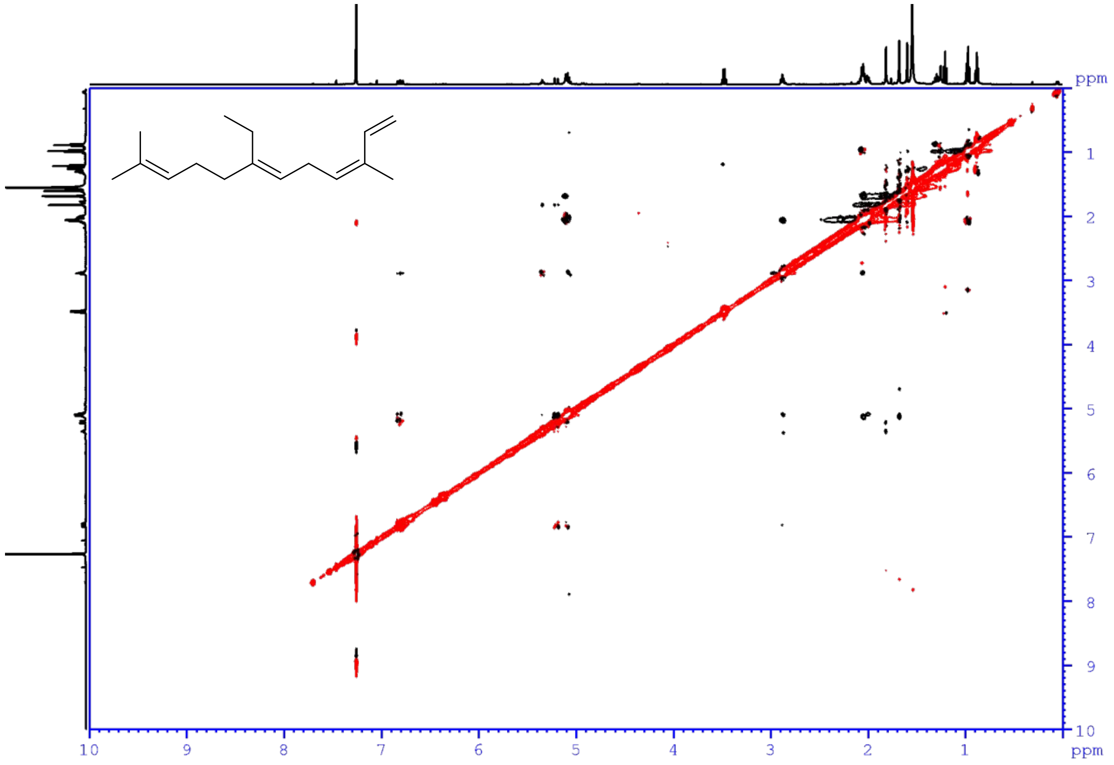

## References


1. Powell, R.W.; Buteler, M.P.; Lenka, S.; Crotti, M.; Santangelo, S.; Burg, M.J.; Bruner, S.; Brenna, E.; Roitberg, A. E.; Stewart, J. D. Investigating *Saccharomyces cerevisiae* alkene reductase OYE3 by substrate profiling, X-ray crystallography and computational methods. *Catal. Sci. Technol.*, **2018**, *8* (19), 5003–5016. DOI: 10.1039/c8cy00440d
2. Otera, J.; Niibo, Y.; Okuda, K. Selective synthesis of 1,3-dienic terpenes in a β-form through regioselective 1,4-elimination of allylic ethers. *Chem. Lett.*, **2006**, *15* (11), 1829–1832. DOI: 10.1246/cl.1986.1829
3. Vik, A.; James, A.; Gundersen, L-L. Screening of terpenes and derivatives for antimycobacterial activity; Identification of geranylgeraniol and geranylgeranyl acetate as potent inhibitors of *Mycobacterium tuberculosis in vitro*. *Planta Med.*, **2007**, *73* (13), 1410–1412. DOI: 10.1055/s-2007-990238
4. Liu, S.; Zhang, Q.; Ma, J.; Huo, X.; Ji, L.; He, B.; Chai, G.; Shi, Q.; Mao, J.; Xi, H.; Fan, W.; Li, S. Highly regio- and stereoselective dehydration of allylic alcohols to conjugated dienes *via* 1,4-*syn*-elimination with $H_2$ evolution. *Org. Lett.*, **2024**, *26* (25), 5306–5311. DOI: 10.1021/acs.orglett.4c01589
5. Morgan, E.D.; Thompson, L.D. Synthesis of (*Z*,*E*)- and (*Z*,*Z*)-α-farnesenes and -homofarnesenes. *J. Chem. Soc., Perkin Trans. 1.*, **1985**, 399–404. DOI: 10.1039/P19850000399
6. Dumas, F.; d'Angelo, J. A new route to *trans* 2,5-dialkylpyrrolidines. *Tetrahedron Lett.*, **1992**, *33* (15), 2005–2008. DOI: 10.1016/0040-4039(92)88125-O
7. Alonso, M.M.; Andrés, L.S.; Marrero, J.G. Formal synthesis of ferruginol methyl ether *via* an acid-catalysed intramolecular Diels–Alder cycloaddition. *Tetrahedron Lett.*, **2024**, *153*, 155362. DOI: 10.1016/j.tetlet.2024.155362
8. Rawat, D.S.; Gibbs, R.A. Synthesis of 7-substituted farnesyl diphosphate analogues. *Org. Lett.*, **2002**, *4* (18), 3027–3030. DOI: 10.1021/ol026176i
9. Grimm, J.A.A.; Zhou, H.; Properzi, R.; Leutzsch, M.; Bistoni, G.; Nienhaus, J.; List, B. Catalytic asymmetric synthesis of cannabinoids and menthol from neral. *Nature*, **2023**, *615* (7953), 634–639. DOI: 10.1038/s41586-023-05747-9
10. Wada, A.; Wang, F.; Suhara, Y.; Yamano, Y.; Okitsu, T.; Nakagawa, K.; Okano, T. Efficient synthesis and biological evaluation of demethyl geranylgeranoic acid derivatives. *Bioorg. Med. Chem.*, **2010**, *18* (16), 5795–5806. DOI: 10.1016/j.bmc.2010.07.003
11. Sen, S.E.; Ewing, G.J. Natural and unnatural terpenoid precursors of insect juvenile hormone. *J. Org. Chem.*, **1997**, *62* (11), 3529–3536. DOI: 10.1021/jo962008q
